# Summarizing attributable factors and evaluating risk of bias of Mendelian randomization studies for Alzheimer’s dementia and cognitive status: a systematic review and meta-analysis

**DOI:** 10.1186/s13643-025-02792-5

**Published:** 2025-03-13

**Authors:** Xiaoni Meng, Xiaochun Li, Meiling Cao, Jing Dong, Haotian Wang, Weijie Cao, Di Liu, Youxin Wang

**Affiliations:** 1 Department of Clinical Epidemiology, Beijing Institute of Respiratory Medicine and Beijing Chao-Yang Hospital, Capital Medical University, Beijing, 100020 China; 2 School of Public Health, Capital Medical University, Beijing, 100069 China; 3 The Medical Department, Xijing Hospital, the Fourth Military Medical University, Xi’an, 710032 Shaanxi China; 4 Health Management Center, Xuanwu Hospital, Capital Medical University, Beijing, 100050 China; 5 Centre for Precision Medicine, Edith Cowan University, Perth, WA 7027 Australia; 6 Shenzhen Institute of Advanced Technology, Chinese Academy of Sciences, University Town, 1068 Xueyuan Avenue, Nanshan District, Shenzhen, 518055 China; 7 School of Public Health, North China University of Science and Technology, 21 Bohaidadao, Caofeidian District, Tangshan, 063210 China

**Keywords:** Alzheimer’s dementia, Cognitive status, Modifiable factors, Mendelian randomization, Systemic review, Risk of bias

## Abstract

**Background:**

No effective treatment is available to delay or reverse the onset and progression of Alzheimer’s dementia (AD). Mild cognitive impairment, a clinical state between normal aging and AD, may offer the proper window for AD intervention and treatment. This systematic review aimed to summarize evidence from Mendelian randomization (MR) studies exploring factors attributable to AD and related cognitive status and to assess its credibility.

**Methods:**

We searched PubMed, Embase, MEDLINE, and the Cochrane Library to identify MR studies investigating the associations between any factor and AD and related cognitive status. The risk of bias in MR studies was evaluated using nine signaling questions tailored to identify potential biases based on the STROBE-MR guidelines.

**Results:**

A total of 125 eligible publications were examined, including 106 AD-related MR studies reporting 674 records and 28 cognition-related MR studies reporting 141 records. We identified 185 unique causal risk factors for AD and 49 for cognitive status. More than half of the MR studies reporting AD or cognitive status outcomes exhibited poor methodological quality, with a high risk of bias observed in 59% of the AD-related studies and 64% of the cognitive-related studies.

**Conclusions:**

This systematic review summarized modifiable factors and omics signatures, providing a database of MR studies on AD and related cognitive status. The evaluation of bias risk in MR studies serves to raise awareness and improve overall quality. A critical appraisal checklist for assessing the risk of bias may pave the way for the development of a standardized tool.

**Systematic review registration:**

The review protocol was registered with the Prospective Register of Systematic Reviews (PROSPERO) under the registration number CRD42023213990.

**Supplementary Information:**

The online version contains supplementary material available at 10.1186/s13643-025-02792-5.

## Introduction

Alzheimer’s dementia (AD) is a complex, multifactorial neurodegenerative disorder, with an estimated heritability of 58–79% [1–4]. Increasing evidence indicates that both genetic and lifestyle factors contribute to individual risk of AD [2, 5, 6]. Due to prolonged life expectancy and an aging population worldwide, AD has become a significant public health issue [7–9]. There is currently no effective treatment to delay or reverse the onset and progression of AD, and drug development has repeatedly failed [8, 10]. Mild cognitive impairment, an intermediate clinical state between normal aging and AD, may offer an important window for the intervention and treatment of AD [11, 12]. Significant gaps remain in understanding the complexity of AD and related cognitive states, highlighting the urgent need to elucidate the etiology of AD and associated cognitive performance.

Mendelian randomization (MR) uses genetic variants as instrumental variables (IVs) and leverages the fact that genotypes are randomly assorted during meiosis, thus creating a natural randomized controlled trial (RCT) [13, 14]. This approach provides stronger and more compelling evidence for the causal effects of exposure on diseases, effectively overcoming traditional limitations from confounding and reverse causality in observational studies [15–17]. Therefore, MR studies are preferred for causal inference when RCTs are not feasible. Despite numerous MR studies exploring the causal associations of risk factors with AD and related cognitive performance [18–22], the comprehensive aggregation of the etiology of AD and related cognitive performance remains limited. Furthermore, MR studies have provided important new insights into the etiology of genetic variants to AD and related cognitive performance while making reliable conduct and interpretation of MR analyses even more complex. Assessing bias in MR studies is crucial for interpreting their findings. However, the assessment of risk bias in MR studies concerning AD and related cognitive performance remains limited.

Over the past 5 years, advances in reporting the results of MR have made findings from MR studies more interpretable [16]. The STROBE-MR guidelines (Strengthening the Reporting of Observational Studies in Epidemiology using Mendelian Randomization) published in JAMA and BMJ in 2021 [23, 24], which assisted readers, reviewers, and journal editors in evaluating the quality of published MR studies. Shortcomings in study design, IV selection, data analysis, and finding extrapolation might introduce a high risk of bias, leading to deviated estimates of the clinical translation of the findings. However, tools for evaluating the risk of bias in estimates from MR studies are still limited. Therefore, the purpose of our study is to summarize current findings from MR studies to assess potential causal risk factors for AD and related cognitive performance. Another purpose of our study is to evaluate the risk of bias in MR studies in the field of AD and related cognitive status to help raise awareness and improve quality. Additionally, a critical appraisal checklist that can be used to assess the risk of bias in MR studies may pave the way for developing a comprehensive tool for assessing the risk of bias in MR studies.

## Methods

This systematic review followed the guidelines outlined in the Preferred Reporting Items for Systematic Reviews and Meta-Analyses (PRISMA). The review protocol was registered with the Prospective Register of Systematic Reviews (PROSPERO) under the registration number CRD42023213990.

### Search strategy and selection criteria

First, we systematically searched Embase, MEDLINE, and the Cochrane Library from inception to February 22, 2022, to identify MR studies examining the associations of risk factors with AD and related cognitive status. Since PubMed includes all MEDLINE literature and additional literature not indexed in MEDLINE, we further used PubMed to ensure a comprehensive search. Cognitive-related outcomes were described as cognitive status, cognitive function, cognitive performance, cognitive dysfunction, cognitive impairment, and cognitive decline. This process was independently completed by two reviewers (XM and XL) and checked by another reviewer (DL). Any disagreements were discussed and resolved by a consensus discussion among the reviewers.

The search strategies used the keywords “(“Alzheimer”* OR “cognitive” OR “cognition”) AND (“genetic instrumental” OR “genetic instrument” OR “Mendelian randomization” OR “Mendelian randomization” OR “instrumental variable”),” with detailed search strategies presented in Table S1. The full texts of potentially eligible articles were retrieved and abstracted with English language restriction. The retrieved articles were exported to the EndNote reference library (version X9.3.3), where duplicates were identified and removed. Titles and abstracts of the remaining articles were screened using predefined criteria. Subsequently, the full texts of the remaining articles were assessed to determine their inclusion.

For this evidence, we considered studies that used MR to investigate the association between any factor and AD and related cognitive status. Specifically, we excluded studies that examined associations between related exposures without genetic variants and the risk of cognition and AD, as well as those focusing on AD age at onset, progression of AD, or severity of symptom relapse or remission of AD. Studies involving non-genetic variation in IVs and genetic studies (such as genetic correlation analysis, transcriptome-wide association study, proteome-wide association study (PWAS)) other than MR studies were excluded. Additionally, studies that investigated the effect of IVs on outcomes but did not quantify causation or reported uninterpretable results were excluded. We further excluded narrative reviews, opinions, protocols, and conference abstracts. Of note, for studies with partially repeated sample sizes that reported associations of the same exposure and outcome, we retained these studies. Because sample overlap may introduce bias, we need to assess the impact of this bias on the results. For the cases where only exposure or outcome were repeated, we retained these studies to perform the meta-analysis.

### Data extraction and statistical analysis

For each eligible MR study, we extracted the following information: title, first author’s name, year of publication, investigated exposure factors, number of included single-nucleotide polymorphisms (SNPs), database sources, sample sizes for exposures and outcomes, ancestry of the study population, outcome ascertainment, and summary effect size (odds ratio [OR] or regression coefficient [β] with 95% confidence intervals [CIs]). Regarding MR effect sizes, we prioritized results from the inverse-variance weighted (IVW) method; if IVW results were unavailable, we extracted the MR‒Egger method results. Additionally, we extracted data on the detailed genetic instruments, the variance of the factor explained by the genetic instruments (*R*^2^) and *F*-statistics. If an article investigated more than one risk factor, we extracted those data separately.

We performed a meta-analysis of studies in which the same exposure associated with an outcome (i.e., AD or cognitive status) was reported more than twice using an incompletely replicated study population. For non-independent multiple MR estimates, we combined IVW estimates using two-level meta-analysis models via the rma.mv function (R version 4.2.1, package “*metafor*”) [25], which was used to address the problem that exposure or outcome summary data were used twice or more. For non-overlapping data of multiple MR estimates, we combined the IVW method results of the MR studies for the same factor and the same outcome using meta-analytic techniques (R version 4.2.1, package “*meta*”). The combined results are presented as ORs with 95% CIs. Random-effects models were used if heterogeneity existed (*P* < 0.05); otherwise, a fixed-effects model was applied. The heterogeneity of studies was evaluated using the *I*^2^ statistic, with *I*^2^ values less than 25% regarded as low heterogeneity, between 25 and 50% as mild heterogeneity, between 50 and 75% as moderate heterogeneity, and greater than 75% as severe heterogeneity. A two-sided *P* < 0.05 was considered to indicate statistical significance in all cases.

### Risk of bias assessment

Currently, no specific tool exists for assessing the risk of bias in MR studies. As such, we synthetically extracted nine possible biases based on the STROBE-MR guideline [23]. These biases span three domains: (1) bias of IV selection: weak instrument bias, pleiotropy bias, and biological complexity explained; (2) bias of population selection: sample overlap and crowd stratification; and (3) bias in the selection of the reported result: consistent with sensitivity analyses, repeatability, other study design evidence, and reporting bias. Each bias is detailed in Table S2. Each domain was judged as having a low, moderate (including cases with no information), or high risk of bias. If all domains were assessed as low risk, the final overall evaluation was low risk; if any domain was assessed as high risk, the final overall evaluation was high risk. In all other cases, the final overall evaluation was medium risk.

### Patient and public involvement

No patients or the public were involved in the design, conduct, reporting, or dissemination plans of our research, as our data were derived from previously published studies.

## Results

### Literature search and study characteristics

**Fig. 1:**
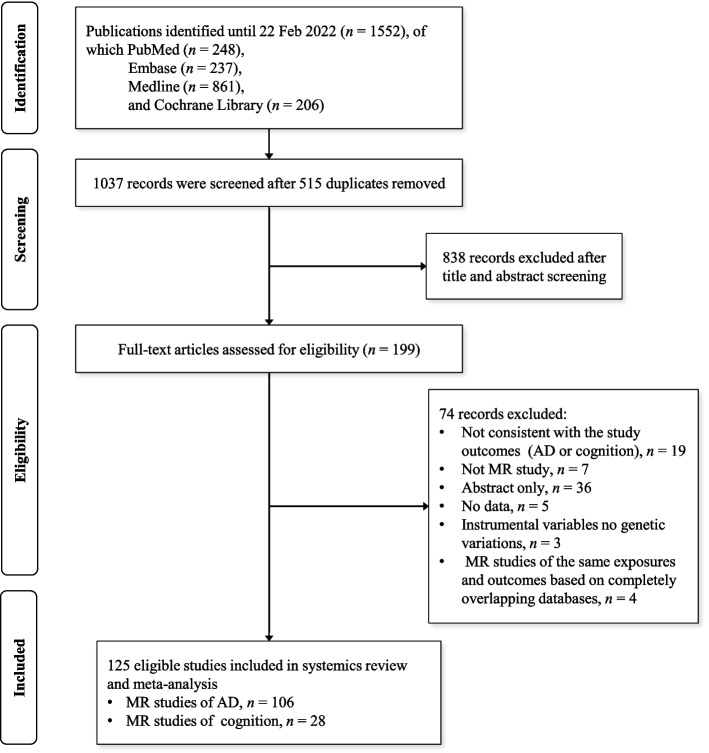
Flow diagram of studies included in the systematic review and meta-analysis. AD, Alzheimer’s dementia; MR, Mendelian randomization

In this systematic review, 1552 studies were initially identified. After removing 515 duplicates, 1037 studies remained. Subsequently, 838 studies were excluded based on title and/or abstract for not meeting eligibility criteria, leaving 199 studies. Following a full-text review, 70 studies were excluded because they were not MR studies, were abstracts only, had IVs with no genetic variations, or did not assess related outcomes (i.e., AD or cognition). Thus, 129 MR studies underwent full-text screening for data extraction. Among these, 4 articles were excluded due to complete database overlap in studies of the same exposures and outcomes. Ultimately, this review included 125 eligible studies, consisting of 106 MR studies on AD and 28 MR studies on cognitive status. The detailed process of the systematic review is presented in a schematic flowchart in Fig. 1.

### The identified MR studies for AD

The eligible articles were published between 2014 and 2022. The characteristics of the included MR studies are shown in Table S3, with all studies reporting AD as an outcome. A total of 106 eligible AD-related MR studies corresponding to 674 records examined associations of 399 unique exposures with AD, including 22 records for eight sociodemographic factors, 83 for 40 lifestyle attributes, 39 for 13 anthropometrics, 99 for 64 predispositions to diseases/phenotypes, 55 for 30 dietary intake and nutrients, 63 for 29 biochemical indexes, 115 for 54 inflammatory factors, 16 for 11 biomarkers of immunity and inflammation, and 182 for 150 omics traits (Table S3).

All 674 eligible records used data from published literature, and 610 of these records (90.64%) included AD and exposure data from European ancestry. In the included AD-related MR studies, the diagnosis of AD was mainly based on the National Institute of Neurological and Communicative Disorders and Stroke and the Alzheimer’s Disease and Related Disorders Association (NINCDS-ADRDA) criteria or the Diagnostic and Statistical Manual of Mental Disorders, 4th Edition (DSM-IV) criteria. We observed that three databases were most frequently used among the 674 associations. Specifically, the combined database of the Alzheimer’s Disease Working Group of the Psychiatric Genomics Consortium (PGC-ALZ), the International Genomics of Alzheimer’s Project (IGAP), the Alzheimer’s Disease Sequencing Project (ADSP), and the UK Biobank (UKB) [26] was used 241 times (35.76%); the IGAP database, including the Alzheimer’s Disease Genetics Consortium (ADGC), the Cohorts for Heart and Aging Research in Genomic Epidemiology consortium (CHARGE), the European Alzheimer’s Disease Initiative (EADI), and the Genetic and Environmental Risk in Alzheimer’s Disease Consortium (GERAD) [27] was used 165 times (24.48%); additionally, another IGAP database including the ADGC, CHARGE, EADI, and GERAD/Defining Genetic, Polygenic and Environmental Risk for Alzheimer’s Disease Consortium (PERADES) [3] was used 124 times (18.40%) (Table S3).

### The causality of risk/protective factors with AD

In total, 232 of 674 associations (34.42%), including 185 unique exposures from 53 articles, presented a nominally statistically significant effect (*P* < 0.05) (Table S4). In detail, among the 185 risk/protective factors associated with AD, four sociodemographic factors (including elevated years of schooling, college/university, intelligence, and occupational attainment) were associated with a reduced risk for AD. Regarding ten lifestyle attributes, there was suggestive evidence for potential associations between genetically predicted higher number of sleep episodes, sleep efficiency, smoking quantity, regular participation in sports clubs/gym, number of sexual partners, risk-taking tendency, and computer use and lower odds of AD; conversely, short sleep duration, Alcohol Use Disorder Identification Test scores, and moderate-to-vigorous physical activity were associated with higher odds of AD. For seven anthropometrics, genetically predicted increases in waist circumference (WC) and adult body mass index (BMI) were significantly associated with a higher risk of AD, while increases in height, systolic blood pressure (SBP), diastolic blood pressure (DBP), extreme height, and birth weight were significantly associated with a lower risk of AD. For 21 predispositions to diseases/phenotypes, 11 genetically predicted diseases/phenotypes (e.g., rheumatoid arthritis (RA), cognitive ability, cancers, shingles) were associated with lower odds of AD, whereas 10 diseases/phenotypes (e.g., coronary artery disease (CAD), schizophrenia, insulin resistance, herpesvirus infections) presented a risk causal effect on AD (Table S4).

Furthermore, for eight dietary intake and nutrients, 12 MR studies supported the protective causal effect of high levels of serum calcium, copper, and circulating glutamine, vitamin C, and 25-hydroxyvitamin D (25(OH)D) on AD and the risk causal effect of coffee consumption, higher linoleic acid, and isoleucine on AD. For 25 biomarkers (including 17 biochemical indices, four inflammatory cytokines, and four biomarkers of immunity and inflammation), MR studies provided suggestive evidence that 16 biochemical indices and inflammatory cytokines (e.g., 1-standard deviation [SD] increase in lipoprotein(a) [Lp(a)] concentrations and thyrotropin [TSH], elevated interleukin [IL]10 and IL1ra) were associated with a reduced risk of AD, whereas nine biomarkers (e.g., glycoprotein acetyls, CD4 count, MIP-1b, C-reactive protein (CRP), and CD 33) were associated with a high risk of AD. Some evidence for lipids and AD risk suggested that higher levels of high-density lipoprotein cholesterol (HDL-C) and 11 lipid subclasses (e.g., total cholesterol [TC] in large HDL-C, mean diameter for HDL particles, free cholesterol in large HDL) were associated with a lower risk of AD, others demonstrated that greater low-density lipoprotein cholesterol (LDL-C) and TC levels were positively associated with the risk of AD (Table S4).

In addition, 110 of the 150 druggable targets (73.33%), including gut metabolites and microbiota, neuroimaging features, leukocyte telomere length, drugs, proteins, genes, and DNA methylation targets, were causally associated with AD. In detail, an MR study reported suggestive associations of a host genetic-driven increase in Blautia and elevated γ-aminobutyric acid, a downstream product of Blautia-dependent arginine metabolism, with a lower risk of AD [28]. In addition, the MR study revealed suggestive associations between cortical surface area (per 1 SD increase in the lateral orbitofrontal, supramarginal, and lingual regions, and per 1 SD decrease in temporal pole) and decreased thickness of the cuneus with a higher risk of AD [29]. Additionally, MR evidence suggested that shorter telomere length was causally associated with a higher risk of AD [30–33]. MR studies indicated that the use of lipid-lowering drugs (PCSK9 inhibitors) was associated with an increased risk of AD [34], whereas the overall use of antihypertensive medications and reduction in SBP through variants in genes encoding targets of calcium channel blockers were associated with a lower risk of AD [35]. The expression of various genes was also causally associated with the risk of AD, confirming *ACE* and *BIN1* as risk genes for AD [36, 37], while a genetically predicted anti-obesity drug target gene *CNR1* was a protective factor against AD risk [38]. Protein targets of angiotensin-converting enzyme inhibitors were associated with increased AD risk [39], and 11 other AD PWAS-significant genes (e.g., *PVR*, *EPHX2, SNX32*, *CTSH*) were consistent with a causal role in AD, acting via their cis-regulated brain protein abundance [19]. Furthermore, summary data-based MR (SMR) method integrating tissue-shared and tissue-specific protein quantitative trait loci (pQTLs) data identified proteins involved in AD risk in neurologically relevant tissues (brain, cerebrospinal fluid, and plasma), showing that variants in the *CD33* locus were associated with plasma CD33 protein levels and AD risk [18]. Network SMR applied to cis-DNA methylation quantitative trait loci (mQTLs) data revealed pleiotropic association of some novel DNA methylation sites and genes with AD (e.g., cg05656486 (*NDUFS2*), cg13210467 (*STAG3*), and cg03887787 (*FIBP*)) [40]. These identified genes, proteins, and DNA methylation loci have the potential to serve as attractive drug targets, meriting further investigation through functional studies and clinical trials to illustrate the value of these studies in the discovery of drug targets for AD (Table S4).

A total of 399 unique AD-related risk factors were considered, with 142 studied in more than one MR estimate. Meta-analyses of 417 associations from 86 eligible AD-related MR studies reported links between 142 unique exposures and AD (Table S4). Results indicated that 10 of the 142 meta-analyses (5.63%) showed a nominally statistically significant effect (*P* < 0.05), including educational attainment/years of schooling, intelligence, DBP, RA, circulating 25(OH)D, LDL-C, TSH, Lp(a), CRP, and leukocyte telomere length (Table 1). Overall, 98 of the 142 meta-analyses (69.01%) reported associations with low between-study heterogeneity estimates (*I*^2^ < 25%), 10 (7.04%) with mild heterogeneity estimates (25% ≤ *I*^2^ < 50%), 18 (12.68%) with moderate heterogeneity estimates (50% ≤ *I*^2^ < 75%), and 15 (10.56%) with very large (severe) heterogeneity estimates (*I*^2^ ≥ 75%). A meta-analysis investigating the causality between linoleic acid and AD showed that *I*^2^ included null values, possibly due to both point and interval estimates of the effect value of one MR study being 1 (Table S4).

**Table 1:** The significant association of AD with risk facto﻿rs

Publication	Risk of factor (exposure)	OR (95% CI)	*P*
	**Sociodemographic factors**		
Meta-analysis	Educational attainment/years of schooling	0.755 (0.658–0.866)	0.001
Meta-analysis	Intelligence	0.633 (0.491–0.816)	0.011
Ko et al. 2021	Occupational attainment	0.720 (0.540–0.950)	0.020
	**Lifestyle attributes**		
Yang et al. 2020	Number of sexual partners	0.500 (0.270–0.930)	0.040
Andrews et al. 2021	Moderate-to-vigorous physical activity	1.320 (1.030–1.710)	0.038
Yang et al. 2021	Computer use	0.670 (0.480–0.920)	0.010
	**Anthropometrics**		
Meta-analysis	DBP	0.989 (0.980–0.999)	0.035
Li et al. 2020	Extreme height	0.996 (0.992–0.999)	0.015
Li et al. 2021	Birth weight	0.970 (0.940–1.000)	0.038
	**Predisposition to diseases/phenotypes**		
Han et al. 2018	α-synuclein in PD	0.638 (0.485–0.838)	0.001
Jansen et al. 2019	Schizophrenia	1.016 (1.004–1.028)	0.012
Zhou et al. 2021	IR: based on fasting insulin	1.130 (1.039–1.228)	0.004
Zhou et al. 2021	IR: based on the euglycemic-hyperinsulinemic clamp and the insulin suppression test	1.015 (1.001–1.029)	0.030
Walter et al. 2016	Insulin sensitivity	1.170 (1.020–1.340)	0.020
Seddighi et al. 2019	Lung cancer	0.910 (0.840–0.990)	0.019
Seddighi et al. 2019	Smoking-related cancers: including renal cell carcinoma, pancreatic cancer, upper aerodigestive tract cancer, urinary bladder cancer, lung cancer	0.950 (0.920–0.980)	0.003
Seddighi et al. 2019	Leukemia	0.980 (0.960–1.000)	0.012
Seddighi et al. 2019	Breast cancer	0.940 (0.890–0.990)	0.028
Seddighi et al. 2019	Non-smoking-related cancers: including prostate cancer, leukemia, breast cancer, melanoma, lymphoma, ovarian cancer	0.980 (0.970–0.997)	0.009
Seddighi et al. 2019	All cancers: including renal cell carcinoma, pancreatic cancer, upper aerodigestive tract cancer, urinary bladder cancer, lung cancer, prostate cancer, leukemia, breast cancer, melanoma, lymphoma, ovarian cancer)	0.980 (0.960–0.990)	2.70 × 10^−4^
Meta-analysis	RA	0.991 (0.982–0.999)	0.039
	**Dietary intake**		
Cheng et al. 2019	Copper	0.870 (0.750–1.000)	0.050
Adams 2020	Circulating glutamine	0.830 (0.710–0.970)	0.020
Larsson et al. 2017	Isoleucine	1.350 (1.080–1.690)	0.007
Meta-analysis	25(OH)D	0.861 (0.773–0.959)	0.018
	**Biochemical index**		
Lord et al. 2021	Mean diameter for HDL particles	0.887 (0.797–0.987)	0.027
Lord et al. 2021	Total cholesterol in large HDL	0.891 (0.803–0.988)	0.029
Lord et al. 2021	Free cholesterol in large HDL	0.891 (0.808–0.983)	0.021
Lord et al. 2021	Total lipids in large HDL	0.914 (0.839–0.996)	0.040
Lord et al. 2021	Concentration of large HDL particles	0.913 (0.838–0.995)	0.038
Lord et al. 2021	Phospholipids in large HDL	0.912 (0.836–0.995)	0.039
Lord et al. 2021	Total cholesterol in very large HDL	0.883 (0.790–0.988)	0.029
Lord et al. 2021	Free cholesterol in very large HDL	0.859 (0.783–0.943)	0.001
Lord et al. 2021	Total lipids in very large HDL	0.881 (0.801–0.968)	0.008
Lord et al. 2021	Concentration of very large HDL particles	0.866 (0.786–0.955)	0.004
Lord et al. 2021	Phospholipids in very large HDL	0.886 (0.810–0.969)	0.008
Meta-analysis	LDL	1.079 (1.012–1.150)	0.025
Meta-analysis	TSH	0.986 (0.978–0.994)	0.012
Meta-analysis	Lp(a)	0.941 (0.912–0.971)	1.0 × 10^−4^
Lord et al. 2021	Glycoprotein acetyls	1.199 (1.045–1.375)	0.010
	**Inflammatory cytokines**		
Meta-analysis	CRP	1.018 (1.005–1.031)	0.016
	**Biomarkers of immunity and inflammation**		
Yang et al. 2021	Complement C4 (Brain)	2.273 (1.560–3.312)	1.89 × 10^−5^
Fani et al. 2021	CD4 count	1.32 (1.126–1.514)	0.005
	**Omics traits**		
Zhuang et al. 2020	γ-aminobutyric acid	0.960 (0.920–1.000)	0.034
Zhuang et al. 2020	Blautia	0.880 (0.790–0.990)	0.028
Wu et al. 2021	Surface area: lateral orbitofrontal	1.040 (1.010–1.080)	0.022
Wu et al. 2021	Surface area: temporal pole	0.950 (0.900–0.997)	0.040
Wu et al. 2021	Surface area: supramarginal	1.050 (1.010–1.090)	0.008
Wu et al. 2021	Surface area: lingual	1.030 (1.004–1.060)	0.024
Wu et al. 2021	Thickness of cuneus	0.930 (0.890–0.980)	0.006
Meta-analysis	Leukocyte telomere length	1.100 (1.005–1.204)	0.041
Zhuang et al. 2021	Anti-obesity drug target genes: *CNR1*	0.950 (0.920–0.990)	0.010
Walker et al. 2020	Angintensin converting enzyme inhibitors	13.200 (2.140–81.240)	0.005
	***Genome***		
Baird et al. 2021	*CCDC6*	1.249 (1.157–1.319)	1.22 × 10^−8^
Baird et al. 2021	*TSPAN14*	1.140 (1.085–1.193)	2.64 × 10^−7^
Baird et al. 2021	*KAT8*	0.875 (0.837–0.941)	6.18 × 10^−9^
Baird et al. 2021	*ZNF646*	0.806 (0.749–0.855)	3.53 × 10^−9^
Baird et al. 2021	*CCNT2-AS1*	0.860 (0.811–0.898)	5.06 × 10^−7^
Baird et al. 2021	*PRSS36*	0.874 (0.837–0.914)	1.66 × 10^−9^
Baird et al. 2021	*AC012146.1*	0.866 (0.828–0.887)	5.62 × 10^−10^
Wingo et al. 2021	*EPHX2*	1.063 (1.037–1.088)	7.07 × 10^−7^
Wingo et al. 2021	*PVR*	0.592 (0.468–0.750)	1.41 × 10^−5^
Wingo et al. 2021	*SNX32*	0.903 (0.861–0.947)	2.73 × 10^−5^
Wingo et al. 2021	*CTSH*	1.050 (1.026–1.075)	3.06 × 10^−5^
Wingo et al. 2021	*RTFDC1*	1.140 (1.070–1.214)	4.63 × 10^−5^
Wingo et al. 2021	*LACTB*	1.098 (1.043–1.155)	3.77 × 10^−4^
Wingo et al. 2021	*ICA1L*	0.816 (0.729–0.913)	4.14 × 10^−4^
Wingo et al. 2021	*DOC2A*	0.713 (0.581–0.875)	1.21 × 10^−3^
Wingo et al. 2021	*PLEKHA1*	1.484 (1.144–1.927)	3.00 × 10^−3^
Wingo et al. 2021	*STX4*	1.593 (1.155–2.198)	4.55 × 10^−3^
Wingo et al. 2021	*STX6*	1.372 (1.080–1.742)	9.54 × 10^−3^
Wingo et al. 2021	*CARHSP1*	1.136 (1.028–1.256)	1.22 × 10^−2^
Zhu et al. 2021	*B4GALT3* (Blood)	0.911 (0.883–0.940)	7.33 × 10^−9^
Zhu et al. 2021	*NDUFS2* (Blood)	0.948 (0.930–0.967)	1.63 × 10^−7^
Zhu et al. 2021	*BIN1* (Blood)	1.043 (1.027–1.059)	2.36 × 10^−8^
Zhu et al. 2021	*HLA-DRA* (Blood)	1.174 (1.102–1.249)	3.99 × 10^−7^
Zhu et al. 2021	*CASTOR3* (Blood)	0.892 (0.856–0.930)	4.26 × 10^−8^
Zhu et al. 2021	*EPHA1-AS1* (Blood)	0.969 (0.960–0.979)	1.11 × 10^−10^
Zhu et al. 2021	*SLC24A4* (Blood)	0.979 (0.972–0.987)	7.31 × 10^−7^
Zhu et al. 2021	*RIN3* (Blood)	0.938 (0.914–0.962)	1.58 × 10^−6^
Zhu et al. 2021	*APH1B* (Blood)	1.025 (1.015–1.035)	1.22 × 10^−7^
Zhu et al. 2021	*ZNF232* (Blood)	0.944 (0.924–0.964)	1.57 × 10^−7^
Zhu et al. 2021	*SIGLEC22P* (Blood)	0.931 (0.905–0.956)	3.91 × 10^−7^
Zhu et al. 2021	*CD33* (Blood)	1.038 (1.024–1.052)	6.18 × 10^−7^
Zhu et al. 2021	*CASS4* (Blood)	1.172 (1.101–1.248)	7.20 × 10^−7^
Zhu et al. 2021	*RPL39P* (Blood)	1.112 (1.069–1.156)	1.76 × 10^−7^
	***DNA methylation***		
Liu et al. 2021	cg05656486 (*NDUFS2*) (Brain)	1.043 (1.025–1.061)	2.06 × 10^−6^
Liu et al. 2021	cg08850169 (*NDUFS2*) (Brain)	1.047 (1.027–1.068)	5.28 × 10^−6^
Liu et al. 2021	cg16673712 (*NDUFS2*) (Brain)	0.979 (0.970–0.989)	7.21 × 10^−5^
Liu et al. 2021	cg23274951 (*NDUFS2*) (Brain)	1.045 (1.025–1.066)	3.89 × 10^−6^
Liu et al. 2021	cg24049880 (*NDUFS2*) (Brain)	1.041 (1.025–1.057)	1.33 × 10^−6^
Liu et al. 2021	cg13210467 (*STAG3*) (Brain)	0.957 (0.940–0.974)	3.11 × 10^−6^
Liu et al. 2021	cg22906224 (*STAG3*) (Brain)	0.990 (0.986–0.994)	9.58 × 10^−7^
Liu et al. 2021	cg03887787 (*FIBP*) (Brain)	0.980 (0.971–0.990)	1.67 × 10^−5^
Liu et al. 2021	cg19792802 (*FIBP*) (Brain)	0.991 (0.987–0.995)	1.36 × 10^−5^
Liu et al. 2021	cg02220965 (*KAT8*) (Brain)	1.018 (1.010–1.026)	1.78 × 10^−5^
Liu et al. 2021	cg04275947 (*KAT8*) (Brain)	1.045 (1.023–1.068)	4.88 × 10^−5^
Liu et al. 2021	cg07078430 (*KAT8*) (Brain)	0.980 (0.973–0.988)	8.74 × 10^−6^
Liu et al. 2021	cg04275947 (*RNF40*) (Brain)	1.045 (1.023–1.068)	4.88 × 10^−5^
Liu et al. 2021	cg07078430 (*RNF40*) (Brain)	0.980 (0.973–0.988)	8.74 × 10^−6^
Liu et al. 2021	cg04275947 (*PRSS36*) (Brain)	1.045 (1.023–1.068)	4.88 × 10^−5^
Liu et al. 2021	cg07078430 (*PRSS36*) (Brain)	0.980 (0.973–0.988)	8.74 × 10^−6^
Liu et al. 2021	cg02220965 (*C16orf93*) (Brain)	1.018 (1.010–1.026)	1.78 × 10^−5^
Liu et al. 2021	cg04275947 (C16orf93) (Brain)	1.045 (1.023–1.068)	4.88 × 10^−5^
Liu et al. 2021	cg03433048 (*ZNF232*) (Brain)	1.017 (1.009–1.025)	5.03 × 10^−5^
Liu et al. 2021	cg20814095 (*ZNF232*) (Brain)	1.021 (1.011–1.031)	5.07 × 10^−5^
Liu et al. 2021	cg03433048 (*AC012146.7*) (Brain)	1.017 (1.009–1.025)	5.03 × 10^−5^
Liu et al. 2021	cg20814095 (*AC012146.7*) (Brain)	1.021 (1.011–1.031)	5.07 × 10^−5^
Liu et al. 2021	cg09070378 (*FCER1G*) (Blood)	1.027 (1.015–1.040)	2.17 × 10^−5^
Liu et al. 2021	cg19116668 (*PILRA*) (Blood)	1.019 (1.014–1.024)	1.26 × 10^−14^
Liu et al. 2021	cg01669108 (*FIBP*) (Blood)	0.986 (0.980–0.993)	2.33 × 10^−5^
Liu et al. 2021	cg23483894 (*FIBP*) (Blood)	0.973 (0.960–0.986)	6.12 × 10^−5^
Liu et al. 2021	cg03887787 (*CTSW*) (Blood)	0.991 (0.988–0.995)	4.65 × 10^−6^
Liu et al. 2021	cg01669108 (*CTSW*) (Blood)	0.986 (0.980–0.993)	2.33 × 10^−5^
Liu et al. 2021	cg23483894 (*CTSW*) (Blood)	0.973 (0.960–0.986)	6.12 × 10^−5^
Liu et al. 2021	cg17207590 (*APH1B*) (Blood)	0.966 (0.950–0.982)	3.40 × 10^−5^
Liu et al. 2021	cg26675395 (*BCKDK*) (Blood)	1.055 (1.029–1.082)	3.29 × 10^−5^
Liu et al. 2021	cg00249205 (*BCKDK*) (Blood)	0.972 (0.960–0.985)	3.04 × 10^−5^
Liu et al. 2021	cg26949037 (*BCKDK*) (Blood)	0.960 (0.941–0.980)	8.67 × 10^−5^
Liu et al. 2021	cg05768032 (*BCKDK*) (Blood)	0.990 (0.985–0.994)	3.09 × 10^−5^
Liu et al. 2021	cg10421029 (*BCKDK*) (Blood)	1.033 (1.018–1.048)	1.39 × 10^−5^
Liu et al. 2021	cg02220965 (*BCKDK*) (Blood)	1.016 (1.009–1.022)	6.52 × 10^−6^
Liu et al. 2021	cg03418659 (*BCKDK*) (Blood)	1.016 (1.009–1.023)	8.46 × 10^−6^
Liu et al. 2021	cg01067137 (*BCKDK*) (Blood)	1.017 (1.009–1.026)	3.29 × 10^−5^
	***Proteome***		
Yang et al. 2021	Tyrosine-protein phosphatase nonreceptor type 1 (CSF)	0.003 (3.81 × 10^−4^–0.023)	2.74 × 10^−8^
Yang et al. 2021	SLAM family member 5 (CSF)	0.091 (0.044–0.189)	1.17 × 10^−10^
Yang et al. 2021	Endothelial monocyte-activating polypeptide 2 (Plasma)	0.043 (0.027–0.069)	3.70 × 10^−38^
Yang et al. 2021	SPARC-like protein 1 (Plasma)	20.934 (6.176–70.954)	1.04 × 10^−6^
Yang et al. 2021	Hemopexin (Plasma)	2.288 (1.652–3.169)	6.28 × 10^−7^
Yang et al. 2021	Sialic acid-binding Ig-like lectin 14 (Plasma)	0.227 (0.132–0.390)	8.05 × 10^−8^
Yang et al. 2021	Sialic acid-binding Ig-like lectin 6 (Plasma)	0.152 (0.065–0.355)	1.37 × 10^−5^
Yang et al. 2021	Junctional adhesion molecule-like (Plasma)	1.939 (1.422–2.645)	2.87 × 10^−5^
Yang et al. 2021	cAMP-specific 3',5'-cyclic phosphodiesterase 4D (Plasma)	43.143 (9.937–187.306)	5.02 × 10^−7^
Yang et al. 2021	Cathepsin F (Plasma)	11.637 (3.632–37.290)	3.62 × 10^−5^
Yang et al. 2021	Kallikrein-13 (Plasma)	0.102 (0.042–0.251)	6.69 × 10^−7^
Yang et al. 2021	Ephrin type-A receptor 5 (Plasma)	0.154 (0.080–0.297)	2.20 × 10^−8^
Yang et al. 2021	Cyclin-dependent kinase inhibitor 1B (Plasma)	0.202 (0.101–0.404)	6.01 × 10^−6^
Yang et al. 2021	Ficolin-2 (Plasma)	0.163 (0.070–0.381)	2.73 × 10^−5^
Yang et al. 2021	Cytokine receptor-like factor 1: Cardiotrophin-like cytokine factor 1 Complex (Brain)	0.003 (2.12 × 10^−4^–0.048)	3.86 × 10.8^−5^
Yang et al. 2021	Copine-1 (Brain)	0.440 (0.296–0.654)	4.91 × 10^−5^
Yang et al. 2021	Leucine-rich repeat transmembrane neuronal protein 1 (Brain)	0.007 (0.001–0.058)	3.62 × 10^−6^
Yang et al. 2021	A disintegrin and metalloproteinase with thrombospondin motifs 4 (Brain)	197,773.744 (11,653.427–3,356,476.468)	3.14 × 10^−17^
Yang et al. 2021	Ras GTPase-activating protein 1 (Brain)	187.888 (24.381–1447.932)	5.02 × 10^−7^
Yang et al. 2021	Scavenger receptor cysteine-rich type 1 protein M130 (Brain)	0.021 (0.004–0.114)	7.30 × 10^−6^

*CI*, confidence interval; *CSF*, cerebrospinal fluid; *CRP*, C-reactive protein; *DBP*, diastolic blood pressure; *HDL*, high-density lipoprotein; *IR*, insulin resistance; *LDL*, low-density lipoprotein; *Lp(a)*, lipoprotein(a); *OR*, odds ratio; *PD*, Parkinson’s disease; *RA*, rheumatoid arthritis; *TSH*, thyrotropin; *25(OH)D*, 25-hydroxyvitamin D

Specifically, 11 records from 10 MR studies investigated the causal association between years of schooling and AD, with six (54.55%) suggesting that increased years of schooling were associated with a lower risk of AD. The overall meta-analysis still indicated a significant association between genetically predicted more years of schooling and reduced risk of AD (OR 0.757, 95% CI 0.659–0.869,* P* = 0.0011, *I*^2^ = 78.94%) (Table 1 and Table S4). Similarly, a meta-analysis of higher intelligence, consisting of four records from three MR studies, revealed that intelligence was causally associated with a reduced risk of AD (OR 0.633 95% CI 0.491–0.816),* P* = 0.0105, *I*^2^ = 0.00%) (Table 1 and Table S4). Furthermore, meta-analyses of five, four, one, two, six, five, and five eligible MR studies consisting of six, five, four, two, 10, seven, and seven records, respectively, reported that genetically increased DBP (OR 0.989, 95% CI 0.980–0.999, *P* = 0.035, *I*^2^ = 28.46%), 25(OH)D (OR 0.861, 95% CI 0.773–0.959, *P* = 0.0181, *I*^2^ = 49.25%), TSH (OR 0.986, 95% CI 0.978–0.994, *P* = 0.0123, *I*^2^ = 0.00%), and Lp(a) (OR 0.941, 95% CI 0.912–0.971, *P* < 0.001, *I*^2^ = 0.00%) levels had a protective effect on AD. In contrast, genetically elevated LDL-C (OR 1.079, 95% CI 1.012–1.150, *P* = 0.0249, *I*^2^ = 55.96%), CRP (OR 1.018, 95%CI 1.005–1.031, *P* = 0.0163, *I*^2^ = 22.45%), and short telomeres (OR 1.100, 95% CI 1.005–1.204, *P* = 0.0413, *I*^2^ = 66.17%) were associated with an increasing risk of AD (Table 1 and Table S4). Moreover, meta-analyses of four reports supported that RA (OR 0.991, 95% CI 0.982–0.999, *P* = 0.039, *I*^2^ = 38.25%) was a causal protective factor for AD risk (Table 1 and Table S4).

### The identified MR studies for cognition-related outcomes

Among eligible cognition-related MR studies, 28 studies corresponded to 141 records examining associations of 49 unique risk factors with cognition status. These included two records for two sociodemographic factors, 25 for eight lifestyle attributes, 13 for six anthropometrics, 23 for 14 predispositions to diseases/phenotypes, 13 for three dietary intake and nutrients, 49 for eight biochemical indexes, one for one biomarker of immunity and inflammation, and 15 for seven omics traits. The cognition-related outcomes included general cognitive function, cognitive resilience, and mild cognitive impairment, as well as specific domains of cognition, such as visual memory, reaction time, working memory, response inhibition, and emotion recognition, assessed using various neuropsychological tests (Table S5).

The two databases related to cognitive performance/cognition function were used the most frequently. First, the cognitive performance dataset from the Social Science Genetic Association Consortium (SSGAC) is the most commonly used database for assessing general cognitive function, appearing 14 times (9.93%) in this systematic review. The second most frequently used database was the genome-wide association study (GWAS) of cognitive function from Davies et al. 2018, which comprised three databases: CHARGE, Cognitive Genomics Consortium (COGENT), and UKB. It was used 11 times (7.80%), with seven studies utilizing CHARGE and COGENT, and four using data from all three databases combined. Additionally, out of the total 141 associations, 60 associations (44.55%) utilized cognitive function data from the UKB, focusing on specific domains of cognition such as visual memory, reaction time, pair matching, reasoning, prospective memory, and verbal-numeric reasoning (fluid intelligence). Moreover, almost all of the included cognition-related MR studies were conducted with data on cognitive status and exposures based on European ancestry (Table S5).

### The causality of risk/protective factors with cognitive status

Among the 141 associations examined, 34 (24.11%) involved 24 factors associated with cognitive function (Table S6). Of the 24 risk/protective factors related to cognition, six cognition-associated lifestyle attributes were identified, namely, each additional hour/day of sleep, smoking initiation, lifetime cannabis use, television watching, and driving behavior, which were causally associated with increased risk for various cognitive statuses, contributing to lower reaction time, lower fluid intelligence and cognitive ability, worse working memory, and decreased cognitive performance. In contrast, computer use was a beneficial factor for cognitive status. Furthermore, four anthropometric indicators, namely, birth weight, SBP, DBP, and pulse pressure (PP), were causally associated with cognition. MR evidence showed that high birth weight was a protective factor for intelligence. Genetically predicted SBP, DBP, or PP was causally associated with poorer processing speed (i.e., Digit Symbol Substitution Test score), and increased SBP level was associated with poorer verbal memory (i.e., Rey Auditory Verbal Learning Test score). However, an increment in genetically predicted SBP level was associated with higher executive function (i.e., Stroop Interference Test score). Notably, for the three cognition-associated diseases/phenotypes, there was evidence for causal associations between genetically determined schizophrenia and reduced cognitive resilience. Reduced levels of lung function indicators (forced expiratory volume in 1 s (FEV1) and forced vital capacity) were associated with lower cognitive function (Table S6).

Four biochemical indicators involving eight associations from three eligible MR studies reported that high TC levels increased the prevalence of mild cognitive impairment. Significant causal effects of mean corpuscular hemoglobin (MCH) on cognitive performance were observed, specifically in verbal-numeric reasoning and numeric memory. Similarly, red blood cell distribution width was significantly related to verbal-numeric reasoning, suggesting that anemia may have a causal effect on cognitive performance. Furthermore, MR results did not support the causal effects of serum uric acid, serum creatinine, or serum cystatin C on cognitive performance testing of verbal-numeric reasoning, reaction time, visual memory, and numeric memory, except that the urine albumin to creatinine ratio was causally associated with verbal-numeric reasoning (Table S6).

It is worth mentioning that six omics traits and one biomarker of immunity and inflammation involving eight associations from four studies were associated with cognition. Specifically, a causal effect from longer telomere length on better general cognitive performance was found, and the association between telomere length and Stroop interference score (cognitive test) was also found to be causal. MR evidence indicated that genetically predicted increased total white matter volume and cerebral white matter volume in the left and right hemispheres are causally protective factors for cognitive resilience. The DNA methylation locus (i.e., cg15676719) was linked to cognitive function in offspring, while the proteome (dipeptidase 1) and the biomarker of immunity (CD33 protein) were associated with poorer cognitive function (Table S6).

**Fig. 2:**
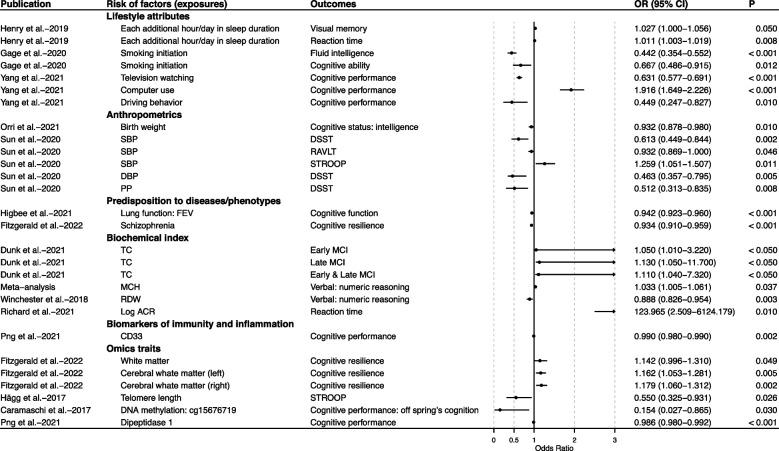
Forest plot of significant results of included MR studies for cognition. ACR, albumin to creatinine ratio; CI, confidence interval; DBP, diastolic blood pressure; DSST, Digit Symbol Substitution Test; FEV, forced expiratory volume; MCH, mean corpuscular hemoglobin; MCI, mild cognitive impairment; OR, odds ratio; PP, pulse pressure; RAVLT, Rey Auditory Verbal Learning Test; RDW, red blood cell distribution width; SBP, systolic blood pressure; STROOP, Stroop interference score; TC, total cholesterol

A total of 49 cognition-related risk factors were evaluated, with six of them (12.77%) from six eligible MR studies investigated multiple cognitive outcomes, including lifetime cannabis use, herpes simplex virus infection, MCH, FEV1, type 2 diabetes (T2D), and telomere length (Table S6). However, the meta-analysis results showed that only the association between MCH and cognitive test (i.e., verbal–numeric reasoning) was statistically significant (*P* < 0.05) (Fig. 2). Specifically, an MR study used two different cognitive databases to investigate the potential causality between lifetime cannabis use and three neuropsychological tests, namely, working memory, response inhibition, and emotion recognition. Meta-analyses for each cognition-related outcome indicated no significant association between lifetime cannabis use and any of the neuropsychological tests (OR 0.633, 95% CI 0.016–23.886, *P* = 0.359, *I*^2^ = 10.95% for working memory; OR 1.040, 95% CI 0.112–9.611, *P* = 0.861, *I*^2^ = 0.00% for response inhibition; OR 0.927, 95% CI 0.062–13.807, *P* = 0.781, *I*^2^ = 0.00% for emotion recognition) (Table S6). Similarly, meta-analyses of T2D involving more than one cognitive test (including reaction time and visual memory) also presented no statistically significant effect (*P* > 0.050) (Table S6). Moreover, the meta-analysis results of MCH showed inconsistent trends in four cognitive tests (i.e., reaction time, numeric memory, prospective memory, and verbal-numeric reasoning). Specifically, no significant causal effects were observed between MCH and reaction time, numeric memory, or prospective memory, while genetically predicted higher MCH was associated with relatively higher verbal–numeric reasoning (OR 1.033, 95% CI 1.005–1.061, *P* = 0.037, *I*^2^ = 16.59%) (Fig. 2 and Table S6), implying that anemia was associated with increased cognitive function.

Three records investigated the relationship between herpes simplex virus infection and cognitive function, and the meta-analytic results showed no significant association (OR 1.000, 95% CI 1.000–1.000, *P* > 0.999) (Table S6). Two records examined the association between FEV1 and cognitive function, and the meta-analysis also revealed no significant association (OR 0.945, 95% CI 0.697–1.282, *P* = 0.257, *I*^2^ = 0.00%) (Table S6). Finally, two records investigated the relationship between telomere length and general cognitive ability, and the meta-analysis demonstrated no significant correlation (OR 1.087, 95% CI 0.700–1.687, *P* = 0.251, *I*^2^ = 0.00%) (Table S6).

### Risk of bias assessment of MR studies

**Fig. 3:**
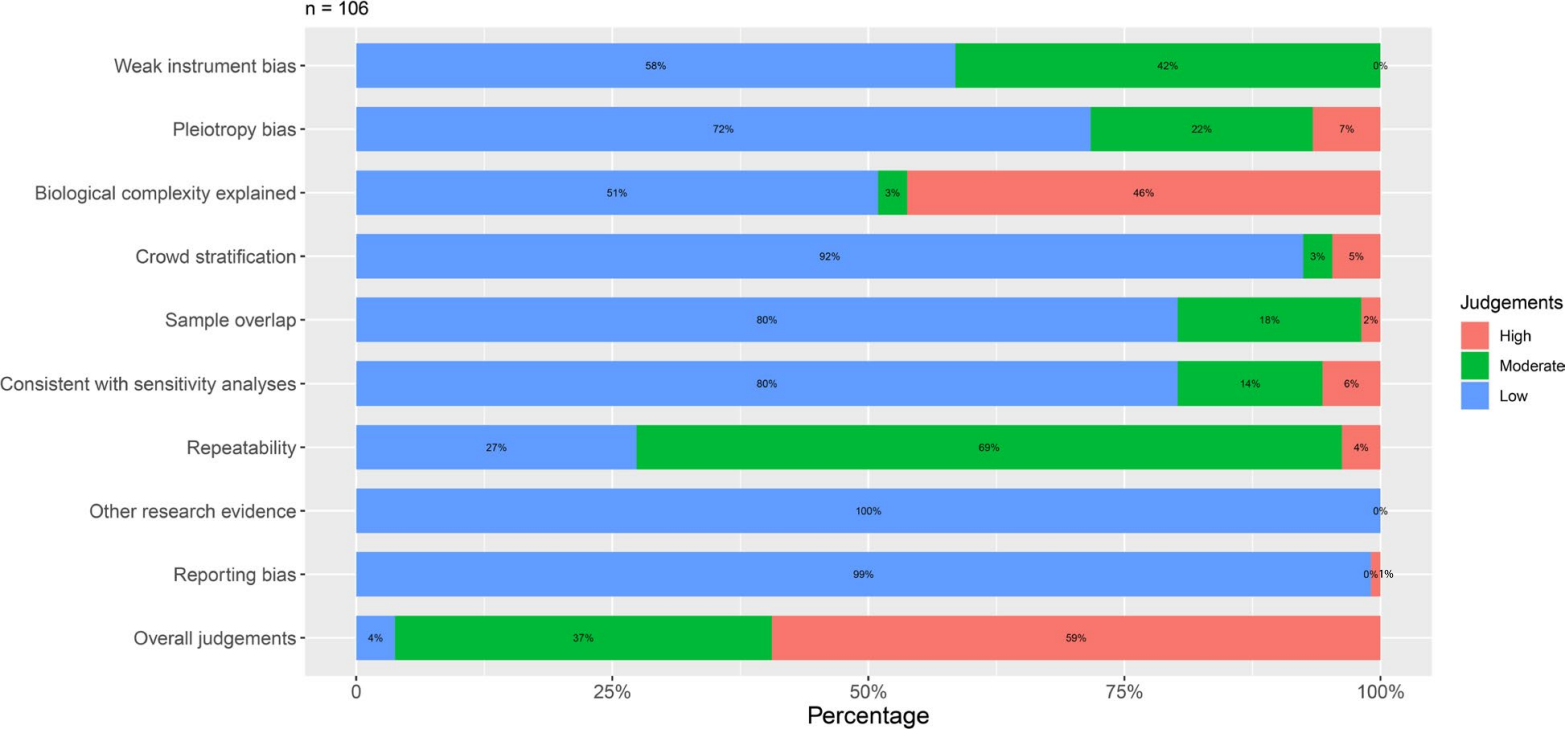
Risk of bias assessment in included AD-related MR studies. AD, Alzheimer’s dementia; MR, Mendelian randomization

**Fig. 4:**
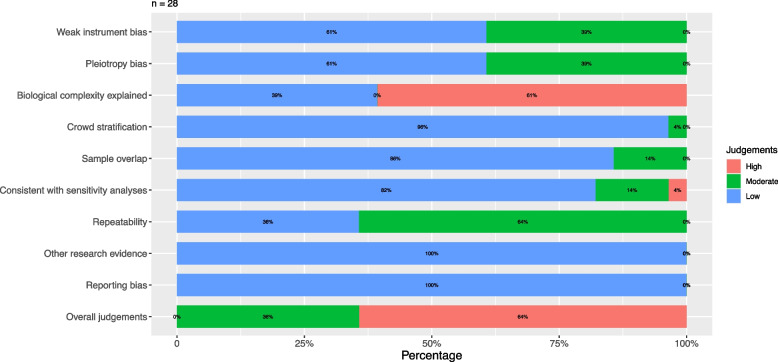
Risk of bias assessment in included cognition-related MR studies. MR, Mendelian randomization

One recognized advantage of MR studies is their ability to control for unmeasured bias, enhancing the validity of the results. However, MR studies face other biases. The risk of bias in the 106 AD-related MR studies and 28 cognition-related MR studies was assessed across three domains: bias of IV selection, bias of population selection, and bias in the selection of the reported result (Tables S7 and S8). Specifically, 63 (59%), 39 (37%), and 4 (4%) of the included articles on AD were rated as having high, moderate, and low risk of bias, respectively, based on overall judgment (Fig. 3). Similarly, 18 (64%) and 10 (36%) of the included articles on cognitive status were rated as having high and moderate risk of bias, respectively, according to predefined methodological criteria (Fig. 4). For MR studies of AD, the main high risk of bias comes from inappropriate biological evaluation of the included IVs (46%), pleiotropy bias (7%), crowd stratification (6%), and limited evidence from sensitivity analyses (6%). The main moderate risk of bias comes from limited repeatability evidence from other populations (69%), weak instrumental bias (42%), and pleiotropy bias (22%). Overall, biases such as limited repeatability evidence from other populations, inappropriate biological evaluation of the included IVs, weak instrumental bias, and pleiotropy bias faced a low proportion of low risk of bias (Fig. 3). For cognitive status, the main high risk of bias comes from inappropriate biological evaluation of the included IVs (61%) and limited evidence from sensitivity analyses (4%), and the main moderate risk of bias comes from limited repeatability evidence from other populations (64%), weak instrumental bias (39%), and pleiotropy bias (39%). Overall, biases such as limited repeatability evidence from other populations, inappropriate biological evaluation of the included IVs, weak instrumental bias, and pleiotropy bias, faced a low proportion of low risk of bias (Fig. 4). More detailed information is shown in Tables S7, S8 and Figs. 3 and 4.

## Discussion

To our knowledge, this study is the first systematic review of MR studies focusing on risk factors for AD and cognition, and assessing their quality. First, we systematically summarized and appraised modifiable risk factors for available interventions (including years of schooling, smoking, drinking) and omics signatures (including genome, proteome, DNA methylation, druggable targets) related to AD and cognition in MR studies. These findings may help implement preventive measures and accelerate the identification of relevant drugs for the prevention or treatment of AD and adverse cognitive performance. Second, we evaluated the risk of bias in MR studies, which may contribute to the development of a tool for assessing bias in MR study estimations.

First, this comprehensive systematic review of MR studies provided evidence supporting causality between various genetically determined modifiable factors/omics traits and AD. Specifically, four sociodemographic factors, ten lifestyle attributes, seven anthropometrics, 21 predispositions to diseases/phenotypes, eight dietary intakes and nutrients, 17 biochemical indices, eight inflammatory cytokines and biomarkers of inflammation, and 110 omics traits were causally associated with increased/reduced risk of AD. Similarly, the studies provided evidence that genetically predicted factors were causally associated with cognitive status, including six lifestyle attributes, four anthropometric indicators, three predispositions to diseases/phenotypes, four biochemical indicators, serum protein (CD33), and six omics traits. These causal risk factors for AD and cognitive status serve as the basis for future research to explore comorbidities of AD/cognitive status with other diseases.

The partial MR findings were further supported by meta-analyses, revealing that genetically predicted RA, higher educational attainment/years of schooling, DBP, 25(OH)D, TSH, and Lp(a) were causally associated with reduced risks of AD. Conversely, genetically determined short leukocyte telomeres and higher levels of LDL-C were identified as causal risk factors for AD. Only one meta-analysis provided evidence for cognitive status, showing that anemia, assessed by MCH, has a causative effect on cognitive performance. The significant causal risk factors identified from MR studies and meta-analyses may play a crucial role in preventing cognitive impairment from progressing to AD and may contribute to understanding the progression from cognitive impairment to AD from an etiological perspective.

It is noteworthy that the same causal risk factors (i.e., higher TC) and protective factors (i.e., computer use, birth weight, SBP, DBP, CD33, and longer telomere length) were found to be associated with AD and cognitive status. The consistent causality observed between these risk factors and AD and cognitive performance suggests that these genetically predicted factors for AD and cognition are governed by shared genetic mechanisms. However, not all shared factors of cognition and dementia have consistent effects on them. For instance, genetically predicted schizophrenia was found to be a causal risk factor for AD [26] but a protective factor for cognitive resilience [41]. Additionally, relatively few causal risk factors overlap between AD and cognitive status, which may suggest that cognitive status is an independent risk factor for AD. Future studies should focus on dementia and cognitive decline.

The ultimate goal of MR studies is to translate these discoveries into clinical practice. In this study, we introduced the clinical use of genetic findings in the field of AD and related cognitive status, identifying individuals at unusually high or low risk and enhancing the global knowledge of the biology of genetic variants associated with AD and cognitive status.

First, the MR approach can establish links between modifiable risk factors and AD as well as related cognitive status. MR occupies a unique position in the hierarchy of clinical evidence. The findings from MR studies, which are less prone to confounding and reverse causality bias, sit at the interface between traditional observational epidemiology (i.e., cohort studies) and interventional trials in terms of the level of evidence [16]. Our comprehensive review will help investigators judge the relative priority of modifiable factors for AD and related cognitive status across the lifespan for future research and clinical management of the disease. In particular, we found negative factors related to AD (including short sleep duration, CAD, schizophrenia, elevated LDL-C and CD33 protein levels, short telomeres, and high expression of *CR1*), which are crucial for setting inclusion and exclusion criteria in future population studies. For negative correlation factors, also known as competing events, subjects with these factors should be excluded to avoid false negatives or false positives in the observed associations.

Second, these discoveries could help generate hypotheses for drug repurposing. Drug repurposing provides a rapid approach to meet the urgent need for therapeutics to address AD and related cognitive status. The lower success rates of new therapeutics contribute to increased expenses, limiting the availability of effective medicines and leading to higher healthcare costs. Notably, only one in ten targets that entered clinical trials successfully received approval, with many demonstrating efficacy shortcomings (~ 50%) or adverse safety profiles (~ 25%) during late-stage clinical trials after many years of development [42, 43]. MR studies can assess whether molecular biomarkers are pathogenic, thereby saving time and money and preventing unnecessary adverse drug effects. It is recommended that MR combined with RCT research should be conducted for drug-targeted therapy to leverage the complementary advantages of both methods, transforming MR research results and improving RCT efficiency. Despite the modest impact of common variants identified through GWASs, the effect size of genetic variants on molecular phenotypes can be substantial. Furthermore, the effect of drugs on targets could also be amplified [44]. MR analysis derived from genetic instruments based on transcriptomic and proteomic data could identify therapeutic targets relevant to AD and related cognitive status.

The second aim of our study was to assess the quality of MR studies on AD and related cognitive performance. MR studies may be susceptible to high bias risk due to shortcomings in study design, methods, conduct, and analysis, potentially resulting in distorted estimates of causal associations between exposure and outcome. In this systematic review, the risk of bias in MR studies was assessed using nine items across three domains. More than half of the MR studies in the field of AD and related cognitive performance were found to be of poor methodological quality, with a high risk of bias accounting for 59% of the AD-related studies and 64% of the cognitive-related studies. Overall, the detected bias in AD/cognition-related MR studies was mainly caused by inappropriate biological evaluation of the included IVs, and the limited evidence from sensitivity analyses, the limited repeatability evidence from other populations, and weak instrumental bias and pleiotropy bias also contributed largely to the overall risk of bias in AD-related MR studies. The selection of IVs is crucial. While many studies emphasize eliminating possible pleiotropic bias, they seldom explain the biological mechanisms. More seriously, the number of SNPs reported in some studies was inconsistent with the number actually provided [45, 46], highlighting the need for improved reporting standardization of IVs in future studies.

The MR method is currently in the development stage, with many new sensitivity analysis methods emerging to improve result reliability [46, 47]. However, more methods can lead to inconsistent results and challenging interpretations. Conducting MR studies in diverse ethnic populations would be valuable for assessing the external validity of existing studies, which predominantly focus on European populations. Many experts agree that the evidence level of MR falls between that of observational studies and that of RCTs. The findings from MR studies should be interpreted within the context of existing evidence from observational studies and RCTs. When evidence from RCTs is not feasible, the findings from observational studies and MR studies in the same dataset should be compared. Future research in AD and related cognitive performance needs to consider the aforementioned biases (i.e., inappropriate biological evaluation of the included IVs, weak IV bias) to enhance the credibility of the findings.

In the past decade, significant progress has been made in developing tools to assess the validity of studies [23, 48, 49]. However, tools available for evaluating the risk of bias in estimates of MR studies remain limited. Our findings may pave the way for the development of a tool to assess the risk of bias in MR studies.

Our systematic review, pooling evidence from multiple sources, provides a comprehensive synopsis of the range and validity of reported associations between various factors and the risk of AD and related cognitive status. For the same exposure, if there are different GWAS data sources, we will perform a meta-analysis to summarize the results. To our knowledge, we performed the largest and most comprehensive assessment of MR studies for AD and related cognitive status risk thus far. We have created a database of MR studies on AD and related cognitive performance to avoid redundancy in future research. Additionally, our study evaluated the risk of bias in MR studies within this field to raise awareness and improve study quality.

There are notable limitations to consider. Summary GWAS data are crucial for MR studies. However, few MR studies have disclosed detailed information on GWASs or evaluated its quality. Although the Strengthening the Reporting of Genetic Association (STREGA) studies guidelines assess the strengths and weaknesses of this evidence [50], tools for evaluating the risk of bias in estimates from GWASs are still limited. Future MR studies may need to evaluate the quality of GWASs. As mentioned, the success of GWAS and MR studies has primarily focused on Caucasian populations, leading to imbalances in the translatability of findings. Genetic associations can differ across ethnicities, so future MR studies should investigate risk factors for AD and related cognitive status in diverse ancestral populations. Additionally, the factors investigated might represent those that were best understood rather than those most relevant to AD and related cognitive status. Cognitive impairment, a preclinical symptom of AD, has received far less attention than AD, necessitating more research in this area. Furthermore, although MR methods have several advantages over traditional meta-analysis methods and can provide evidence for associations of factors with AD and related cognitive status, they are still dependent on modeling experiments and assumptions. In addition, MR analysis is based on numerous assumptions that cannot be directly tested and may bias the results. Finally, we have only preliminarily explored the possible biases in MR studies; more biases will likely be exposed as methods develop, requiring ongoing attention.

## Conclusion

In this systematic review, we aimed to explain potentially paradoxical and implausible findings from MR analyses for AD and related cognitive status, highlighting the need for careful application and critical appraisal of MR findings. We provided a database of MR studies on AD and related cognitive performance to avoid redundancy in subsequent studies. Additionally, a critical appraisal checklist for assessing the risk of bias in MR studies may pave the way for the development of a standardized tool for evaluating bias in MR studies.

## Supplementary Information

Additional file 1. Table S1. Keywords and search strategy used in the system review.

Additional file 2. Table S2. Detailed description of the risk of bias assessment in the identified MR studies.

Additional file 3. Table S3. Study characteristics of included MR studies for AD.

Additional file 4. Table S4. The association between AD and risk factors.

Additional file 5. Table S5. Study characteristics of included MR studies for cognition.

Additional file 6. Table S6. The association between cognitive status and risk factors.

Additional file 7. Table S7. Risk of bias assessment for the included MR studies of AD.

Additional file 8. Table S8. Risk of bias assessment for the included MR studies of cognitive status.

Additional file 9. PRISMA_2020_checklist.

## Data Availability

All data generated or analyzed during this study are included in this published article and its supplementary information files.

## Abbreviations

AD: Alzheimer’s disease
ADGC: Alzheimer’s Disease Genetics Consortium
ADSP: Alzheimer’s Disease Sequencing Project
BMI: Body mass index
CAD: Coronary artery disease
CHARGE: Cohorts for Heart and Aging Research in Genomic Epidemiology consortium
CI: Confidence intervals
COGENT: Cognitive Genomics Consortium
CRP: C-reactive protein
DBP: Diastolic blood pressure
DSM-IV: Statistical Manual of Mental Disorders, 4th Edition
EADI: European Alzheimer’s disease Initiative
FEV1: Forced expiratory volume in 1 s
GERAD: Genetic and Environmental Risk in Alzheimer’s disease consortium
GWAS: Genome-wide association study
HDL: High-density lipoprotein
HDL-C: HDL-cholesterol
IGAP: International Genomics of Alzheimer’s Project
IL: Interleukin
Lp(a): Lipoprotein(a)
IV: Instrumental variable
IVW: Inverse-variance weighted
LDL-C: Low-density lipoprotein cholesterol
MCH: Mean corpuscular hemoglobin
mQTLs: Methylation quantitative trait loci
MR: Mendelian randomization
NINCDS-ADRDA: National Institute of Neurological and Communicative Disorders and Stroke and the Alzheimer’s Disease and Related Disorders Association
OR: Odds ratio
PERADES: Defining Genetic, Polygenic and Environmental Risk for Alzheimer’s Disease Consortium
PGC-ALZ: Alzheimer’s disease working group of the Psychiatric Genomics Consortium
PWAS: Proteome-wide association study
QTLs: Quantitative trait loci
RA: Rheumatoid arthritis
RCT: Randomized controlled trial
SBP: Systolic blood pressure
SD: Standard deviation
SMR: Summary data-based MR
SNP: Single-nucleotide polymorphism
SSGAC: Social Science Genetic Association Consortium
T2D: Type 2 diabetes
TC: Total cholesterol
TSH: Thyrotropin
UKB: UK Biobank
WC: Waist circumference
25(OH)D: 25-Hydroxyvitamin D
