# Supplementary material for: Summarizing attributable factors and evaluating risk of bias of Mendelian randomization studies for Alzheimer’s dementia and cognitive status: a systematic review and meta-analysis
Source: Syst Rev. 2025 Mar 13;14:61. doi: 10.1186/s13643-025-02792-5 (PMC11905674; doi:10.1186/s13643-025-02792-5)
Supplement: Supplementary file 1 — Additional file 1. Table S1. Keywords and search strategy used in the system review. [file 13643_2025_2792_MOESM1_ESM.docx]

# Table S1 Keywords and search strategy used in the system review

| Step | Terms |
| --- | --- |
| 1 | (("cognitive"[Title/Abstract] OR "cognition"[Title/Abstract] OR ("Alzheimer*"[Title/Abstract])) |
| 2 | ("genetic instrumental"[Title/Abstract] OR "genetic instrument"[Title/Abstract] OR "Mendelian randomization"[Title/Abstract] OR "Mendelian randomisation"[Title/Abstract] OR "instrumental variable"[Title/Abstract])) |
| 3 | #1 AND #2 AND (humans [Filter]) |
