# Supplementary material for: Summarizing attributable factors and evaluating risk of bias of Mendelian randomization studies for Alzheimer’s dementia and cognitive status: a systematic review and meta-analysis
Source: Syst Rev. 2025 Mar 13;14:61. doi: 10.1186/s13643-025-02792-5 (PMC11905674; doi:10.1186/s13643-025-02792-5)
Supplement: Supplementary file 2 — Additional file 2. Table S2. Detailed description of the risk of bias assessment in the identified MR studies. [file 13643_2025_2792_MOESM2_ESM.docx]

Table S2 Detailed description of the risk of bias assessment in the identified MR studies

| **Domains** | **Items** | **Explanation and signaling questions** | **Judgements** | | |
| --- | --- | --- | --- | --- | --- |
|  |  |  | **High risk of bias** | **Moderate risk of bias** | **Low risk of bias** |
| 1. Bias of instrumental variables selection | (1) Weak instrument bias | If genetic variants used as instrumental variables are weakly associated with the exposure of interest, the MR estimates can be biased. A partial F-statistic is commonly used as an indicator of potential weak instrument bias (when F is < 10 in an analysis). Were the Methods/Results section describing the evaluation of weak instrumental variables, that is, F statistics were calculated for instrumental variables, and the corresponding results are reported in the Results section? | The study reports the results for the F-statistic, but the F-statistic is less than 10. | Insufficient information is reported to make a judgement about risk of bias. | The study reports the results for the F-statistic, and the F-statistic is more than 10. |
|  | (2) Pleiotropy bias | The selected genetic variants should be independent of multiple factors (vertical pleiotropy) or biological pathways (horizontal pleiotropy) of exposure-outcome association, and the genetic variants should be independent of the outcomes except via exposure; otherwise, this event is a violation of the exclusion restriction assumption and a source of bias in MR studies. Was horizontal pleiotropy evaluated in the risk of bias assessment? The MR-Egger regression analysis was performed to evaluate pleiotropy based on the intercept. | The study reports the threshold of the MR-Egger intercept was less than 0.05. | Insufficient information is reported to make a judgement about risk of bias. | The study reports the threshold of the MR-Egger intercept was more than 0.05. |
|  | (3) Biological complexity explained | The biological mechanism of genetic variation is the basis of strong instrumental variables and reduced pleiotropy. Were there biological explanations for instrumental variables, including the process for screening IVs in the method section and/or incorporated IVs in the analysis being explained in the discussion? | IVs were screened only by *P* value in the study. | There is insufficient information reported to inform a judgement on risk bias. | The detailed screening process and basis of IVs were introduced in the methods section, and the biological effects of IVs were properly explained in the introduction, methods or discussion sections. |
| 2. Bias of population selection | (1) Crowd stratification | It means that the frequency of genetic variation is different among people with different genetic backgrounds, resulting in false association between genetic variation and outcome. In MR study, population stratification can lead to the failure of independence hypothesis or exclusivity hypothesis, and then lead to wrong causal inference. Was the genetic background of the study population taken from the same for exposure and outcome? | The genetic background of the GWAS database corresponding to exposure and outcome was different. | Insufficient information is reported to make a judgement about crowd stratification. | The GWAS corresponding to exposure and outcome had the same genetic background, and there was no problem of population stratification. |
|  | (2) Sample overlap | The estimate from a two-sample analysis, in which data on the exposure and outcome are taken from non-overlapping datasets, is less biased. Was the genetic background of the study population taken from non-overlapping datasets for exposure and outcome? | There was the problem of database overlap between exposure and outcome, and sensitivity analyses found the bias of sample overlap. | Insufficient information is reported to make a judgement about sample overlap. | There was no problem of sample overlap, or there was the problem of sample overlap and the analysis could figure out the bias of sample overlap or the sensitivity analysis provided stable results or the problem of sample overlap did not bias the conclusion. |
| 3. Bias in selection of the reported result | (1) Consistent with sensitivity analyses | Many methods derived as sensitivity analysis can increase the reliability of the results. In detailed, were the results of sensitivity analyses (such as MR-Egger, weighted mode-based estimator, simple median, weighted median, weighted median of penalty, and/or MR-PRESSO, etc.) reported in the study, and were they consistent with the main results of MR analyses (IVW results)? | This study reported the results of sensitivity analysis, which were completely inconsistent with the IVW results. Or the results of sensitivity analyses were reported in the study, which were partially (< 50%) consistent with IVW result. | The results of the sensitivity analysis were not reported. | The results of sensitivity analyses were reported in the study, which were partially (≥ 50%) or completely (100%) consistent with IVW result. |
|  | (2) Repeatability (Winner's curse) | The stability of the results of external verification by different populations and ethnic groups. Were there data from other databases to validate the results? | Validation with data from other populations/sources is reported in the findings, but the results were not completely (< 50%) replicated in the validation population. | Results for validation samples were not found in the study. | Validation of the results with data from other populations/sources is reported in the findings. And the results were partially (≥ 50%) or completely (100%) validated in the replication study. |
|  | (3) Other research evidence | Interpretation of MR findings should be based on comprehensive evidence. The findings from Mendelian randomization study should be interpreted in the context of existing evidence from observational study and/or RCT. | The study did not compare or synthesize evidence from other types of studies. | NA | Evidence from RCT and/or observational studies the association between exposures and outcome is presented in the introduction and/or discussion section. |
|  | (4) Reporting bias | Was the content and purpose of the study consistent? Selective reporting of results in a way that depends on the findings and prevents the estimate from being included in a Mendelian randomization analysis. Specifically, whether the associations between target exposures and outcomes intended to be analyzed in the study are both reported in the results section. | Associations between partial exposures and outcomes were selectively reported in the results section, deliberately emphasizing results of statistical significance. | There is insufficient information reported to inform a judgement on reporting bias. | The content of the research carried out was consistent with the previously set research objectives. Associations between target exposure and outcomes intended to be analyzed in the study are reported in the results section. |
| **Overall judgement-Risk of bias in the MR** | Overall judgement-Risk of bias in the MR | The evidence level of Mendelian randomization studies is evaluated based on the above 9 domains. | Within each domain: the study has some important problems in this domain; Across domains: the study has some important problems; **Criterion:** the study is judged to be at high risk of bias in at least one domain. | Within each domain: there is insufficient information reported to make a judgement on risk of bias; Across domains: the study provides insufficient evidence for a MR; **Criterion:** the study is judged to be at moderate risk of bias for all domains or at moderate (at least one domain) and low risk of bias for all domains. | Within each domain: the study is a well performed MR with regard to this domain; Across domains: the study is comparable to a well performed randomised trial; **Criterion:** the study is judged to be at low risk of bias for all domains. The findings of the meta-analysis are likely to be reliable, domain 1-9 did not raise any concerns with the review process or concerns were appropriately considered in the review conclusions, the conclusions were supported by the evidence and included consideration of the relevance of included studies. |

GWAS, genome wide association study; IVs, instrumental variables; IVW, inverse-variance weighted; MR, Mendelian randomization; RCT, randomized controlled trial
