# Supplementary material for: Summarizing attributable factors and evaluating risk of bias of Mendelian randomization studies for Alzheimer’s dementia and cognitive status: a systematic review and meta-analysis
Source: Syst Rev. 2025 Mar 13;14:61. doi: 10.1186/s13643-025-02792-5 (PMC11905674; doi:10.1186/s13643-025-02792-5)
Supplement: Supplementary file 4 — Additional file 4. Table S4. The association between AD and risk factors. [file 13643_2025_2792_MOESM4_ESM.docx]

**Table S4 The association between AD and risk factors.**

| Publication | Risk of factor (exposure) | Odds ratio (95% confidence interval) | *P* | Meta-analysis_Cochran's Q | Meta-analysis_df | Meta-analysis_I2 |
| --- | --- | --- | --- | --- | --- | --- |
| **Sociodemographic factors** |  |  |  |  |  |  |
| Meta-analysis | Educational attainment/Years of schooling | 0.755 (0.658-0.866) | 0.001 | 48.638 | 10 | 0.794 |
| Wang, Z.-2020 [1] | Years of schooling | 0.590 (0.450-0.770) | <0.001 |  |  |  |
| Zhang, Q.-2020 [2] | Years of schooling | 0.674 (0.571-0.796) | <0.001 |  |  |  |
| Østergaard, S. D.-2015 [3] | Years of education | 0.710 (0.480-1.060) | 0.097 |  |  |  |
| Larsson, S. C.-2017 [4] | Years of education | 0.890 (0.840-0.930) | <0.001 |  |  |  |
| Andrews, S. J.-2021 [5] | Educational attainment | 0.680 (0.600-0.780) | <0.001 |  |  |  |
| Anderson, E. L.-2020 [6] | Educational attainment/Years of schooling | 1.150 (0.680-1.930) | 0.600 |  |  |  |
| Wang, H.-2021 [7] | Educational attainment/Years of schooling (male) | 1.085 (0.617-1.909) | 0.775 |  |  |  |
| Wang, H.-2021 [7] | Educational attainment/Years of schooling (female) | 0.571 (0.366-0.891) | 0.013 |  |  |  |
| Ko, H.-2021 [8] | Educational attainment/Years of schooling | 1.080 (0.620-1.910) | 0.780 |  |  |  |
| Jansen, I. E.-2019 [9] | Educational attainment/Years of schooling | 0.875 (0.814-0.941) | <0.001 |  |  |  |
| Raghavan, N. S.-2019 [10] | Educational attainment/Years of schooling | 0.630 (0.540-0.740) | <0.001 |  |  |  |
| Meta-analysis | College/University completion | 0.798 (0.186-3.416) | 0.298 | 1.661 | 1 | 0.398 |
| Larsson, S. C.-2017 [4] | College/university | 0.740 (0.630-0.860) | <0.001 |  |  |  |
| Østergaard, S. D.-2015 [3] | University completion | 0.950 (0.670-1.340) | 0.752 |  |  |  |
| Meta-analysis | Intelligence | 0.633 (0.491-0.816) | 0.011 | 2.654 | 3 | 0.000 |
| Anderson, E. L.-2020 [6] | Intelligence | 0.620 (0.440-0.880) | 0.008 |  |  |  |
| Wang, H.-2021 [7] | Intelligence (male) | 0.632 (0.443-0.901) | 0.011 |  |  |  |
| Wang, H.-2021 [7] | Intelligence (female) | 0.537 (0.406-0.709) | <0.001 |  |  |  |
| Larsson, S. C.-2017 [4] | Intelligence | 0.730 (0.570-0.930) | 0.010 |  |  |  |
| Li, M.-2021 [11] | Age at menarche | 0.903 (0.807-1.010) | 0.075 |  |  |  |
| Li, M.-2021 [11] | Age at menopause | 0.991 (0.957-1.026) | 0.611 |  |  |  |
| Ko, H.-2021 [8] | Occupational attainment | 0.720 (0.540-0.950) | 0.020 |  |  |  |
| Jansen, I. E.-2019 [9] | Childhood cognitive ability | 0.983 (0.945-1.022) | 0.400 |  |  |  |
| Jansen, I. E.-2019 [9] | Age at First Birth | 1.026 (0.844-1.249) | 0.791 |  |  |  |
| **Lifestyle attributes** |  |  |  |  |  |  |
| Meta-analysis | Chronotype | 0.995 (0.968-1.024) | 0.538 | 0.151 | 2 | 0.000 |
| Huang, J.-2020 [12] | Chronotype | 0.992 (0.912-1.079) | 0.851 |  |  |  |
| Huang, J.-2020 [12] | Chronotype | 0.995 (0.982-1.008) | 0.441 |  |  |  |
| Anderson, E. L.-2021 [13] | Chronotype | 1.020 (0.900-1.160) | 0.770 |  |  |  |
| Meta-analysis | Long sleep duration | 1.049 (0.619-1.779) | 0.733 | 0.611 | 2 | 0.000 |
| Huang, J.-2020 [12] | Long sleep duration | 2.678 (0.147-48.96) | 0.506 |  |  |  |
| Huang, J.-2020 [12] | Long sleep duration | 0.983 (0.693-1.394) | 0.922 |  |  |  |
| Anderson, E. L.-2021 [13] | Long sleep duration | 1.100 (0.790-1.540) | 0.570 |  |  |  |
| Meta-analysis | Short sleep duration | 1.213 (0.917-1.606) | 0.097 | 0.297 | 2 | 0.000 |
| Huang, J.-2020 [12] | Short sleep duration | 1.560 (0.608-3.999) | 0.355 |  |  |  |
| Huang, J.-2020 [12] | Short sleep duration | 1.214 (1.044-1.411) | 0.012 |  |  |  |
| Anderson, E. L.-2021 [13] | Short sleep duration | 1.190 (0.930-1.530) | 0.160 |  |  |  |
| Meta-analysis | Sleep duration | 0.993 (0.960-1.027) | 0.629 | 3.246 | 6 | 0.000 |
| Huang, J.-2020 [12] | Self-reported sleep duration | 0.943 (0.803-1.190) | 0.622 |  |  |  |
| Huang, J.-2020 [12] | Self-reported sleep duration | 0.999 (0.966-1.033) | 0.938 |  |  |  |
| Andrews, S. J.-2021 [5] | Sleep duration | 1.050 (0.890-1.240) | 0.570 |  |  |  |
| Henry, A.-2019 [14] | Sleep duration | 0.890 (0.670–1.180) | 0.410 |  |  |  |
| Huang, J.-2020 [12] | Accelerometer-based sleep duration | 0.962 (0.731-1.266) | 0.782 |  |  |  |
| Huang, J.-2020 [12] | Accelerometer-based sleep duration | 0.985 (0.934-1.039) | 0.572 |  |  |  |
| Anderson, E. L.-2021 [13] | Accelerometer-sleep duration | 0.740 (0.480-1.160) | 0.190 |  |  |  |
| Meta-analysis | L5Timing | 0.970 (0.876- 1.076) | 0.336 | 1.074 | 2 | 0.000 |
| Huang, J.-2020 [12] | L5Timing | 1.063 (0.704-1.603) | 0.773 |  |  |  |
| Huang, J.-2020 [12] | L5Timing | 0.971 (0.927-1.019) | 0.234 |  |  |  |
| Anderson, E. L.-2021 [13] | L5Timing | 0.730 (0.400-1.310) | 0.290 |  |  |  |
| Meta-analysis | Number of sleep episodes | 0.896 (0.202-3.973) | 0.520 | 3.844 | 1 | 0.740 |
| Huang, J.-2020 [12] | Number of sleep episodes | 0.770 (0.606-0.979) | 0.033 |  |  |  |
| Huang, J.-2020 [12] | Number of sleep episodes | 0.981 (0.949-1.014) | 0.252 |  |  |  |
| Meta-analysis | Insomnia | 1.031 (0.959-1.108) | 0.306 | 0.680 | 4 | 0.000 |
| Andrews, S. J.-2021 [5] | Insomnia symptoms | 0.910 (0.650-1.270) | 0.570 |  |  |  |
| Huang, J.-2020 [12] | Insomnia | 0.994 (0.638-1.550) | 0.980 |  |  |  |
| Huang, J.-2020 [12] | Insomnia | 1.029 (0.965-1.099) | 0.377 |  |  |  |
| Anderson, E. L.-2021 [13] | Frequent insomnia | 1.000 (0.590-1.680) | 0.990 |  |  |  |
| Jansen, I. E.-2019 [9] | Insomnia | 1.046 (0.958-1.142) | 0.315 |  |  |  |
| Meta-analysis | Sleep efficiency | 0.996 (0.769-1.291) | 0.955 | 5.565 | 2 | 0.641 |
| Huang, J.-2020 [12] | Sleep efficiency | 1.281 (0.799-2.056) | 0.302 |  |  |  |
| Huang, J.-2020 [12] | Sleep efficiency | 0.982 (0.923-1.044) | 0.562 |  |  |  |
| Cullell, N.-2021 [15] | Sleep efficiency | 2.67E-05 (1.40E-09-0.511) | 0.036 |  |  |  |
| Anderson, E. L.-2021 [13] | Daytime napping | 0.670 (0.450-1.020) | 0.060 |  |  |  |
| Anderson, E. L.-2021 [13] | Daytime sleepiness | 0.650 (0.290-1.430) | 0.280 |  |  |  |
| Anderson, E. L.-2021 [13] | Sleep fragmentation | 1.120 (0.870-1.450) | 0.370 |  |  |  |
| Meta-analysis | Alcohol consumption | 0.922 (0.572-1.486) | 0.540 | 2.869 | 2 | 0.303 |
| Andrews, S. J.-2021 [5] | Alcohol Consumption | 1.130 (0.760-1.670) | 0.550 |  |  |  |
| Andrews, S. J.-2020 [16] | Alcohol consumption | 0.960 (0.740-1.250) | 0.775 |  |  |  |
| Larsson, S. C.-2017 [4] | Alcohol consumption | 0.720 (0.500-1.040) | 0.080 |  |  |  |
| Meta-analysis | AUDIT scores | 1.451 (0.850-2.477) | 0.096 | 3.068 | 2 | 0.348 |
| Andrews, S. J.-2021 [5] | AUDIT scores | 1.480 (1.060-2.060) | 0.026 |  |  |  |
| Andrews, S. J.-2021 [5] | AUDIT scores | 1.550 (1.070-2.250) | 0.023 |  |  |  |
| Andrews, S. J.-2020 [16] | AUDIT scores | 0.450 (0.120-1.750) | 0.250 |  |  |  |
| Andrews, S. J.-2020 [16] | Alcohol dependence | 0.980 (0.930-1.040) | 0.588 |  |  |  |
| Yang, Y. X.-2020 [17] | Drinks per week | 0.990 (0.780-1.260) | 0.940 |  |  |  |
| Meta-analysis | Smoking initiation | 0.942 (0.222-4.006) | 0.694 | 0.856 | 1 | 0.000 |
| Andrews, S. J.-2021 [5] | Smoking initiation | 0.980 (0.770-1.240) | 0.840 |  |  |  |
| Larsson, S. C.-2017 [4] | Smoking initiation | 0.710 (0.370-1.330) | 0.280 |  |  |  |
| Meta-analysis | Smoking quantity: cigarettes per day | 0.777 (0.453-1.334) | 0.183 | 4.599 | 2 | 0.565 |
| Larsson, S. C.-2017 [4] | Smoking quantity: cigarettes per day | 0.690 (0.490-0.990) | 0.040 |  |  |  |
| Østergaard, S. D.-2015 [3] | Smoking quantity: cigarettes per day | 0.670 (0.510-0.890) | 0.007 |  |  |  |
| Andrews, S. J.-2021 [5] | Smoking quantity: cigarettes per day | 0.960 (0.760-1.210) | 0.730 |  |  |  |
| Meta-analysis | Smoking cessation | 0.995 (0.887-1.116) | 0.876 | 2.045 | 2 | 0.022 |
| Yang, Y. X.-2020 [17] | Smoking cessation | 0.900 (0.760-1.060) | 0.200 |  |  |  |
| Larsson, S. C.-2017 [4] | Smoking cessation | 1.160 (0.750-1.780) | 0.520 |  |  |  |
| Jansen, I. E.-2019 [9] | Ever Smoked | 1.003 (0.980-1.027) | 0.771 |  |  |  |
| Zhang, Q.-2020 [2] | Current tobacco smoking | 0.737 (0.321-1.692) | 0.472 |  |  |  |
| Meta-analysis | Subjective well-being | 0.942 (0.797-1.112) | 0.262 | 0.196 | 2 | 0.000 |
| Wang, H.-2021 [7] | Subjective well-being (male) | 0.845 (0.300-2.379) | 0.751 |  |  |  |
| Wang, H.-2021 [7] | Subjective well-being (female) | 1.108 (0.491-2.500) | 0.803 |  |  |  |
| Jansen, I. E.-2019 [9] | Subjective well-being | 0.941 (0.872-1.016) | 0.123 |  |  |  |
| Meta-analysis | Neuroticism | 0.990 (0.892-1.099) | 0.447 | 0.022 | 1 | 0.000 |
| Jansen, I. E.-2019 [9] | Neuroticism | 0.995 (0.933-1.061) | 0.879 |  |  |  |
| Ma, Y. H.-2021 [18] | Neuroticism | 0.990 (0.974-1.007) | 0.262 |  |  |  |
| Meta-analysis | Regular participation in pub/social club | 1.170 (0.353-3.878) | 0.344 | 0.350 | 1 | 0.000 |
| Shen, L. X.-2021 [19] | Regular participation in pub/social club | 1.190 (0.981-1.443) | 0.077 |  |  |  |
| Shen, L. X.-2021 [19] | Regular participation in pub/social club | 0.972 (0.511-1.847) | 0.931 |  |  |  |
| Meta-analysis | Regular participation in sports club/gym | 0.381 (0.000-2863.900) | 0.401 | 4.016 | 1 | 0.751 |
| Shen, L. X.-2021 [19] | Regular participation in sports club/gym | 0.670 (0.463-0.970) | 0.034 |  |  |  |
| Shen, L. X.-2021 [19] | Regular participation in sports club/gym | 0.159 (0.041-0.619) | 0.008 |  |  |  |
| Meta-analysis | Regular participation in religious group | 0.841 (0.201-3.520) | 0.366 | 0.330 | 1 | 0.000 |
| Shen, L. X.-2021 [19] | Regular participation in religious group | 0.852 (0.680-1.068) | 0.165 |  |  |  |
| Shen, L. X.-2021 [19] | Regular participation in religious group | 0.617 (0.210-1.814) | 0.380 |  |  |  |
| Meta-analysis | Loneliness | 0.978 (0.814-1.176) | 0.731 | 0.304 | 3 | 0.000 |
| Shen, L. X.-2021 [19] | Loneliness | 0.900 (0.629-1.289) | 0.566 |  |  |  |
| Shen, L. X.-2021 [19] | Loneliness | 0.890 (0.232-3.415) | 0.865 |  |  |  |
| Shen, L. X.-2021 [19] | Loneliness (MTAG) | 0.993 (0.874-1.127) | 0.910 |  |  |  |
| Shen, L. X.-2021 [19] | Loneliness (MTAG) | 0.950 (0.659-1.371) | 0.785 |  |  |  |
| Andrews, S. J.-2021 [5] | Social isolation | 0.960 (0.720-1.290) | 0.820 |  |  |  |
| Ma, Y. H.-2021 [18] | Agreeableness | 0.999 (0.996-1.002) | 0.477 |  |  |  |
| Ma, Y. H.-2021 [18] | Openness | 1.000 (0.998-1.003) | 0.738 |  |  |  |
| Ma, Y. H.-2021 [18] | Conscientiousness | 1.002 (0.999-1.005) | 0.270 |  |  |  |
| Ma, Y. H.-2021 [18] | Extraversion | 1.012 (0.991-1.003) | 0.262 |  |  |  |
| Yang, Y. X.-2020 [17] | Automobile speeding propensity | 0.820 (0.580-1.150) | 0.250 |  |  |  |
| Yang, Y. X.-2020 [17] | Number of sexual partners | 0.500 (0.270-0.930) | 0.040 |  |  |  |
| Yang, Y. X.-2020 [17] | Self-reported general risk tolerance | 1.160 (0.870-1.520) | 0.280 |  |  |  |
| Meta-analysis | Risk-taking tendency | 0.887 (0.263-2.994) | 0.428 | 4.794 | 1 | 0.791 |
| Yang, Y. X.-2020 [17] | Risk-taking tendency | 0.790 (0.670–0.940) | 0.007 |  |  |  |
| Yang, Y. X.-2020 [17] | Risk-taking tendency | 0.960 (0.910-0.990) | 0.040 |  |  |  |
| Meta-analysis | Vigorous physical activity | 1.037 (0.562-1.916) | 0.586 | 0.143 | 1 | 0.000 |
| Baumeister, S. E.-2020 [20] | Vigorous physical activity | 0.910 (0.460-1.810) | 0.794 |  |  |  |
| Baumeister, S. E.-2020 [20] | Vigorous physical activity | 1.040 (0.950-1.150) | 0.412 |  |  |  |
| Meta-analysis | Physical activity | 0.995 (0.941-1.052) | 0.731 | 1.283 | 2 | 0.000 |
| Wu, P. F.-2021 [21] | Physical activity: Accelerometer-based physical activity measurement-average acceleration | 1.030 (0.480-2.210) | 0.940 |  |  |  |
| Wu, P. F.-2021 [21] | Physical activity: Accelerometer-based physical activity measurement-average acceleration | 1.030 (0.960-1.100) | 0.320 |  |  |  |
| Baumeister, S. E.-2020 [20] | Physical activity | 0.990 (0.990-1.010) | 0.489 |  |  |  |
| Andrews, S. J.-2021 [5] | Moderate-to-vigorous physical activity | 1.320 (1.030-1.710) | 0.038 |  |  |  |
| Yang, F.-2021 [22] | Television watching | 1.150 (0.970-1.360) | 0.110 |  |  |  |
| Yang, F.-2021 [22] | Computer use | 0.670 (0.480-0.920) | 0.010 |  |  |  |
| Yang, F.-2021 [22] | Driving behavior | 1.260 (0.500-3.190) | 0.630 |  |  |  |
| **Anthropometrics** |  |  |  |  |  |  |
| Meta-analysis | WHR | 1.020 (0.900-1.158) | 0.296 | 0.031 | 1 | 0.000 |
| Li, X.-2021 [23] | WHR | 1.020 (1.000-1.040) | 0.112 |  |  |  |
| Zhou, Y.-2019 [24] | WHR | 1.050 (0.760-1.450) | 0.761 |  |  |  |
| Meta-analysis | WHRadjBMI | 1.154 (0.432-3.078) | 0.315 | 0.112 | 1 | 0.000 |
| Zhou, Y.-2019 [24] | WHRadjBMI | 1.120 (0.890-1.410) | 0.324 |  |  |  |
| Larsson, S. C.-2017 [4] | WHRadjBMI | 1.180 (0.970-1.450) | 0.100 |  |  |  |
| Meta-analysis | Pulse pressure | 0.991 (0.961-1.021) | 0.159 | 0.571 | 1 | 0.000 |
| Ou, Y. N.-2021 [25] | Pulse pressure | 0.995(0.983-1.007) | 0.435 |  |  |  |
| Andrews, S. J.-2021 [5] | Pulse pressure | 0.990 (0.990-1.000) | 0.096 |  |  |  |
| Meta-analysis | Height | 0.903 (0.428-1.905) | 0.334 | 21.533 | 1 | 0.954 |
| Zhu, Z.-2018 [26] | Height | 0.850 (0.810-0.890) | <0.001 |  |  |  |
| Jansen, I. E.-2019 [9] | Height | 0.956 (0.941-0.971) | <0.001 |  |  |  |
| Meta-analysis | Waist circumference | 1.030 (0.829-1.279) | 0.335 | 0.008 | 1 | 0.000 |
| Li, X.-2021 [23] | Waist circumference | 1.030 (1.000-1.070) | 0.048 |  |  |  |
| Zhou, Y.-2019 [24] | Waist circumference | 1.020 (0.820-1.270) | 0.836 |  |  |  |
| Meta-analysis | SBP | 0.958 (0.877-1.046) | 0.283 | 17.868 | 7 | 0.608 |
| Sproviero, W.-2021 [27] | SBP | 0.869 (0.773-0.978) | 0.030 |  |  |  |
| Wang, H.-2021 [7] | SBP (male) | 1.618 (0.824-3.175) | 0.162 |  |  |  |
| Wang, H.-2021 [7] | SBP (female) | 0.824 (0.485-1.402) | 0.476 |  |  |  |
| Ou, Y. N.-2021 [25] | SBP | 0.998 (0.991-1.005) | 0.567 |  |  |  |
| Andrews, S. J.-2021 [5] | SBP | 1.000 (0.990-1.000) | 0.140 |  |  |  |
| Walker, V. M.-2020 [28] | SBP | 1.040 (0.950-1.130) | 0.450 |  |  |  |
| Østergaard, S. D.-2015 [3] | SBP | 0.750 (0.620-0.910) | 0.003 |  |  |  |
| Larsson, S. C.-2017 [4] | SBP | 0.940 (0.770-1.140) | 0.510 |  |  |  |
| Meta-analysis | DBP | 0.989 (0.980-0.999) | 0.035 | 6.989 | 5 | 0.285 |
| Sproviero, W.-2021 [27] | DBP | 0.869 (0.773-0.978) | 0.030 |  |  |  |
| Wang, H.-2021 [7] | DBP (male) | 0.610 (0.300-1.241) | 0.172 |  |  |  |
| Wang, H.-2021 [7] | DBP (female) | 0.825 (0.473-1.440) | 0.500 |  |  |  |
| Ou, Y. N.-2021 [25] | DBP | 0.990 (0.979-1.000) | 0.055 |  |  |  |
| Andrews, S. J.-2021 [5] | DBP | 0.990 (0.982-0.998) | 0.016 |  |  |  |
| Larsson, S. C.-2017 [4] | DBP | 0.960 (0.790-1.160) | 0.650 |  |  |  |
| Meta-analysis | BMI | 0.994 (0.956-1.034) | 0.755 | 19.476 | 9 | 0.538 |
| Li, X.-2021 [23] | BMI | 1.030 (1.010-1.050) | 0.003 |  |  |  |
| Wang, H.-2021 [7] | BMI (male) | 1.439 (0.919-2.254) | 0.112 |  |  |  |
| Wang, H.-2021 [7] | BMI (female) | 0.927 (0.651-1.319) | 0.675 |  |  |  |
| Andrews, S. J.-2021 [5] | BMI | 0.980 (0.910-1.070) | 0.710 |  |  |  |
| Zhang, Q.-2020 [2] | BMI | 0.904 (0.815-1.002) | 0.055 |  |  |  |
| Mukherjee, S.-2015 [29] | BMI | 0.950 (0.910-1.000) | 0.058 |  |  |  |
| Mukherjee, S.-2015 [29] | BMI | 0.960 (0.870-1.070) | 0.488 |  |  |  |
| Larsson, S. C.-2017 [4] | BMI | 1.050 (0.910-1.210) | 0.510 |  |  |  |
| Nordestgaard, L. T.-2017 [30] | BMI | 1.020 (0.860-1.220) | 0.820 |  |  |  |
| Li, Q. S.-2020 [31] | BMI | 1.022 (0.995-1.050) | 0.111 |  |  |  |
| Li, Q. S.-2020 [31] | Extreme BMI | 1.007 (0.999-1.015) | 0.093 |  |  |  |
| Li, X.-2021 [23] | Childhood BMI | 1.010 (0.990-1.040) | 0.226 |  |  |  |
| Li, X.-2021 [23] | Body fat percentage | 1.000 (0.950-1.060) | 0.960 |  |  |  |
| Li, Q. S.-2020 [31] | Extreme height | 0.996 (0.992-0.999) | 0.015 |  |  |  |
| Li, X.-2021 [23] | Birth weight | 0.970 (0.940-1.000) | 0.038 |  |  |  |
| **Predisposition to diseases/phenotypes** |  |  |  |  |  |  |
| ***Diseases of the circulatory*** |  |  |  |  |  |  |
| Meta-analysis | CAD | 1.024 (0.932-1.126) | 0.387 | 4.361 | 2 | 0.541 |
| Li, Q. S.-2020 [31] | CAD | 1.034 (0.954-1.119) | 0.423 |  |  |  |
| Li, Q. S.-2020 [31] | CAD | 1.001 (0.986-1.015) | 0.940 |  |  |  |
| Grace, C.-2018 [32] | CAD | 1.070 (1.010-1.150) | 0.027 |  |  |  |
| Wang, T.-2020 [33] | Any stroke | 0.962 (0.758-1.221) | 0.750 |  |  |  |
| Wang, T.-2020 [33] | Any ischemic stroke | 0.902 (0.742-1.097) | 0.305 |  |  |  |
| Wang, T.-2020 [33] | Large-artery atherosclerotic stroke | 1.038 (0.914-1.178) | 0.568 |  |  |  |
| Wang, T.-2020 [33] | Cardio-embolic stroke | 1.066 (0.841-1.045) | 0.245 |  |  |  |
| Kwok, M. K.-2021 [34] | Atrial Fibrillation | 1.000 (1.000-1.010) | 0.370 |  |  |  |
| Li, Q. S.-2020 [31] | Non-cancer illness code self-reported: angina | 4.978 (0.302-81.941) | 0.262 |  |  |  |
| ***Diseases/phenotypes of the nervous system and mental symptom*** |  |  |  |  |  |  |
| Meta-analysis | Cognitive ability | 0.666 (0.285-1.557) | 0.176 | 11.566 | 2 | 0.827 |
| Wang, H.-2021 [7] | Cognitive performance (male) | 0.575 (0.353-0.936) | 0.026 |  |  |  |
| Wang, H.-2021 [7] | Cognitive performance (female) | 0.498 (0.338-0.732) | <0.001 |  |  |  |
| Jansen, I. E.-2019 [9] | Cognitive ability | 0.890 (0.850-0.920) | <0.001 |  |  |  |
| Meta-analysis | Multiple sclerosis | 1.004 (0.936-1.076) | 0.633 | 5.931 | 1 | 0.831 |
| Li, Q. S.-2020 [31] | Multiple sclerosis | 1.009 (1.003-1.015) | 0.005 |  |  |  |
| Li, Q. S.-2020 [31] | Multiple sclerosis | 0.998 (0.991-1.004) | 0.505 |  |  |  |
| Li, Q. S.-2020 [31] | Amyotrophic lateral sclerosis | 1.040 (0.931-1.162) | 0.493 |  |  |  |
| Han, Z.-2018 [35] | PD | 0.918 (0.782-1.076) | 0.291 |  |  |  |
| Han, Z.-2018 [35] | α-synuclein in PD | 0.638 (0.485-0.838) | 0.001 |  |  |  |
| Meta-analysis | Major depressive disorder | 1.009 (0.850-1.198) | 0.634 | 2.352 | 1 | 0.575 |
| Huang, J.- 2020 [12] | Major depressive disorder | 1.030 (0.992-1.069) | 0.124 |  |  |  |
| Huang, J.- 2020 [12] | Major depressive disorder | 1.000 (0.995-1.006) | 0.938 |  |  |  |
| Meta-analysis | Depression | 0.979 (0.567-1.691) | 0.712 | 2.601 | 1 | 0.616 |
| Andrews, S. J.-2021 [5] | Depression | 0.930 (0.850-1.020) | 0.130 |  |  |  |
| Jansen, I. E.-2019 [9] | Depressive Symptoms | 1.015 (0.961-1.072) | 0.599 |  |  |  |
| Jansen, I. E.-2019 [9] | Schizophrenia | 1.016 (1.004-1.028) | 0.012 |  |  |  |
| Jansen, I. E.-2019 [9] | Bipolar Disorder | 1.009 (0.999-1.019) | 0.061 |  |  |  |
| Daghlas, I.-2020 [36] | Migraine | 1.010 (1.000–1.020) | 0.070 |  |  |  |
| ***Diseases of the respiratory system*** |  |  |  |  |  |  |
| Higbee, D.-2021 [37] | Chronic obstructive pulmonary disease | 0.970 (0.920-1.030) | 0.400 |  |  |  |
| Meta-analysis | Lung function: FEV1 | 1.056 (0.532-2.095) | 0.496 | 0.020 | 1 | 0.000 |
| Higbee, D.-2021 [37] | Lung function: FEV1 | 1.040 (0.820-1.320) | 0.730 |  |  |  |
| Russ, T. C.-2021 [38] | Pulmonary function: FEV1 | 1.060 (0.940-1.190) | 0.355 |  |  |  |
| Meta-analysis | Lung function: FVC | 1.006 (0.448-2.264) | 0.936 | 0.462 | 1 | 0.000 |
| Higbee, D.-2021 [37] | Lung function: FVC | 1.080 (0.850-1.370) | 0.510 |  |  |  |
| Russ, T. C.-2021 [38] | Pulmonary function: FVC | 0.980 (0.850-1.140) | 0.815 |  |  |  |
| Meta-analysis | Lung function: FEV1/FVC | 1.061 (0.487-2.310) | 0.513 | 2.397 | 1 | 0.583 |
| Higbee, D.-2021 [37] | Lung function: FEV1/FVC | 0.990 (0.880-1.130) | 0.970 |  |  |  |
| Russ, T. C.-2021 [38] | Pulmonary function: FEV1/FVC ratio | 1.120 (1.020-1.230) | 0.016 |  |  |  |
| Higbee, D.-2021 [37] | Lung function: FEV1, FVC, FEV1/FVC, peak expiratory flow | 1.020 (0.910-1.130) | 0.680 |  |  |  |
| ***Disease of the digestive system*** |  |  |  |  |  |  |
| Meta-analysis | Crohn's disease | 0.998 (0.992-1.003) | 0.221 | 0.886 | 2 | 0.000 |
| Li, Q. S.-2020 [31] | Crohn's disease | 0.996 (0.992-1.001) | 0.107 |  |  |  |
| Li, Q. S.-2020 [31] | Crohn's disease | 0.998 (0.994-1.003) | 0.396 |  |  |  |
| Li, Q. S.-2020 [31] | Crohn's disease | 0.999 (0.995-1.004) | 0.796 |  |  |  |
| Li, Q. S.-2020 [31] | Ulcerative colitis | 1.004 (0.997-1.010) | 0.260 |  |  |  |
| Li, Q. S.-2020 [31] | Inflammatory bowel disease | 0.998 (0.993-1.002) | 0.304 |  |  |  |
| Li, Q. S.-2020 [31] | Celiac disease | 1.000 (0.995-1.005) | 0.977 |  |  |  |
| ***Diseases of Endocrine System*** |  |  |  |  |  |  |
| Zhuang, Q. S.-2021 [39] | Obesity class 1: BMI more than 30 kg/m2 | 0.970 (0.890-1.060) | 0.490 |  |  |  |
| Meta-analysis | T2DM | 1.006 (0.999-1.013) | 0.095 | 8.091 | 9 | 0.000 |
| Li, Q. S.-2020 [31] | T2DM | 1.009 (0.997-1.021) | 0.144 |  |  |  |
| Li, Q. S.-2020 [31] | T2DM | 1.005 (0.997-1.013) | 0.189 |  |  |  |
| Jansen, I. E.-2019 [9] | T2DM | 0.992 (0.971-1.014) | 0.428 |  |  |  |
| Thomassen, J. Q.-2020 [40] | T2DM | 1.040 (0.980-1.100) | > 0.050 |  |  |  |
| Andrews, S. J.-2021 [5] | T2DM | 1.000 (0.960-1.030) | 0.960 |  |  |  |
| Pan, Y.-2020 [41] | T2DM | 1.020 (0.970-1.070) | 0.520 |  |  |  |
| Larsson, S. C.-2017 [4] | T2DM | 1.020 (0.970-1.070) | 0.490 |  |  |  |
| Garfield, V.-2021 [42] | T2DM | 1.150 (0.870-1.520) | > 0.050 |  |  |  |
| Wang, H.-2021 [7] | T2DM (males) | 1.149 (0.423-3.122) | 0.786 |  |  |  |
| Wang, H.-2021 [7] | T2DM (females) | 2.067 (0.944-4.527) | 0.069 |  |  |  |
| ***Diabetic phenotypes*** |  |  |  |  |  |  |
| Zhou, M.-2021 [43] | IR: based on fasting insulin | 1.130 (1.039-1.228) | 0.004 |  |  |  |
| Zhou, M.-2021 [43] | IR: based on the euglycemic-hyperinsulinemic clamp and the insulin suppression test | 1.015 (1.001-1.029) | 0.030 |  |  |  |
| Pan, Y.-2020 [41] | Homeostasis model assessment-IR | 1.170 (0.400-3.370) | 0.780 |  |  |  |
| Meta-analysis | Fasting insulin | 1.158 (0.234-5.724) | 0.451 | 0.106 | 1 | 0.000 |
| Pan, Y.-2020 [41] | Fasting insulin | 1.240 (0.770-2.010) | 0.380 |  |  |  |
| Larsson, S. C.-2017 [4] | Fasting insulin | 1.130 (0.850-1.510) | 0.400 |  |  |  |
| Meta-analysis | Beta-cell function | 1.316 (0.022-78.750) | 0.551 | 6.108 | 1 | 0.836 |
| Pan, Y.-2020 [41] | Homeostasis model assessment: β-cell function | 1.920 (1.150-3.210) | 0.010 |  |  |  |
| Walter, S.-2016 [44] | Beta-cell function | 1.000 (0.940-1.070) | 0.890 |  |  |  |
| Meta-analysis | Non-fasting plasma glucose | 1.070 (0.249-4.588) | 0.662 | 1.318 | 1 | 0.241 |
| Benn, M.-2020 [45] | Non-fasting plasma glucose | 1.410 (0.820-2.430) | 0.220 |  |  |  |
| Benn, M.-2020 [45] | Non-fasting plasma glucose | 1.020 (0.920-1.130) | 0.420 |  |  |  |
| Meta-analysis | HbA1c | 0.973 (0.716-1.322) | 0.860 | 0.060 | 1 | 0.000 |
| Garfield, V.-2021 [42] | HbA1c | 1.090 (0.420-2.830) | > 0.050 |  |  |  |
| Pan, Y.-2020 [41] | HbA1c | 0.960 (0.690-1.320) | 0.780 |  |  |  |
| Larsson, S. C.-2017 [4] | Fasting glucose | 1.140 (0.990-1.320) | 0.070 |  |  |  |
| Walter, S.-2016 [44] | Overall T2DM-Genetic Risk Index | 1.010 (0.960-1.060) | 0.790 |  |  |  |
| Walter, S.-2016 [44] | Insulin sensitivity | 1.170 (1.020-1.340) | 0.020 |  |  |  |
| Walter, S.-2016 [44] | Adiposity | 0.930 (0.740-1.150) | 0.490 |  |  |  |
| Walter, S.-2016 [44] | Diabetic phenotype: other biological factors | 0.900 (0.790-1.040) | 0.140 |  |  |  |
| ***Neoplasms*** |  |  |  |  |  |  |
| Seddighi, S.-2019 [46] | Renal cell carcinoma | 0.970 (0.890-1.050) | 0.434 |  |  |  |
| Seddighi, S.-2019 [46] | Pancreatic cancer | 0.970 (0.910-1.040) | 0.429 |  |  |  |
| Seddighi, S.-2019 [46] | Upper aerodigestive tract cancer | 0.960 (0.900-1.030) | 0.235 |  |  |  |
| Seddighi, S.-2019 [46] | Urinary bladder cancer | 0.940 (0.830-1.060) | 0.325 |  |  |  |
| Seddighi, S.-2019 [46] | Lung cancer | 0.910 (0.840-0.990) | 0.019 |  |  |  |
| Seddighi, S.-2019 [46] | Smoking-related cancers: including renal cell carcinoma, pancreatic cancer, upper aerodigestive tract cancer, urinary bladder cancer, lung cancer | 0.950 (0.920-0.980) | 0.003 |  |  |  |
| Seddighi, S.-2019 [46] | Prostate cancer | 0.980 (0.950-1.010) | 0.234 |  |  |  |
| Seddighi, S.-2019 [46] | Leukemia | 0.980 (0.960-1.000) | 0.012 |  |  |  |
| Seddighi, S.-2019 [46] | Breast cancer | 0.940 (0.890-0.990) | 0.028 |  |  |  |
| Seddighi, S.-2019 [46] | Melanoma | 0.980 (0.930-1.030) | 0.447 |  |  |  |
| Seddighi, S.-2019 [46] | Lymphoma | 1.010 (0.950-1.070) | 0.764 |  |  |  |
| Seddighi, S.-2019 [46] | Ovarian cancer | 1.010 (0.960-1.060) | 0.797 |  |  |  |
| Seddighi, S.-2019 [46] | Non-smoking-related cancers: including prostate cancer, leukemia, breast cancer, melanoma, lymphoma, ovarian cancer | 0.980 (0.970-0.997) | 0.009 |  |  |  |
| Seddighi, S.-2019 [46] | All cancers: including renal cell carcinoma, pancreatic cancer, upper aerodigestive tract cancer, urinary bladder cancer, lung cancer, prostate cancer, leukemia, breast cancer, melanoma, lymphoma, ovarian cancer) | 0.980 (0.960-0.990) | <0.001 |  |  |  |
| ***Disease of immune system*** |  |  |  |  |  |  |
| Meta-analysis | RA | 0.991 (0.982-0.999) | 0.039 | 4.858 | 3 | 0.382 |
| Li, Q. S.-2020 [31] | RA | 0.991 (0.985-0.996) | 0.002 |  |  |  |
| Policicchio, S.-2017 [47] | RA | 1.018 (0.980–1.060) | 0.354 |  |  |  |
| Andrews, S. J.-2019 [48] | RA | 0.990 (0.970-1.010) | 0.370 |  |  |  |
| Bae, S. C.-2019 [49] | RA | 0.962 (0.930-0.994) | 0.021 |  |  |  |
| Lee, Y. H.-2019 [50] | Gout | 1.013 (0.980-1.047) | 0.445 |  |  |  |
| ***Disease of herpes virus infection*** |  |  |  |  |  |  |
| Meta-analysis | HSV infection | 1.097 (0.476-2.530) | 0.680 | 1.871 | 2 | 0.000 |
| Kwok, M. K.-2021 [51] | HSV infection | 1.903 (0.759-4.769) | 0.170 |  |  |  |
| Kwok, M. K.-2021 [51] | HSV infection | 1.002 (0.981-1.024) | 0.860 |  |  |  |
| Kwok, M. K.-2021 [51] | HSV infection | 0.999 (0.998-41.001) | 0.480 |  |  |  |
| Meta-analysis | Chickenpox | 1.006 (0.148-6.829) | 0.975 | 4.279 | 1 | 0.766 |
| Huang, S. Y.-2021 [52] | Chickenpox | 0.846 (0.654-1.094) | 0.202 |  |  |  |
| Huang, S. Y.-2021 [52] | Chickenpox | 1.147 (1.007-1.307) | 0.039 |  |  |  |
| Meta-analysis | Shingles | 0.958 (0.289-3.169) | 0.726 | 10.845 | 1 | 0.908 |
| Huang, S. Y.-2021 [52] | Shingles | 0.867 (0.784-0.958) | 0.005 |  |  |  |
| Huang, S. Y.-2021 [52] | Shingles | 1.047 (0.995-1.101) | 0.075 |  |  |  |
| Meta-analysis | Cold sores | 0.962 (0.281-3.302) | 0.761 | 0.001 | 1 | 0.000 |
| Huang, S. Y.-2021 [52] | Cold sores | 0.959 (0.733-1.254) | 0.758 |  |  |  |
| Huang, S. Y.-2021 [52] | Cold sores | 0.966 (0.738-1.265) | 0.802 |  |  |  |
| Meta-analysis | Mononucleosis | 1.463 (0.340-6.300) | 0.187 | 0.418 | 1 | 0.000 |
| Huang, S. Y.-2021 [52] | Mononucleosis | 1.634 (1.092-2.446) | 0.017 |  |  |  |
| Huang, S. Y.-2021 [52] | Mononucleosis | 1.392 (1.061-1.826) | 0.017 |  |  |  |
| ***Other diseases or status*** |  |  |  |  |  |  |
| Meta-analysis | Periodontitis | 1.039 (0.469-2.302) | 0.650 | 3.447 | 1 | 0.710 |
| Sun, Y. Q.-2020 [53] | Periodontitis | 1.100 (1.020-1.190) | 0.020 |  |  |  |
| Sun, Y. Q.-2020 [53] | Periodontitis | 0.970 (0.870-1.080) | 0.590 |  |  |  |
| Andrews, S. J.-2021 [5] | Hearing difficulties | 1.020 (0.700-1.470) | 0.940 |  |  |  |
| Li, Q. S.-2020 [31] | Myopia | 1.096 (0.820-1.467) | 0.535 |  |  |  |
| **Dietary intake** |  |  |  |  |  |  |
| Meta-analysis | Milk intake | 0.965 (0.809-1.152) | 0.240 | 0.390 | 1 | 0.000 |
| Zhang, Z.-2021 [54] | Milk intake | 0.970 (0.940-1.000) | 0.079 |  |  |  |
| Zhang, Z.-2021 [54] | Milk intake | 0.950 (0.900-1.010) | 0.098 |  |  |  |
| Meta-analysis | Coffee consumption | 1.188 (0.905-1.559) | 0.112 | 1.194 | 2 | 0.000 |
| Zhang, Z.-2021 [55] | Coffee consumption | 1.260 (1.050-1.510) | 0.014 |  |  |  |
| Zhang, Z.-2021 [55] | Coffee consumption | 1.030 (0.750-1.420) | 0.852 |  |  |  |
| Kwok, M. K.-2016 [56] | Habitual coffee consumption | 1.170 (0.960-1.430) | > 0.050 |  |  |  |
| Andrews, S. J.-2021 [5] | Fish and plant diet | 1.090 (0.910-1.300) | 0.370 |  |  |  |
| Andrews, S. J.-2021 [5] | Meat diet | 0.990 (0.800-1.220) | 0.910 |  |  |  |
| ***Nutrients-mineral nutrition*** |  |  |  |  |  |  |
| Meta-analysis | Serum calcium | 0.712 (0.467-1.086) | 0.083 | 1.521 | 3 | 0.000 |
| Shi, Y.-2021 [57] | Serum calcium | 0.760 (0.510-1.150) | 0.194 |  |  |  |
| Shi, Y.-2021 [57] | Serum calcium | 1.140 (0.360-3.640) | 0.818 |  |  |  |
| He, Y.-2020 [58] | Serum calcium | 0.570 (0.350-0.950) | 0.031 |  |  |  |
| Cheng, W. W.-2019 [59] | Serum calcium | 0.740 (0.450-1.220) | 0.230 |  |  |  |
| Cheng, W. W.-2019 [59] | Magnesium | 0.430 (0.080-2.440) | 0.340 |  |  |  |
| Cheng, W. W.-2019 [59] | Iron | 1.040 (0.940-1.140) | 0.480 |  |  |  |
| Cheng, W. W.-2019 [59] | Copper | 0.870 (0.750-1.000) | 0.050 |  |  |  |
| Cheng, W. W.-2019 [59] | Zinc | 0.990 (0.850-1.140) | 0.850 |  |  |  |
| ***Nutrients-polyunsaturated fatty acids*** |  |  |  |  |  |  |
| Meta-analysis | Arachidonic acid (AA, 20:4n6) | 1.002 (0.977-1.028) | 0.488 | 0.202 | 1 | 0.000 |
| Tomata, Y.-2020 [60] | Arachidonic acid (AA, 20:4n6) | 1.002 (0.998-1.006) | 0.461 |  |  |  |
| Tomata, Y.-2020 [60] | Arachidonic acid (AA, 20:4n6) | 1.010 (0.980-1.050) | 0.471 |  |  |  |
| Meta-analysis | Eicosapentaenoic acid (EPA, 20:5n3) | 1.003 (0.911-1.104) | 0.786 | 0.301 | 1 | 0.000 |
| Tomata, Y.-2020 [60] | Eicosapentaenoic acid (EPA, 20:5n3) | 1.002 (0.987-1.017) | 0.823 |  |  |  |
| Tomata, Y.-2020 [60] | Eicosapentaenoic acid (EPA, 20:5n3) | 1.030 (0.930-1.130) | 0.610 |  |  |  |
| Meta-analysis | Linoleic acid (LA, 18:2n6) | 1.000 (0.996-1.004) | 0.772 | NA | 2 | NA |
| Tomata, Y.-2020 [60] | Linoleic acid (LA, 18:2n6) | 0.997 (0.992-1.003) | 0.696 |  |  |  |
| Tomata, Y.-2020 [60] | Linoleic acid (LA, 18:2n6) | 0.980 (0.930-1.030) | 0.476 |  |  |  |
| Wang, Z. -2020 [1] | Linoleic acid | 1.000 (1.000-1.000) | 0.040 |  |  |  |
| Meta-analysis | α-Linolenic acid (ALA, 18:3n3) | 0.994 (0.911-1.085) | 0.555 | 0.557 | 1 | 0.000 |
| Tomata, Y.-2020 [60] | α-Linolenic acid (ALA, 18:3n3) | 0.995 (0.982-1.009) | 0.470 |  |  |  |
| Tomata, Y.-2020 [60] | α-Linolenic acid (ALA, 18:3n3) | 0.960 (0.880-1.060) | 0.427 |  |  |  |
| Meta-analysis | Docosapentaenoic acid (DPA, 22:5n3) | 1.003 (0.953-1.056) | 0.546 | 0.782 | 1 | 0.000 |
| Tomata, Y.-2020 [60] | Docosapentaenoic acid (DPA, 22:5n3) | 1.003 (0.995-1.011) | 0.477 |  |  |  |
| Tomata, Y.-2020 [60] | Docosapentaenoic acid (DPA, 22:5n3) | 1.030 (0.970-1.090) | 0.375 |  |  |  |
| Meta-analysis | Docosahexaenoic acid (DHA, 22:6n3) | 0.997 (0.929-1.069) | 0.854 | 0.044 | 2 | 0.000 |
| Tomata, Y.-2020 [60] | Docosahexaenoic acid (DHA, 22:6n3) | 0.997 (0.965-1.031) | 0.884 |  |  |  |
| Tomata, Y.-2020 [60] | Docosahexaenoic acid (DHA, 22:6n3) | 1.010 (0.810-1.250) | 0.944 |  |  |  |
| Lord, J.-2021 [59] | 22:6, Docosahexaenoic acid | 0.985 (0.860-1.128) | 0.825 |  |  |  |
| Lord, J.-2021 [61] | Omega-3 fatty acids | 1.061 (0.936-1.203) | 0.354 |  |  |  |
| Lord, J.-2021 [61] | Mono-unsaturated fatty acids | 1.086 (0.962-1.226) | 0.182 |  |  |  |
| Lord, J.-2021 [61] | Other polyunsaturated fatty acids than 18:2 | 1.030 (0.964-1.101) | 0.381 |  |  |  |
| ***Nutrients-amino acid*** |  |  |  |  |  |  |
| Meta-analysis | Total homocysteine | 0.998 (0.706-1.410) | 0.951 | 0.023 | 1 | 0.000 |
| Larsson, S. C.-2017 [4] | Total homocysteine | 0.990 (0.880-1.110) | 0.860 |  |  |  |
| Roostaei, T.-2018 [62] | Total homocysteine | 1.000 (0.940-1.060) | 0.960 |  |  |  |
| Adams, C. D.-2020 [63] | Circulating glutamine | 0.830 (0.710-0.970) | 0.020 |  |  |  |
| Larsson, S. C.-2017 [64] | Isoleucine | 1.350 (1.080-1.690) | 0.007 |  |  |  |
| Larsson, S. C.-2017 [64] | Leucine | 1.160 (0.780-1.720) | 0.460 |  |  |  |
| Larsson, S. C.-2017 [64] | Valine | 1.130 (0.820-1.570) | 0.460 |  |  |  |
| ***Nutrients-vitamin*** |  |  |  |  |  |  |
| Meta-analysis | Vitamin B12 | 1.022 (0.595-1.756) | 0.696 | 1.674 | 1 | 0.403 |
| Gagliano Taliun, S. A.-2019 [65] | Vitamin B12 levels | 1.000 (0.980-1.020) | 0.990 |  |  |  |
| Larsson, S. C.-2017 [4] | Serum vitamin B12 | 1.110 (0.950-1.300) | 0.180 |  |  |  |
| Meta-analysis | Vitamin C | 0.958 (0.907-1.011) | 0.097 | 23.859 | 5 | 0.790 |
| Liu, H.-2021 [66] | Plasma vitamin C | 0.930 (0.800-1.090) | 0.385 |  |  |  |
| Liu, H.-2021 [66] | Plasma vitamin C | 0.930 (0.880-0.980) | 0.007 |  |  |  |
| Liu, H.-2021 [66] | Plasma vitamin C | 0.890 (0.840-0.940) | <0.001 |  |  |  |
| Liu, H.-2021 [66] | Plasma vitamin C | 1.020 (0.920-1.120) | 0.759 |  |  |  |
| Chen, L.-2021 [67] | Plasma vitamin C | 0.968 (0.946-0.991) | 0.006 |  |  |  |
| Williams, D. M.-2019 [68] | Circulating ascorbate: vitamin C | 1.000 (0.990-1.020) | > 0.050 |  |  |  |
| Meta-analysis | 25(OH)D | 0.861 (0.773-0.959) | 0.018 | 7.882 | 4 | 0.493 |
| Wang, L.-2020 [69] | Circulating Vitamin D Levels: 25(OH)D levels | 0.620 (0.460-0.840) | 0.002 |  |  |  |
| Wang, L.-2020 [69] | Circulating Vitamin D Levels: 25(OH)D levels | 0.880 (0.730-1.060) | 0.190 |  |  |  |
| Larsson, S. C.-2017 [4] | Circulating Vitamin D Levels: 25(OH)D levels | 0.920 (0.850-0.980) | 0.010 |  |  |  |
| Larsson, S. C.-2018 [70] | Serum 25(OH)D | 0.860 (0.780-0.940) | 0.002 |  |  |  |
| Mokry, L. E.-2016 [71] | Vitamin D: 25(OH)D levels | 1.250 (1.030-1.510) | 0.021 |  |  |  |
| Williams, D. M.-2019 [68] | Retinol: vitamin A | 1.050 (0.940-1.180) | > 0.050 |  |  |  |
| Williams, D. M.-2019 [68] | β-carotene | 1.020 (1.000-1.030) | > 0.050 |  |  |  |
| Larsson, S. C.-2017 [4] | Serum folate: vitamin B9 | 0.980 (0.720-1.330) | 0.890 |  |  |  |
| Liu, G.-2018 [72] | Circulating vitamin E levels | 0.960 (0.470-1.940) | 0.936 |  |  |  |
| **Biochemical index** |  |  |  |  |  |  |
| Lord, J.-2021 [61] | Mean diameter for HDL particles | 0.887 (0.797-0.987) | 0.027 |  |  |  |
| Lord, J.-2021 [61] | Total cholesterol in large HDL | 0.891 (0.803-0.988) | 0.029 |  |  |  |
| Lord, J.-2021 [61] | Cholesterol esters in large HDL | 0.913 (0.833-1.001) | 0.052 |  |  |  |
| Lord, J.-2021 [61] | Free cholesterol in large HDL | 0.891 (0.808-0.983) | 0.021 |  |  |  |
| Lord, J.-2021 [61] | Total lipids in large HDL | 0.914 (0.839-0.996) | 0.040 |  |  |  |
| Lord, J.-2021 [61] | Concentration of large HDL particles | 0.913 (0.838-0.995) | 0.038 |  |  |  |
| Lord, J.-2021 [61] | Phospholipids in large HDL | 0.912 (0.836-0.995) | 0.039 |  |  |  |
| Lord, J.-2021 [61] | Triglycerides in small HDL | 1.047 (0.945-1.159) | 0.381 |  |  |  |
| Lord, J.-2021 [61] | Total cholesterol in very large HDL | 0.883 (0.790-0.988) | 0.029 |  |  |  |
| Lord, J.-2021 [61] | Cholesterol esters in very large HDL | 0.907 (0.794-1.036) | 0.149 |  |  |  |
| Lord, J.-2021 [61] | Free cholesterol in very large HDL | 0.859 (0.783-0.943) | 0.001 |  |  |  |
| Lord, J.-2021 [61] | Total lipids in very large HDL | 0.881 (0.801-0.968) | 0.008 |  |  |  |
| Lord, J.-2021 [61] | Concentration of very large HDL particles | 0.866 (0.786-0.955) | 0.004 |  |  |  |
| Lord, J.-2021 [61] | Phospholipids in very large HDL | 0.886 (0.810-0.969) | 0.008 |  |  |  |
| Meta-analysis | LDL-C | 1.079 (1.012-1.150) | 0.025 | 20.436 | 9 | 0.560 |
| Li, Q. S.-2020 [31] | LDL-C | 1.064 (1.025-1.104) | 0.001 |  |  |  |
| Li, Q. S.-2020 [31] | LDL-C | 1.070 (1.014-1.130) | 0.013 |  |  |  |
| Zhang, Q.-2020 [2] | LDL-C | 1.193 (1.097-1.296) | <0.001 |  |  |  |
| Proitsi, P.-2014 [73] | LDL-C | 0.807 (0.570 - 1.140) | 0.225 |  |  |  |
| Proitsi, P.-2014 [73] | LDL-C | 1.309 (0.240 - 4.220) | 0.677 |  |  |  |
| Proitsi, P.-2014 [73] | LDL-C | 3.652 (0.240 - 4.220) | 0.077 |  |  |  |
| Benn, M.-2017 [74] | LDL-C | 0.830 (0.750-0.920) | < 0.001 |  |  |  |
| Zhu, Z.-2018 [26] | LDL-C | 1.030 (0.950-1.120) | 0.470 |  |  |  |
| Williams, D. M.-2020 [75] | LDL-C | 0.980 (0.880-1.100) | 0.750 |  |  |  |
| Williams, D. M.-2020 [75] | LDL-C | 1.010 (0.740-1.380) | 0.960 |  |  |  |
| Meta-analysis | HDL-C | 0.930 (0.822-1.052) | 0.200 | 27.736 | 6 | 0.784 |
| Andrews, S. J.-2021 [5] | HDL-C | 0.990 (0.920-1.060) | 0.750 |  |  |  |
| Zhang, Q.-2020 [2] | HDL-C | 0.761 (0.697-0.830) | <0.001 |  |  |  |
| Li, Q. S.-2020 [31] | HDL-C | 0.962 (0.914-1.013) | 0.141 |  |  |  |
| Li, Q. S.-2020 [31] | HDL-C | 0.960 (0.895-1.029) | 0.253 |  |  |  |
| Proitsi, P.-2014 [71] | HDL-C | 0.947 (0.760-1.180) | 0.644 |  |  |  |
| Proitsi, P.-2014 [71] | HDL-C | 1.559 (0.390-2.560) | 0.265 |  |  |  |
| Proitsi, P.-2014 [71] | HDL-C | 1.514 (0.390-2.550) | 0.394 |  |  |  |
| Meta-analysis | TG | 1.077 (0.943-1.230) | 0.173 | 2.615 | 3 | 0.000 |
| Andrews, S. J.-2021 [5] | TG | 1.070 (0.980-1.170) | 0.110 |  |  |  |
| Proitsi, P.-2014 [73] | TG | 1.126 (0.900-1.410) | 0.309 |  |  |  |
| Proitsi, P.-2014 [73] | TG | 0.619 (0.390-2.580) | 0.236 |  |  |  |
| Proitsi, P.-2014 [73] | TG | 1.795 (0.390-2.580) | 0.228 |  |  |  |
| Meta-analysis | TC | 1.081 (0.978-1.195) | 0.113 | 32.785 | 9 | 0.725 |
| Dunk, M. M.-2021 [76] | TC | 1.210 (1.090-54.050) | < 0.050 |  |  |  |
| Li, Q. S.-2020 [31] | TC | 1.082 (1.036-1.132) | <0.001 |  |  |  |
| Li, Q. S.-2020 [31] | TC | 1.094 (1.022-1.170) | 0.009 |  |  |  |
| Wang, H.-2021 [7] | TC (male) | 1.071 (0.824-1.393) | 0.605 |  |  |  |
| Wang, H.-2021 [7] | TC (female) | 1.029 (0.836-1.267) | 0.783 |  |  |  |
| Andrews, S. J.-2021 [5] | TC | 0.970 (0.910-1.040) | 0.440 |  |  |  |
| Zhang, Q.-2020 [2] | TC | 1.300 (1.196-1.412) | <0.001 |  |  |  |
| Dunk, M. M.-2021 [76] | TC | 0.911 (0.710-1.170) | 0.469 |  |  |  |
| Dunk, M. M.-2021 [76] | TC | 0.961 (0.360-2.780) | 0.935 |  |  |  |
| Dunk, M. M.-2021 [76] | TC | 2.066 (0.350-2.780) | 0.165 |  |  |  |
| Kunutsor, S. K.-2018 [77] | Serum gamma-glutamyltransferase | 1.090 (0.980-1.220) | 0.100 |  |  |  |
| Fani, L.-2021 [78] | Fibrinogen | 1.614 (0.835-3.124) | 0.154 |  |  |  |
| Meta-analysis | TSH | 0.986 (0.978-0.994) | 0.012 | 0.217 | 3 | 0.000 |
| Li, G. H.-2021 [79] | TSH (overall) | 0.988 (0.977-0.998) | 0.017 |  |  |  |
| Li, G. H.-2021 [79] | TSH (overall) | 0.986 (0.976 -0.996) | 0.008 |  |  |  |
| Li, G. H.-2021 [79] | TSH (women) | 0.985 (0.975-0.996) | 0.008 |  |  |  |
| Li, G. H.-2021 [79] | TSH (men) | 0.985 (0.976-0.995) | 0.003 |  |  |  |
| Meta-analysis | FT4 | 1.001 (0.979-1.023) | 0.892 | 0.176 | 2 | 0.000 |
| Li, G. H.-2021 [79] | FT4 (overall) | 1.003 (0.982-1.024) | 0.787 |  |  |  |
| Li, G. H.-2021 [79] | FT4 (overall) | 1.002 (0.987-1.018) | 0.757 |  |  |  |
| Li, G. H.-2021 [79] | FT4 (overall) | 0.998 (0.982-1.015) | 0.851 |  |  |  |
| Romo, M. L.-2017 [80] | Circulating leptin levels | 0.990 (0.550-1.780) | > 0.050 |  |  |  |
| Romo, M. L.-2017 [80] | Soluble plasma leptin receptor levels | 1.080 (0.830-1.410) | > 0.050 |  |  |  |
| Syed, A. A. S.-2020 [81] | Testosterone | 0.998 (0.943-1.056) | 0.935 |  |  |  |
| Larsson, S. C.-2018 [70] | Serum parathyroid hormone | 1.110 (0.970-1.260) | 0.120 |  |  |  |
| Meta-analysis | Uric acid | 1.025 (0.742-1.417) | 0.505 | 0.037 | 1 | 0.000 |
| Williams, D. M.-2019 [68] | Urate | 1.030 (0.960-1.100) | > 0.050 |  |  |  |
| Yuan, H.-2018 [82] | Serum uric acid | 1.020 (0.950-1.100) | 0.560 |  |  |  |
| Meta-analysis | Lp(a) | 0.941 (0.912-0.971) | < 0.001 | 0.080 | 1 | 0.000 |
| Larsson, S. C.-2020 [83] | Lp(a) | 0.960 (0.840-1.110) | 0.609 |  |  |  |
| Pan, Y.-2019 [84] | Lp(a) | 0.940 (0.910-0.970) | < 0.001 |  |  |  |
| Lord, J.-2021 [61] | Glycoprotein acetyls | 1.199 (1.045-1.375) | 0.010 |  |  |  |
| **Inflammatory cytokines** |  |  |  |  |  |  |
| Meta-analysis | Vascular endothelial growth factor | 1.002 (0.949-1.057) | 0.754 | 0.611 | 1 | 0.000 |
| Yeung, C. H. C.-2021 [85] | Vascular endothelial growth factor | 1.001 (0.993-1.010) | 0.790 |  |  |  |
| Fani, L.-2021 [78] | Vascular endothelial growth factor | 1.017 (0.978-1.057) | 0.405 |  |  |  |
| Meta-analysis | PDGFB/PDGFbb | 1.002 (0.985-1.019) | 0.651 | 1.546 | 2 | 0.000 |
| Yeung, C. H. C.-2021 [85] | PDGFB/PDGFbb | 1.006 (0.996-1.016) | 0.270 |  |  |  |
| Yeung, C. H. C.-2021 [85] | PDGFB/PDGFbb | 0.998 (0.987-1.009) | 0.760 |  |  |  |
| Fani, L.-2021 [78] | PDGFB/PDGFbb | 0.979 (0.913-1.050) | 0.550 |  |  |  |
| Meta-analysis | CLEC11A/SCGFb | 0.999 (0.985-1.012) | 0.700 | 0.316 | 2 | 0.000 |
| Yeung, C. H. C.-2021 [85] | CLEC11A/SCGFb | 1.000 (0.992-1.009) | 0.930 |  |  |  |
| Yeung, C. H. C.-2021 [85] | CLEC11A/SCGFb | 0.997 (0.988-1.007) | 0.560 |  |  |  |
| Fani, L.-2021 [78] | SCGFb | 0.988 (0.925-1.054) | 0.712 |  |  |  |
| Meta-analysis | KITLG/SCF | 0.998 (0.979-1.018) | 0.764 | 0.314 | 2 | 0.000 |
| Yeung, C. H. C.-2021 [85] | KITLG/SCF | 0.996 (0.977-1.015) | 0.660 |  |  |  |
| Yeung, C. H. C.-2021 [85] | KITLG/SCF | 0.999 (0.989-1.009) | 0.890 |  |  |  |
| Fani, L.-2021 [78] | SCF | 1.048 (0.863-1.273) | 0.635 |  |  |  |
| Meta-analysis | FGFBasic | 0.998 (0.966-1.031) | 0.813 | 0.504 | 2 | 0.000 |
| Yeung, C. H. C.-2021 [85] | FGFBasic | 0.994 (0.976-1.013) | 0.530 |  |  |  |
| Yeung, C. H. C.-2021 [85] | FGFBasic | 1.005 (0.980-1.031) | 0.690 |  |  |  |
| Fani, L.-2021 [78] | FGFBasic | 1.016 (0.839-1.231) | 0.867 |  |  |  |
| Meta-analysis | Granulocyte colony-stimulating factor | 0.996 (0.908-1.091) | 0.662 | 0.937 | 1 | 0.000 |
| Yeung, C. H. C.-2021 [85] | Granulocyte colony-stimulating factor | 1.002 (0.983-1.021) | 0.860 |  |  |  |
| Yeung, C. H. C.-2021 [85] | Granulocyte colony-stimulating factor | 0.988 (0.967-1.009) | 0.270 |  |  |  |
| Meta-analysis | HGF | 0.998 (0.976-1.020) | 0.701 | 0.252 | 2 | 0.000 |
| Yeung, C. H. C.-2021 [85] | HGF | 0.999 (0.984-1.014) | 0.870 |  |  |  |
| Yeung, C. H. C.-2021 [85] | HGF | 0.997 (0.984-1.011) | 0.700 |  |  |  |
| Fani, L.-2021 [78] | HGF | 0.963 (0.829-1.120) | 0.626 |  |  |  |
| Meta-analysis | Macrophage colony-stimulating factor | 0.998 (0.950-1.049) | 0.698 | 0.267 | 1 | 0.000 |
| Yeung, C. H. C.-2021 [85] | Macrophage colony-stimulating factor | 0.997 (0.989-1.006) | 0.500 |  |  |  |
| Yeung, C. H. C.-2021 [85] | Macrophage colony-stimulating factor | 1.002 (0.985-1.019) | 0.840 |  |  |  |
| Williams, D. M.-2018 [86] | The magnitude of effect of variation in IGF1 | 1.170 (0.920-1.490) | > 0.050 |  |  |  |
| Handy, A.-2021 [87] | Insulin-like growth factor-binding protein 2 | 0.990 (0.920-1.080) | 0.898 |  |  |  |
| Williams, D. M.-2018 [86] | The magnitude of effect of variation in insulin-like growth factor-binding protein 3 | 0.990 (0.900-1.080) | > 0.050 |  |  |  |
| Meta-analysis | Macrophage inflammatory protein-1b | 1.009 (0.843-1.208) | 0.634 | 5.506 | 1 | 0.818 |
| Yeung, C. H. C.-2021 [85] | Macrophage inflammatory protein-1b | 0.997 (0.990-1.004) | 0.350 |  |  |  |
| Fani, L.-2021 [78] | Macrophage inflammatory protein-1b | 1.026 (1.003-1.050) | 0.024 |  |  |  |
| Meta-analysis | CCL11/Eotaxin-1 | 0.997 (0.982-1.012) | 0.440 | 0.518 | 2 | 0.000 |
| Yeung, C. H. C.-2021 [85] | Eotaxin | 0.998 (0.988-1.008) | 0.690 |  |  |  |
| Yeung, C. H. C.-2021 [85] | CCL11/Eotaxin-1 | 0.995 (0.985-1.004) | 0.280 |  |  |  |
| Fani, L.-2021 [78] | Eotaxin | 1.015 (0.954-1.080) | 0.637 |  |  |  |
| Meta-analysis | Monocyte chemotactic protein-1 | 1.004 (0.922-1.094) | 0.642 | 0.024 | 1 | 0.000 |
| Yeung, C. H. C.-2021 [85] | Monocyte chemotactic protein-1 | 1.004 (0.990-1.017) | 0.590 |  |  |  |
| Fani, L.-2021 [78] | Monocyte chemotactic protein-1 | 1.009 (0.949-1.073) | 0.771 |  |  |  |
| Meta-analysis | CCL27/CTACK | 1.002 (0.988-1.017) | 0.581 | 0.277 | 2 | 0.000 |
| Yeung, C. H. C.-2021 [85] | CCL27/CTACK | 1.002 (0.993-1.010) | 0.660 |  |  |  |
| Yeung, C. H. C.-2021 [85] | CCL27/CTACK | 1.002 (0.992-1.013) | 0.660 |  |  |  |
| Fani, L.-2021 [78] | CTACK | 1.019 (0.957-1.084) | 0.553 |  |  |  |
| Meta-analysis | Regulated on activation | 0.999 (0.941-1.062) | 0.927 | 0.099 | 1 | 0.000 |
| Yeung, C. H. C.-2021 [85] | Regulated on activation (RANTES) | 1.001 (0.988-1.015) | 0.880 |  |  |  |
| Yeung, C. H. C.-2021 [85] | Regulated on activation | 0.998 (0.985-1.011) | 0.780 |  |  |  |
| Meta-analysis | Macrophage inflammatory protein-1a | 1.004 (0.942-1.071) | 0.543 | 0.088 | 1 | 0.000 |
| Yeung, C. H. C.-2021 [85] | Macrophage inflammatory protein-1a | 1.006 (0.991-1.020) | 0.450 |  |  |  |
| Yeung, C. H. C.-2021 [85] | Macrophage inflammatory protein-1a | 1.003 (0.990-1.017) | 0.630 |  |  |  |
| Meta-analysis | CXCL1/GROa | 1.000 (0.988-1.012) | 0.955 | 0.250 | 2 | 0.000 |
| Yeung, C. H. C.-2021 [85] | CXCL1/GROa | 1.000 (0.992-1.008) | 0.970 |  |  |  |
| Yeung, C. H. C.-2021 [85] | CXCL1/GROa | 1.000 (0.993-1.008) | 0.910 |  |  |  |
| Fani, L.-2021 [78] | GRO-α | 1.011 (0.969-1.055) | 0.612 |  |  |  |
| Meta-analysis | Monocyte-specific chemokine 3 | 1.002 (0.948-1.059) | 0.759 | 0.126 | 1 | 0.000 |
| Yeung, C. H. C.-2021 [85] | Monocyte-specific chemokine 3 | 1.005 (0.985-1.025) | 0.610 |  |  |  |
| Yeung, C. H. C.-2021 [85] | Monocyte-specific chemokine 3 | 1.001 (0.992-1.011) | 0.840 |  |  |  |
| Meta-analysis | IP-10/CXCL10 | 1.013 (0.987-1.039) | 0.167 | 0.585 | 2 | 0.000 |
| Yeung, C. H. C.-2021 [85] | IP-10/CXCL10 | 1.017 (0.993-1.042) | 0.160 |  |  |  |
| Yeung, C. H. C.-2021 [85] | IP-10/CXCL10 | 1.011 (0.998-1.025) | 0.100 |  |  |  |
| Fani, L.-2021 [78] | IP-10/CXCL10 | 1.062 (0.917-1.229) | 0.426 |  |  |  |
| Meta-analysis | Monokine induced by interferon gamma | 1.047 (0.444-2.473) | 0.618 | 2.181 | 1 | 0.542 |
| Yeung, C. H. C.-2021 [85] | Monokine induced by interferon gamma | 1.005 (0.996-1.015) | 0.290 |  |  |  |
| Fani, L.-2021 [78] | Monokine induced by interferon gamma | 1.170 (0.957-1.432) | 0.126 |  |  |  |
| Yeung, C. H. C.-2021 [85] | Monokine induced by interferon gamma (CXCL9.1 1593.21.3)) | 0.995 (0.981-1.010) | 0.520 |  |  |  |
| Yeung, C. H. C.-2021 [85] | Monokine induced by interferon gamma (CXCL9 (CXCL9.9188.119.3)) | 0.998 (0.982-1.015) | 0.840 |  |  |  |
| Yeung, C. H. C.-2021 [85] | Stromal cell-derived factor-1 alpha | 0.988 (0.968-1.010) | 0.280 |  |  |  |
| Fani, L.-2021 [78] | IL1 | 1.089 (0.869-1.363) | 0.461 |  |  |  |
| Meta-analysis | IL1B | 0.992 (0.858-1.148) | 0.628 | 2.639 | 1 | 0.621 |
| Yeung, C. H. C.-2021 [85] | IL1B | 0.979 (0.956-1.003) | 0.090 |  |  |  |
| Yeung, C. H. C.-2021 [85] | IL1B | 1.002 (0.988-1.017) | 0.760 |  |  |  |
| Meta-analysis | lL1ra | 0.986 (0.899-1.081) | 0.303 | 0.025 | 1 | 0.000 |
| Yeung, C. H. C.-2021 [85] | lL1ra | 0.986 (0.972-1.000) | 0.040 |  |  |  |
| Tsui, A.-2018 [88] | lL1ra | 1.020 (0.640-1.490) | 0.850 |  |  |  |
| Meta-analysis | IL2 | 1.000 (0.956-1.047) | 0.990 | 0.151 | 1 | 0.000 |
| Yeung, C. H. C.-2021 [85] | IL2 | 0.998 (0.986-1.011) | 0.810 |  |  |  |
| Yeung, C. H. C.-2021 [85] | IL2 | 1.001 (0.993-1.010) | 0.790 |  |  |  |
| Meta-analysis | IL2RA | 0.994 (0.978-1.009) | 0.228 | 0.440 | 2 | 0.000 |
| Yeung, C. H. C.-2021 [85] | IL2RA | 0.989 (0.973-1.005) | 0.190 |  |  |  |
| Yeung, C. H. C.-2021 [85] | IL2RA | 0.995 (0.987-1.003) | 0.210 |  |  |  |
| Fani, L.-2021 [78] | IL2RA | 0.991 (0.935-1.050) | 0.761 |  |  |  |
| Meta-analysis | IL4 | 0.994 (0.945-1.045) | 0.355 | 0.059 | 1 | 0.000 |
| Yeung, C. H. C.-2021 [85] | IL4 | 0.995 (0.982-1.008) | 0.460 |  |  |  |
| Yeung, C. H. C.-2021 [85] | IL4 | 0.993 (0.983-1.002) | 0.140 |  |  |  |
| Meta-analysis | lL5 | 0.994 (0.973-1.015) | 0.341 | 1.099 | 2 | 0.000 |
| Yeung, C. H. C.-2021 [85] | lL5 | 0.993 (0.978-1.008) | 0.360 |  |  |  |
| Yeung, C. H. C.-2021 [85] | lL5 | 0.995 (0.983-1.008) | 0.470 |  |  |  |
| Fani, L.-2021 [78] | lL5 | 0.900 (0.745-1.088) | 0.278 |  |  |  |
| Meta-analysis | IL6 | 0.993 (0.885-1.115) | 0.595 | 0.189 | 1 | 0.000 |
| Yeung, C. H. C.-2021 [85] | IL6 | 0.991 (0.971-1.012) | 0.420 |  |  |  |
| Tsui, A.-2018 [88] | IL6 | 1.000 (0.960-1.030) | 0.780 |  |  |  |
| Yeung, C. H. C.-2021 [85] | IL6 (IL6.2573.20.15) | 1.004 (0.994-1.015) | 0.420 |  |  |  |
| Yeung, C. H. C.-2021 [85] | IL6 (IL6.4673.13.2) | 1.000 (0.985-1.014) | 0.950 |  |  |  |
| Meta-analysis | IL7 | 1.001 (0.984-1.018) | 0.899 | 1.548 | 2 | 0.000 |
| Yeung, C. H. C.-2021 [85] | IL7 | 1.001 (0.990-1.011) | 0.910 |  |  |  |
| Yeung, C. H. C.-2021 [85] | IL7 | 1.001 (0.990-1.013) | 0.820 |  |  |  |
| Fani, L.-2021 [78] | IL7 | 0.947 (0.868-1.033) | 0.226 |  |  |  |
| Yeung, C. H. C.-2021 [85] | IL8 | 0.997 (0.983-1.012) | 0.730 |  |  |  |
| Yeung, C. H. C.-2021 [85] | IL9 (IL9.5834.18.3) | 1.004 (0.989-1.019) | 0.620 |  |  |  |
| Yeung, C. H. C.-2021 [85] | IL9 (IL9.8379.35.4) | 1.007 (0.991-1.024) | 0.370 |  |  |  |
| Yeung, C. H. C.-2021 [85] | IL9 | 0.992 (0.978-1.006) | 0.240 |  |  |  |
| Meta-analysis | IL10 | 0.991 (0.966-1.016) | 0.253 | 2.040 | 2 | 0.020 |
| Yeung, C. H. C.-2021 [85] | IL10 | 0.996 (0.986-1.007) | 0.510 |  |  |  |
| Yeung, C. H. C.-2021 [85] | IL10 | 0.984 (0.970-0.999) | 0.030 |  |  |  |
| Fani, L.-2021 [78] | IL10 | 0.967 (0.885-1.055) | 0.454 |  |  |  |
| Meta-analysis | IL12p70 | 0.996 (0.929-1.068) | 0.605 | 0.658 | 1 | 0.000 |
| Yeung, C. H. C.-2021 [85] | IL12p70 | 0.997 (0.986-1.008) | 0.610 |  |  |  |
| Fani, L.-2021 [78] | IL12p70 | 0.975 (0.925-1.028) | 0.360 |  |  |  |
| Meta-analysis | IL13 | 1.000 (0.983-1.017) | 0.999 | 1.044 | 2 | 0.000 |
| Yeung, C. H. C.-2021 [85] | IL13 | 0.997 (0.987-1.008) | 0.620 |  |  |  |
| Yeung, C. H. C.-2021 [85] | IL13 | 1.003 (0.991-1.015) | 0.670 |  |  |  |
| Fani, L.-2021 [78] | IL13 | 1.021 (0.964-1.083) | 0.478 |  |  |  |
| Meta-analysis | IL16 | 0.998 (0.986-1.010) | 0.551 | 0.389 | 2 | 0.000 |
| Yeung, C. H. C.-2021 [85] | IL16 | 1.000 (0.990-1.009) | 0.930 |  |  |  |
| Yeung, C. H. C.-2021 [85] | IL16 | 0.997 (0.990-1.004) | 0.430 |  |  |  |
| Fani, L.-2021 [78] | IL16 | 0.986 (0.926-1.051) | 0.675 |  |  |  |
| Meta-analysis | IL17 | 0.994 (0.898-1.100) | 0.586 | 0.007 | 1 | 0.000 |
| Yeung, C. H. C.-2021 [85] | IL17 | 0.994 (0.978-1.009) | 0.430 |  |  |  |
| Fani, L.-2021 [78] | IL17 | 0.982 (0.743-1.300) | 0.902 |  |  |  |
| Meta-analysis | IL18 | 1.003 (0.988-1.019) | 0.440 | 0.656 | 2 | 0.000 |
| Yeung, C. H. C.-2021 [85] | IL18 | 1.003 (0.996-1.010) | 0.420 |  |  |  |
| Fani, L.-2021 [78] | IL18 | 1.025 (0.973-1.080) | 0.353 |  |  |  |
| Tsui, A.-2018 [88] | IL18 | 0.960 (0.180-5.100) | 0.820 |  |  |  |
| Meta-analysis | CRP | 1.018 (1.005-1.031) | 0.016 | 7.737 | 6 | 0.224 |
| Zhang, Z.-2022 [89] | CRP | 1.020 (1.010-1.040) | 0.008 |  |  |  |
| Zhang, Z.-2022 [89] | CRP | 1.012 (0.997-1.028) | 0.114 |  |  |  |
| Wang, H.-2021 [7] | CRP (male) | 0.985 (0.743-1.306) | 0.919 |  |  |  |
| Wang, H.-2021 [7] | CRP (female) | 1.259 (1.005-1.577) | 0.045 |  |  |  |
| Handy, A.-2021 [87] | CRP | 1.000 (0.930-1.070) | 0.973 |  |  |  |
| Fani, L.-2021 [78] | CRP | 1.106 (1.010-1.210) | 0.029 |  |  |  |
| Larsson, S. C.-2017 [4] | CRP | 1.040 (0.940-1.170) | 0.440 |  |  |  |
| Meta-analysis | TNF-a | 1.007 (0.909-1.115) | 0.559 | 0.341 | 1 | 0.000 |
| Yeung, C. H. C.-2021 [85] | TNFa | 1.006 (0.990-1.022) | 0.440 |  |  |  |
| Andrews, S. J.-2019 [48] | serum TNF-a levels | 1.040 (0.930-1.160) | 0.470 |  |  |  |
| Meta-analysis | TNF-b | 0.998 (0.982-1.015) | 0.697 | 2.619 | 2 | 0.236 |
| Yeung, C. H. C.-2021 [85] | TNF-b | 0.996 (0.988-1.003) | 0.270 |  |  |  |
| Fani, L.-2021 [78] | TNF-β | 0.960 (0.896-1.028) | 0.241 |  |  |  |
| Yeung, C. H. C.-2021 [85] | Lymphotoxin-alpha /TNF-b | 1.005 (0.992-1.018) | 0.460 |  |  |  |
| Meta-analysis | TNF | 1.002 (0.734-1.368) | 0.956 | 1.635 | 1 | 0.389 |
| Yeung, C. H. C.-2021 [85] | TNF | 0.989 (0.973-1.005) | 0.180 |  |  |  |
| Andrews, S. J.-2019 [48] | Blood TNF expression | 1.050 (0.960-1.150) | 0.300 |  |  |  |
| Meta-analysis | TNF-related apoptosis-inducing ligand | 1.007 (0.836-1.212) | 0.724 | 2.459 | 1 | 0.593 |
| Yeung, C. H. C.-2021 [85] | TNF-related apoptosis-inducing ligand | 0.997 (0.990-1.004) | 0.390 |  |  |  |
| Fani, L.-2021 [78] | TNF-related apoptosis-inducing ligand | 1.029 (0.990-1.070) | 0.143 |  |  |  |
| Meta-analysis | MIF | 0.999 (0.977-1.021) | 0.818 | 0.069 | 2 | 0.000 |
| Yeung, C. H. C.-2021 [85] | MIF | 0.999 (0.986-1.013) | 0.920 |  |  |  |
| Yeung, C. H. C.-2021 [85] | MIF | 0.998 (0.983-1.014) | 0.830 |  |  |  |
| Fani, L.-2021 [78] | MIF | 1.022 (0.850-1.231) | 0.812 |  |  |  |
| Yeung, C. H. C.-2021 [85] | IFNG | 1.000 (0.984-1.016) | 0.990 |  |  |  |
| Yeung, C. H. C.-2021 [85] | IFNG (IFNG.2989.17.2) | 0.996 (0.985-1.007) | 0.510 |  |  |  |
| Yeung, C. H. C.-2021 [85] | lFNG (IFNG.14147.50.3) | 1.000 (0.987-1.012) | 0.940 |  |  |  |
| **Biomarkers of immunity and inflammation** |  |  |  |  |  |  |
| Meta-analysis | Complement C3 | 1.692 (0.101-28.283) | 0.254 | 0.063 | 1 | 0.000 |
| Rasmussen, K. L.-2018 [90] | Complement C3 | 1.660 (1.050-2.630) | 0.030 |  |  |  |
| Rasmussen, K. L.-2018 [90] | Complement C3 | 1.990 (0.520-7.650) | 0.320 |  |  |  |
| Yang, C.-2021 [91] | Complement C4 (Brain) | 2.273 (1.560-3.312) | <0.001 |  |  |  |
| Fani, L.-2021 [78] | Intercellular adhesion molecule 1: CD54 | 1.002 (0.430-2.340) | 0.996 |  |  |  |
| Tsui, A.-2018 [88] | Erythrocyte sedimentation rate | 1.370 (1.010-1.850) | 0.050 |  |  |  |
| Fani, L.-2021 [78] | Platelet count | 1.005 (0.934-1.080) | 0.895 |  |  |  |
| Fani, L.-2021 [78] | Basophil count | 1.171 (0.936-1.467) | 0.167 |  |  |  |
| Fani, L.-2021 [78] | Monocyte count | 0.968 (0.894-1.046) | 0.406 |  |  |  |
| Fani, L.-2021 [78] | Lymphocyte count | 1.035 (0.932-1.148) | 0.521 |  |  |  |
| Fani, L.-2021 [78] | CD4 count | 1.32 (1.126-1.514) | 0.005 |  |  |  |
| Fani, L.-2021 [78] | CD4 : CD8 lymphocyte ratio | 1.043 (0.912-1.192) | 0.539 |  |  |  |
| Meta-analysis | CD33 | 0.727 (0.197-2.687) | 0.535 | 84.842 | 4 | 0.953 |
| Png, G.-2021 [92] | CD33 (Serum) | 1.058 (1.030-1.087) | <0.001 |  |  |  |
| Png, G.-2021 [92] | CD33 (Serum) | 1.046 (1.024-1.069) | <0.001 |  |  |  |
| Png, G.-2021 [92] | Myeloid cell surface antigen CD33 (Serum) | 1.009 (1.006-1.013) | <0.001 |  |  |  |
| Yang, C.-2021 [91] | Myeloid cell surface antigen CD33 (CSF) | 0.093 (0.044-0.197) | <0.001 |  |  |  |
| Yang, C.-2021 [91] | Myeloid cell surface antigen CD33 (Plasma) | 1.535 (1.299-1.813) | <0.001 |  |  |  |
| **Omics traits** |  |  |  |  |  |  |
| ***Gut microbial metabolites*** |  |  |  |  |  |  |
| Meta-analysis | Trimethylamine-N-oxide | 0.990 (0.706-1.387) | 0.770 | 0.000 | 1 | 0.000 |
| Zhuang, Z.-2021 [93] | Trimethylamine-N-oxide | 0.990 (0.890-1.090) | 0.775 |  |  |  |
| Zhuang, Z.-2020 [94] | Trimethylamine-N-oxide | 0.990 (0.930-1.050) | 0.697 |  |  |  |
| Meta-analysis | Choline | 1.016 (0.714-1.447) | 0.665 | 0.029 | 1 | 0.000 |
| Zhuang, Z.-2021 [93] | Choline | 1.010 (0.920-1.100) | 0.905 |  |  |  |
| Zhuang, Z.-2020 [94] | Choline | 1.020 (0.950-1.090) | 0.650 |  |  |  |
| Meta-analysis | Carnitine | 1.029 (0.811-1.305) | 0.369 | 0.505 | 1 | 0.000 |
| Zhuang, Z.-2021 [93] | Carnitine | 1.050 (0.980-1.120) | 0.178 |  |  |  |
| Zhuang, Z.-2020 [94] | Carnitine | 1.020 (0.980-1.070) | 0.310 |  |  |  |
| Zhuang, Z.-2020 [94] | β-hydroxybutyric acid | 0.970 (0.900-1.040) | 0.371 |  |  |  |
| Zhuang, Z.-2020 [94] | γ-aminobutyric acid | 0.960 (0.920-1.000) | 0.034 |  |  |  |
| Zhuang, Z.-2020 [94] | Propionic acid | 0.970 (0.910-1.040) | 0.407 |  |  |  |
| Zhuang, Z.-2020 [94] | Serotonin | 0.990 (0.930-1.060) | 0.760 |  |  |  |
| Zhuang, Z.-2021 [93] | Betaine | 1.060 (1.000-1.120) | 0.056 |  |  |  |
| ***Gut microbiota-Blautia*** |  |  |  |  |  |  |
| Zhuang, Z.-2020 [94] | Blautia | 0.880 (0.790-0.990) | 0.028 |  |  |  |
| ***Neuroimaging feature*** |  |  |  |  |  |  |
| Wu, B. S.-2021 [95] | Surface area: lateral orbitofrontal | 1.040 (1.010-1.080) | 0.022 |  |  |  |
| Wu, B. S.-2021 [95] | Surface area: temporal pole | 0.950 (0.900-0.997) | 0.040 |  |  |  |
| Wu, B. S.-2021 [95] | Surface area: supramarginal | 1.050 (1.010-1.090) | 0.008 |  |  |  |
| Wu, B. S.-2021 [95] | Surface area: lingual | 1.030 (1.004-1.060) | 0.024 |  |  |  |
| Wu, B. S.-2021 [95] | Thickness of cuneus | 0.930 (0.890-0.980) | 0.006 |  |  |  |
| Meta-analysis | Neuroimaging feature: susceptibility-weighted T1 imaging | 1.037 (0.224-4.805) | 0.816 | 0.095 | 1 | 0.000 |
| Wang, H.-2021 [7] | Neuroimaging feature: susceptibility-weighted T1 imaging (male) | 1.084 (0.749-1.571) | 0.669 |  |  |  |
| Wang, H.-2021 [7] | Neuroimaging feature: susceptibility-weighted T1 imaging (female) | 1.005 (0.739-1.367) | 0.973 |  |  |  |
| Meta-analysis | Neuroimaging feature: hippocampal volume | 1.238 (0.407-3.763) | 0.247 | 0.607 | 1 | 0.000 |
| Wang, H.-2021 [7] | Neuroimaging feature: hippocampal volume (male) | 1.351 (1.023-1.785) | 0.034 |  |  |  |
| Wang, H.-2021 [7] | Neuroimaging feature: hippocampal volume (female) | 1.174 (0.944-1.459) | 0.149 |  |  |  |
| ***Leukocyte telomere length*** |  |  |  |  |  |  |
| Meta-analysis | Leukocyte telomere length | 1.100 (1.005-1.204) | 0.041 | 17.737 | 6 | 0.662 |
| Yu, G.-2021 [96] | Leukocyte telomere length | 0.790 (0.670-0.930) | 0.004 |  |  |  |
| Scheller Madrid, A.-2020 [97] | Short telomeres | 0.930 (0.720-1.180) | > 0.050 |  |  |  |
| Scheller Madrid, A.-2020 [97] | Short telomeres | 1.220 (0.880-1.690) | > 0.050 |  |  |  |
| Scheller Madrid, A.-2020 [97] | Short telomeres | 1.100 (1.020-1.180) | > 0.050 |  |  |  |
| Zhan, Y.-2015 [98] | Telomere length Shortening | 1.360 (1.120-1.670) | 0.002 |  |  |  |
| Gao, K.-2019 [99] | Telomere length | 1.030 (1.010-1.050) | 0.001 |  |  |  |
| Guo, Y.-2019 [100] | Leukocyte telomere length | 0.950 (0.920-0.990) | 0.010 |  |  |  |
| ***Lipid-lowering drug targets*** |  |  |  |  |  |  |
| Meta-analysis | PCSK9 (encoding the target for PCSK9 inhibitors, eg, evolocumab and alirocumab) inhibition | 1.449 (0.521-4.031） | 0.136 | 0.739 | 1 | 0.000 |
| Williams, D. M.-2020 [75] | PCSK9 (encoding the target for PCSK9 inhibitors, eg, evolocumab and alirocumab) inhibition | 1.410 (1.190-1.670) | < 0.050 |  |  |  |
| Williams, D. M.-2020 [75] | PCSK9 (encoding the target for PCSK9 inhibitors, eg, evolocumab and alirocumab) inhibition | 1.730 (1.120-2.670) | < 0.050 |  |  |  |
| Meta-analysis | Lowering PCSK9 concentration | 1.361 (0.038-49.260) | 0.472 | 2.645 | 1 | 0.622 |
| Williams, D. M.-2020 [75] | Lowering PCSK9 concentration | 1.100 (0.850-1.430) | 0.470 |  |  |  |
| Williams, D. M.-2020 [75] | Lowering PCSK9 concentration | 1.980 (1.020-3.810) | 0.040 |  |  |  |
| Meta-analysis | HMGCR (encoding the target for statins) inhibition | 0.912 (0.084-9.951) | 0.711 | 0.165 | 1 | 0.000 |
| Williams, D. M.-2020 [75] | HMGCR (encoding the target for statins) inhibition | 0.940 (0.630-1.390) | > 0.050 |  |  |  |
| Williams, D. M.-2020 [75] | HMGCR (encoding the target for statins) inhibition | 0.750 (0.270-2.050) | > 0.050 |  |  |  |
| Meta-analysis | ApoB (encoding the target of mipomersen) antisense | 1.126 (0.340-3.730) | 0.428 | 0.125 | 1 | 0.000 |
| Williams, D. M.-2020 [75] | ApoB (encoding the target of mipomersen) antisense | 1.140 (0.930-1.380) | > 0.050 |  |  |  |
| Williams, D. M.-2020 [75] | ApoB (encoding the target of mipomersen) antisense | 1.030 (0.610-1.750) | > 0.050 |  |  |  |
| Meta-analysis | NPC1L1 (encoding the target for ezetimibe) blockade | 1.171 (0.056-24.429) | 0.628 | 0.993 | 1 | 0.000 |
| Williams, D. M.-2020 [75] | NPC1L1 (encoding the target for ezetimibe) blockade | 1.300 (0.780-2.170) | > 0.050 |  |  |  |
| Williams, D. M.-2020 [75] | NPC1L1 (encoding the target for ezetimibe) blockade | 0.680 (0.210-2.170) | > 0.050 |  |  |  |
| ***Anti-obesity drug target genes*** |  |  |  |  |  |  |
| Zhuang, Q. S.-2021 [39] | Anti-obesity drug target gene: *ADRA1A* | 1.120 (0.940-1.320) | 0.190 |  |  |  |
| Zhuang, Q. S.-2021 [39] | Anti-obesity drug target gene: *ADRA1B* | 1.060 (0.980-1.150) | 0.130 |  |  |  |
| Zhuang, Q. S.-2021 [39] | Anti-obesity drug target gene: *CARTPT* | 1.020 (0.990-1.060) | 0.210 |  |  |  |
| Zhuang, Q. S.-2021 [39] | Anti-obesity drug target gene: *CNR1* | 0.950 (0.920-0.990) | 0.010 |  |  |  |
| Zhuang, Q. S.-2021 [39] | Anti-obesity drug target gene: *DRD2* | 0.980 (0.940-1.020) | 0.310 |  |  |  |
| Zhuang, Q. S.-2021 [39] | Anti-obesity drug target gene: *HRH1* | 0.950 (0.860-1.050) | 0.300 |  |  |  |
| Zhuang, Q. S.-2021 [39] | Anti-obesity drug target gene: *PNLIP* | 1.020 (0.990-1.060) | 0.240 |  |  |  |
| Zhuang, Q. S.-2021 [39] | Anti-obesity drug target gene: *THRB* | 0.990 (0.940-1.040) | 0.600 |  |  |  |
| Zhuang, Q. S.-2021 [39] | Anti-obesity drug target gene: *NPY* | 0.980 (0.940-1.030) | 0.490 |  |  |  |
| ***Common antihypertensive drug classes*** |  |  |  |  |  |  |
| Meta-analysis | Antihypertensive medications | 0.970 (0.586-1.607) | 0.588 | 1.122 | 1 | 0.109 |
| Ou, Y. N.-2021 [25] | Antihypertensive medications | 0.961 (0.944-0.978) | <0.001 |  |  |  |
| Walker, V. M.-2020 [28] | Antihypertensive drugs | 1.140 (0.830-1.560) | 0.410 |  |  |  |
| Meta-analysis | Beta-adrenoceptor blockers | 0.946 (0.349-2.561) | 0.608 | 0.350 | 1 | 0.000 |
| Ou, Y. N.-2021 [25] | β-blockers | 0.934 (0.786-1.081) | 0.362 |  |  |  |
| Walker, V. M.-2020 [28] | Beta-adrenoceptor blockers | 1.120 (0.630-2.010) | 0.690 |  |  |  |
| Meta-analysis | Calcium channel blockers | 1.134 (0.066-19.540) | 0.675 | 3.528 | 1 | 0.717 |
| Ou, Y. N.-2021 [25] | Calcium channel blocker | 0.959 (0.941-0.977) | <0.001 |  |  |  |
| Walker, V. M.-2020 [28] | Calcium channel blockers | 1.530 (0.940-2.490) | 0.090 |  |  |  |
| Meta-analysis | Thiazides | 0.912 (0.055-15.024) | 0.749 | 1.496 | 1 | 0.332 |
| Ou, Y. N.-2021 [25] | Thiazides | 1.008 (0.939-1.078) | 0.812 |  |  |  |
| Walker, V. M.-2020 [28] | Thiazides and related diuretics | 0.560 (0.220-1.440) | 0.230 |  |  |  |
| Ou, Y. N.-2021 [25] | Angiotensin receptor blockers | 0.940 (0.773-1.108) | 0.473 |  |  |  |
| Walker, V. M.-2020 [28] | Angiotensin-ll receptor antagonists | 0.570 (0.110-2.840) | 0.490 |  |  |  |
| Walker, V. M.-2020 [28] | Centrally acting antihypertensives | 1.120 (0.420-2.960) | 0.820 |  |  |  |
| Walker, V. M.-2020 [28] | Loop diuretics | 0.780 (0.180-3.400) | 0.740 |  |  |  |
| Walker, V. M.-2020 [28] | PSDs and aldosterone antagonists | 0.170 (0.020-1.330) | 0.090 |  |  |  |
| Walker, V. M.-2020 [28] | Renin inhibitors | 1.850 (0.150-23.500) | 0.630 |  |  |  |
| Walker, V. M.-2020 [28] | Adrenergic neurone blockers | 0.940 (0.050-16.170) | 0.970 |  |  |  |
| Walker, V. M.-2020 [28] | Alpha-adrenoceptor blockers | 1.100 (0.560-2.160) | 0.780 |  |  |  |
| Walker, V. M.-2020 [28] | Angintensin converting enzyme inhibitors | 13.200 (2.140-81.240) | 0.005 |  |  |  |
| Walker, V. M.-2020 [28] | Vasodilator antihypertensives | 0.980 (0.300-3.140) | 0.970 |  |  |  |
| ***Genome*** |  |  |  |  |  |  |
| Baird, D. A.-2021 [101] | *CCDC6* | 1.249 (1.157-1.319) | <0.001 |  |  |  |
| Baird, D. A.-2021 [101] | *TSPAN14* | 1.140 (1.085-1.193) | <0.001 |  |  |  |
| Baird, D. A.-2021 [101] | *KAT8* | 0.875 (0.837-0.941) | <0.001 |  |  |  |
| Baird, D. A.-2021 [101] | *ZNF646* | 0.806 (0.749-0.855) | <0.001 |  |  |  |
| Baird, D. A.-2021 [101] | *CCNT2-AS1* | 0.860 (0.811-0.898) | <0.001 |  |  |  |
| Baird, D. A.-2021 [101] | *PRSS36* | 0.874 (0.837-0.914) | <0.001 |  |  |  |
| Baird, D. A.-2021 [101] | *AC012146.1* | 0.866 (0.828-0.887) | <0.001 |  |  |  |
| Wingo, A. P.-2021 [102] | *EPHX2* | 1.063 (1.037-1.088) | <0.001 |  |  |  |
| Wingo, A. P.-2021 [102] | *PVR* | 0.592 (0.468-0.750) | <0.001 |  |  |  |
| Wingo, A. P.-2021 [102] | *SNX32* | 0.903 (0.861-0.947) | <0.001 |  |  |  |
| Wingo, A. P.-2021 [102] | *CTSH* | 1.050 (1.026-1.075) | <0.001 |  |  |  |
| Wingo, A. P.-2021 [102] | *RTFDC1* | 1.140 (1.070-1.214) | <0.001 |  |  |  |
| Wingo, A. P.-2021 [102] | *LACTB* | 1.098 (1.043-1.155) | <0.001 |  |  |  |
| Wingo, A. P.-2021 [102] | *ICA1L* | 0.816 (0.729-0.913) | <0.001 |  |  |  |
| Wingo, A. P.-2021 [102] | *DOC2A* | 0.713 (0.581-0.875) | 0.001 |  |  |  |
| Wingo, A. P.-2021 [102] | *PLEKHA1* | 1.484 (1.144-1.927) | 0.003 |  |  |  |
| Meta-analysis | ACE | 0.870 (0.659-1.148) | 0.099 | 0.817 | 1 | 0.000 |
| Baird, D. A.-2021 [101] | *ACE* | 0.858 (0.812-0.901) | <0.001 |  |  |  |
| Wingo, A. P.-2021 [102] | *ACE* | 0.895 (0.830-0.965) | 0.004 |  |  |  |
| Wingo, A. P.-2021 [102] | *STX4* | 1.593 (1.155-2.198) | 0.005 |  |  |  |
| Wingo, A. P.-2021 [102] | *STX6* | 1.372 (1.080-1.742) | 0.010 |  |  |  |
| Wingo, A. P.-2021 [102] | *CARHSP1* | 1.136 (1.028-1.256) | 0.012 |  |  |  |
| Zhu, J.-2021 [103] | *B4GALT3* (Blood) | 0.911 (0.883-0.940) | <0.001 |  |  |  |
| Zhu, J.-2021 [103] | *NDUFS2* (Blood) | 0.948 (0.930-0.967) | <0.001 |  |  |  |
| Zhu, J.-2021 [103] | *BIN1* (Blood) | 1.043 (1.027-1.059) | <0.001 |  |  |  |
| Zhu, J.-2021 [103] | *HLA-DRA* (Blood) | 1.174 (1.102-1.249) | <0.001 |  |  |  |
| Zhu, J.-2021 [103] | *CASTOR3* (Blood) | 0.892 (0.856-0.930) | <0.001 |  |  |  |
| Zhu, J.-2021 [103] | *EPHA1-AS1* (Blood) | 0.969 (0.960-0.979) | <0.001 |  |  |  |
| Zhu, J.-2021 [103] | *SLC24A4* (Blood) | 0.979 (0.972-0.987) | <0.001 |  |  |  |
| Zhu, J.-2021 [103] | *RIN3* (Blood) | 0.938 (0.914-0.962) | <0.001 |  |  |  |
| Zhu, J.-2021 [103] | *APH1B* (Blood) | 1.025 (1.015-1.035) | <0.001 |  |  |  |
| Meta-analysis | AC012146.7 | 0.976 (0.938-1.015) | 0.079 | 0.026 | 1 | 0.000 |
| Zhu, J.-2021 [103] | *AC012146.7* (Blood) | 0.976 (0.969-0.984) | <0.001 |  |  |  |
| Zhu, J.-2021 [103] | *AC012146.7* (Brain) | 0.975 (0.966-0.985) | <0.001 |  |  |  |
| Zhu, J.-2021 [103] | *ZNF232* (Blood) | 0.944 (0.924-0.964) | <0.001 |  |  |  |
| Zhu, J.-2021 [103] | *SIGLEC22P* (Blood) | 0.931 (0.905-0.956) | <0.001 |  |  |  |
| Zhu, J.-2021 [103] | *CD33* (Blood) | 1.038 (1.024-1.052) | <0.001 |  |  |  |
| Zhu, J.-2021 [103] | *CASS4* (Blood) | 1.172 (1.101-1.248) | <0.001 |  |  |  |
| Zhu, J.-2021 [103] | *RPL39P* (Blood) | 1.112 (1.069-1.156) | <0.001 |  |  |  |
| Meta-analysis | CR1 | 1.168 (0.268-5.092) | 0.407 | 45.797 | 1 | 0.978 |
| Baird, D. A.-2021 [101] | *CR1* | 1.315 (1.245-1.420) | <0.001 |  |  |  |
| Zhu, J.-2021 [103] | *CR1* (Brain) | 1.043 (1.029-1.057) | <0.001 |  |  |  |
| ***DNA methylation*** |  |  |  |  |  |  |
| Liu, D.-2021 [104] | cg05656486 (*NDUFS2*) (Brain) | 1.043 (1.025-1.061) | <0.001 |  |  |  |
| Liu, D.-2021 [104] | cg08850169 (*NDUFS2*) (Brain) | 1.047 (1.027-1.068) | <0.001 |  |  |  |
| Liu, D.-2021 [104] | cg16673712 (*NDUFS2*) (Brain) | 0.979 (0.970-0.989) | <0.001 |  |  |  |
| Liu, D.-2021 [104] | cg23274951 (*NDUFS2*) (Brain) | 1.045 (1.025-1.066) | <0.001 |  |  |  |
| Liu, D.-2021 [104] | cg24049880 (*NDUFS2*) (Brain) | 1.041 (1.025-1.057) | <0.001 |  |  |  |
| Liu, D.-2021 [104] | cg13210467 (*STAG3*) (Brain) | 0.957 (0.940-0.974) | <0.001 |  |  |  |
| Liu, D.-2021 [104] | cg22906224 (*STAG3*) (Brain) | 0.990 (0.986-0.994) | <0.001 |  |  |  |
| Liu, D.-2021 [104] | cg03887787 (*FIBP*) (Brain) | 0.980 (0.971-0.990) | <0.001 |  |  |  |
| Liu, D.-2021 [104] | cg19792802 (*FIBP*) (Brain) | 0.991 (0.987-0.995) | <0.001 |  |  |  |
| Liu, D.-2021 [104] | cg02220965 (*KAT8*) (Brain) | 1.018 (1.010-1.026) | <0.001 |  |  |  |
| Liu, D.-2021 [104] | cg04275947 (*KAT8*) (Brain) | 1.045 (1.023-1.068) | <0.001 |  |  |  |
| Liu, D.-2021 [104] | cg07078430 (*KAT8*) (Brain) | 0.980 (0.973-0.988) | <0.001 |  |  |  |
| Liu, D.-2021 [104] | cg04275947 (*RNF40*) (Brain) | 1.045 (1.023-1.068) | <0.001 |  |  |  |
| Liu, D.-2021 [104] | cg07078430 (*RNF40*) (Brain) | 0.980 (0.973-0.988) | <0.001 |  |  |  |
| Liu, D.-2021 [104] | cg04275947 (*PRSS36*) (Brain) | 1.045 (1.023-1.068) | <0.001 |  |  |  |
| Liu, D.-2021 [104] | cg07078430 (*PRSS36*) (Brain) | 0.980 (0.973-0.988) | <0.001 |  |  |  |
| Liu, D.-2021 [104] | cg02220965 (*C16orf93*) (Brain) | 1.018 (1.010-1.026) | <0.001 |  |  |  |
| Liu, D.-2021 [104] | cg04275947 (*C16orf93*) (Brain) | 1.045 (1.023-1.068) | <0.001 |  |  |  |
| Liu, D.-2021 [104] | cg03433048 (*ZNF232*) (Brain) | 1.017 (1.009-1.025) | <0.001 |  |  |  |
| Liu, D.-2021 [104] | cg20814095 (*ZNF232*) (Brain) | 1.021 (1.011-1.031) | <0.001 |  |  |  |
| Liu, D.-2021 [104] | cg03433048 (*AC012146.7*) (Brain) | 1.017 (1.009-1.025) | <0.001 |  |  |  |
| Liu, D.-2021 [104] | cg20814095 (*AC012146.7*) (Brain) | 1.021 (1.011-1.031) | <0.001 |  |  |  |
| Liu, D.-2021 [104] | cg09070378 (*FCER1G*) (Blood) | 1.027 (1.015-1.040) | <0.001 |  |  |  |
| Liu, D.-2021 [104] | cg19116668 (*PILRA*) (Blood) | 1.019 (1.014-1.024) | <0.001 |  |  |  |
| Liu, D.-2021 [104] | cg01669108 (*FIBP*) (Blood) | 0.986 (0.980-0.993) | <0.001 |  |  |  |
| Liu, D.-2021 [104] | cg23483894 (*FIBP*) (Blood) | 0.973 (0.960-0.986) | <0.001 |  |  |  |
| Liu, D.-2021 [104] | cg03887787 (*CTSW*) (Blood) | 0.991 (0.988-0.995) | <0.001 |  |  |  |
| Liu, D.-2021 [104] | cg01669108 (*CTSW*) (Blood) | 0.986 (0.980-0.993) | <0.001 |  |  |  |
| Liu, D.-2021 [104] | cg23483894 (*CTSW*) (Blood) | 0.973 (0.960-0.986) | <0.001 |  |  |  |
| Liu, D.-2021 [104] | cg17207590 (*APH1B*) (Blood) | 0.966 (0.950-0.982) | <0.001 |  |  |  |
| Liu, D.-2021 [104] | cg26675395 (*BCKDK*) (Blood) | 1.055 (1.029-1.082) | <0.001 |  |  |  |
| Liu, D.-2021 [104] | cg00249205 (*BCKDK*) (Blood) | 0.972 (0.960-0.985) | <0.001 |  |  |  |
| Liu, D.-2021 [104] | cg26949037 (*BCKDK*) (Blood) | 0.960 (0.941-0.980) | <0.001 |  |  |  |
| Liu, D.-2021 [104] | cg05768032 (*BCKDK*) (Blood) | 0.990 (0.985-0.994) | <0.001 |  |  |  |
| Liu, D.-2021 [104] | cg10421029 (*BCKDK*) (Blood) | 1.033 (1.018-1.048) | <0.001 |  |  |  |
| Liu, D.-2021 [104] | cg02220965 (*BCKDK*) (Blood) | 1.016 (1.009-1.022) | <0.001 |  |  |  |
| Liu, D.-2021 [104] | cg03418659 (*BCKDK*) (Blood) | 1.016 (1.009-1.023) | <0.001 |  |  |  |
| Liu, D.-2021 [104] | cg01067137 (*BCKDK*) (Blood) | 1.017 (1.009-1.026) | <0.001 |  |  |  |
| ***Proteome*** |  |  |  |  |  |  |
| Meta-analysis | Vitamin D-binding protein | 0.840 (0.031-22.478) | 0.623 | 8.577 | 1 | 0.883 |
| Handy, A.-2021 [87] | Vitamin D-binding protein | 1.060 (0.990-1.140) | 0.091 |  |  |  |
| Zhang, H.-2020 [105] | Vitamin D-binding protein | 0.630 (0.450-0.890) | 0.009 |  |  |  |
| Handy, A.-2021 [87] | APOE ε3 | 0.990 (0.920-1.060) | 0.734 |  |  |  |
| Handy, A.-2021 [87] | Apolipoprotein B-100 | 1.030 (0.950-1.110) | 0.518 |  |  |  |
| Yang, C.-2021 [91] | Tyrosine-protein phosphatase nonreceptor type 1 (CSF) | 0.003 (3.81E-04-0.023) | <0.001 |  |  |  |
| Yang, C.-2021 [91] | SLAM family member 5 (CSF) | 0.091 (0.044-0.189) | <0.001 |  |  |  |
| Yang, C.-2021 [91] | Endothelial monocyte-activating polypeptide 2 (Plasma) | 0.043 (0.027-0.069) | <0.001 |  |  |  |
| Yang, C.-2021 [91] | SPARC-like protein 1 (Plasma) | 20.934 (6.176-70.954) | <0.001 |  |  |  |
| Yang, C.-2021 [91] | Hemopexin (Plasma) | 2.288 (1.652-3.169) | <0.001 |  |  |  |
| Yang, C.-2021 [91] | Sialic acid-binding Ig-like lectin 14 (Plasma) | 0.227 (0.132-0.390) | <0.001 |  |  |  |
| Yang, C.-2021 [91] | Sialic acid-binding Ig-like lectin 6 (Plasma) | 0.152 (0.065-0.355) | <0.001 |  |  |  |
| Yang, C.-2021 [91] | Junctional adhesion molecule-like (Plasma) | 1.939 (1.422-2.645) | <0.001 |  |  |  |
| Yang, C.-2021 [91] | cAMP-specific 3',5'-cyclic phosphodiesterase 4D (Plasma) | 43.143 (9.937-187.306) | <0.001 |  |  |  |
| Yang, C.-2021 [91] | Cathepsin F (Plasma) | 11.637 (3.632-37.290) | <0.001 |  |  |  |
| Yang, C.-2021 [91] | Kallikrein-13 (Plasma) | 0.102 (0.042-0.251) | <0.001 |  |  |  |
| Yang, C.-2021 [91] | Ephrin type-A receptor 5 (Plasma) | 0.154 (0.080-0.297) | <0.001 |  |  |  |
| Yang, C.-2021 [91] | Cyclin-dependent kinase inhibitor 1B (Plasma) | 0.202 (0.101-0.404) | <0.001 |  |  |  |
| Yang, C.-2021 [91] | Ficolin-2 (Plasma) | 0.163 (0.070-0.381) | <0.001 |  |  |  |
| Yang, C.-2021 [91] | Cytokine receptor-like factor 1:Cardiotrophin-like cytokine factor 1 Complex (Brain) | 0.003 (2.12E-04-0.048) | <0.001 |  |  |  |
| Yang, C.-2021 [91] | Copine-1 (Brain) | 0.440 (0.296-0.654) | <0.001 |  |  |  |
| Yang, C.-2021 [91] | Leucine-rich repeat transmembrane neuronal protein 1 (Brain) | 0.007 (0.001-0.058) | <0.001 |  |  |  |
| Yang, C.-2021 [91] | A disintegrin and metalloproteinase with thrombospondin motifs 4 (Brain) | 197773.744 (11653.427-3356476.468) | <0.001 |  |  |  |
| Yang, C.-2021 [91] | Ras GTPase-activating protein 1 (Brain) | 187.888 (24.381-1447.932) | <0.001 |  |  |  |
| Yang, C.-2021 [91] | Scavenger receptor cysteine-rich type 1 protein M130 (Brain) | 0.021 (0.004-0.114) | <0.001 |  |  |  |
| Meta-analysis | APP | 0.986 (0.732-1.327) | 0.649 | 1.098 | 1 | 0.089 |
| Yeung, C. H. C.-2021 [106] | Amyloid precursor protein (APP) | 0.970 (0.920-1.020) | 0.195 |  |  |  |
| Yeung, C. H. C.-2021 [106] | APP | 1.020 (0.940-1.100) | 0.642 |  |  |  |
| Meta-analysis | APLP2 | 0.985 (0.729-1.332) | 0.646 | 0.412 | 1 | 0.000 |
| Yeung, C. H. C.-2021 [106] | APLP2 | 0.970 (0.910-1.040) | 0.440 |  |  |  |
| Yeung, C. H. C.-2021 [106] | Amyloid-like protein 2 (APLP2) | 1.000 (0.940-1.070) | 0.925 |  |  |  |
| Meta-analysis | SAP | 1.015 (0.775-1.330) | 0.609 | 0.054 | 1 | 0.000 |
| Yeung, C. H. C.-2021 [106] | Serum amyloid P-component (SAP) | 1.010 (0.950-1.070) | 0.773 |  |  |  |
| Yeung, C. H. C.-2021 [106] | SAP | 1.020 (0.970-1.090) | 0.421 |  |  |  |
| Meta-analysis | Aβ_1–40_ | 1.017 (0.583-1.775) | 0.762 | 0.047 | 1 | 0.000 |
| Yeung, C. H. C.-2021 [106] | Amyloid beta (Aβ)_1–40_ | 1.030 (0.890-1.180) | 0.732 |  |  |  |
| Yeung, C. H. C.-2021 [106] | Aβ_1–40_ | 1.010 (0.910-1.130) | 0.804 |  |  |  |
| Meta-analysis | Aβ_1–42_ peptides | 0.950 (0.451-1.996) | 0.535 | 0.198 | 1 | 0.000 |
| Yeung, C. H. C.-2021 [106] | Aβ_1–42_ peptides | 0.920 (0.770-1.100) | 0.370 |  |  |  |
| Yeung, C. H. C.-2021 [106] | Aβ_1–42_ peptides | 0.970 (0.830-1.120) | 0.652 |  |  |  |
| Meta-analysis | Aβ_42_ | 0.619 (0.0217-17.658) | 0.320 | 0.462 | 1 | 0.000 |
| Yeung, C. H. C.-2021 [106] | Aβ_42_ | 0.690 (0.380-1.270) | 0.233 |  |  |  |
| Yeung, C. H. C.-2021 [106] | Aβ_42_ | 0.460 (0.170-1.260) | 0.130 |  |  |  |
| Meta-analysis | Total tau | 1.143 (0.326-4.005 | 0.405 | 0.283 | 1 | 0.000 |
| Yeung, C. H. C.-2021 [106] | Total tau | 1.080 (0.810-1.430) | 0.598 |  |  |  |
| Yeung, C. H. C.-2021 [106] | Total tau | 1.200 (0.920-1.560) | 0.183 |  |  |  |
| Meta-analysis | Phosphorylated tau_181_ | 1.072 (0.205-5.612) | 0.687 | 1.890 | 1 | 0.471 |
| Yeung, C. H. C.-2021 [106] | Phosphorylated tau_181_ | 0.960 (0.770-1.200) | 0.738 |  |  |  |
| Yeung, C. H. C.-2021 [106] | Phosphorylated tau_181_ | 1.250 (0.920-1.690) | 0.151 |  |  |  |
| When a noun in the table appears three or more times, the abbreviation is used to indicate it, as follows: APOE, Apolipoprotein E; BMI, Body mass index; CSF, cerebrospinal fluid; CVD, cardiovascular disease; CAD, coronary artery disease; CRP, C-reactive protein; CTACK, cutaneous T-cell attracting chemokine; DBP, diastolic blood pressure; FEV1, forced expiratory volume in 1 second; FGFBasic, basic fibroblast growth factor; FT4, free thyroxine; FVC, forced vital capacity; GRO, growth-regulated oncogene; HbA1c, glycosylated hemoglobin A1c; HDL, high-density lipoprotein; HDL-C, HDL-cholesterol; HGF, hepatocyte growth factor; HSV, herpes simplex virus; IFNG, interferon gamma; IGF1, insulin-like growth factor 1; IOP+, Institute of Psychiatry Plus; IP-10, interferon-gamma-induced protein 10; IL, interleukin; IR, insulin resistance; LDL-C, low-density lipoprotein cholesterol; Lp(a), lipoprotein(a); L5Timing, least active 5 hours [L5] timing; MIF, macrophage-migration inhibitory factor; PD, Parkinson’s disease; PDGFB/PDGFbb, platelet-derived growth factor subunit B; RA, rheumatoid arthritis; SBP, systolic blood pressure; SCF, stem cell factor; SCGFb, stem cell growth factor beta; TC, total cholesterol; TG, triglycerides; TNF, tumor necrosis factor; TSH, thyrotropin; T2DM, type 2 diabetes mellitus; WHR, Waist-to-hip ratio; WHRadjBMI, Waist-to-hip ratio adjusted by body mass index; 25(OH)D, 25-hydroxyvitamin D. | | | | | | |
|  |  |  |  |  |  |  |
| **References** |  |  |  |  |  |  |
| 1. Wang Z, Meng L, Shen L, et al. Impact of modifiable risk factors on Alzheimer's disease: A two-sample Mendelian randomization study. *Neurobiology of aging* 2020;91:167.e11-67.e19. doi: 10.1016/j.neurobiolaging.2020.02.018 | | | | | | |
| 2. Zhang Q, Xu F, Wang L, et al. Detecting potential causal relationship between multiple risk factors and Alzheimer's disease using multivariable Mendelian randomization. *Aging* 2020;12(21):21747‐57. doi: 10.18632/aging.103983 | | | | | | |
| 3. Østergaard SD, Mukherjee S, Sharp SJ, et al. Associations between Potentially Modifiable Risk Factors and Alzheimer Disease: A Mendelian Randomization Study. *PLoS medicine* 2015;12(6):e1001841. doi: 10.1371/journal.pmed.1001841 [published Online First: 2015/06/17] | | | | | | |
| 4. Larsson SC, Traylor M, Malik R, et al. Modifiable pathways in Alzheimer's disease: Mendelian randomisation analysis. *BMJ (Clinical research ed)* 2017;359:j5375. doi: 10.1136/bmj.j5375 | | | | | | |
| 5. Andrews SJ, Fulton-Howard B, O'Reilly P, et al. Causal Associations Between Modifiable Risk Factors and the Alzheimer's Phenome. *Annals of neurology* 2021;89(1):54-65. doi: 10.1002/ana.25918 | | | | | | |
| 6. Anderson EL, Howe LD, Wade KH, et al. Education, intelligence and Alzheimer's disease: evidence from a multivariable two-sample Mendelian randomization study. *International journal of epidemiology* 2020;49(4):1163-72. doi: 10.1093/ije/dyz280 | | | | | | |
| 7. Wang H, Rosenthal BS, Makowski C, et al. Causal association of cognitive reserve on Alzheimer's disease with putative sex difference. *Alzheimer's and Dementia: Diagnosis, Assessment and Disease Monitoring* 2021;13(1):e12270. doi: 10.1002/dad2.12270 | | | | | | |
| 8. Ko H, Kim S, Kim K, et al. Genome-wide association study of occupational attainment as a proxy for cognitive reserve. *Brain : a journal of neurology* 2021;145(4):1436-48. doi: 10.1093/brain/awab351 | | | | | | |
| 9. Jansen IE, Savage JE, Watanabe K, et al. Genome-wide meta-analysis identifies new loci and functional pathways influencing Alzheimer's disease risk. *Nature genetics* 2019;51(3):404-13. doi: 10.1038/s41588-018-0311-9 | | | | | | |
| 10. Raghavan NS, Vardarajan B, Mayeux R. Genomic variation in educational attainment modifies Alzheimer disease risk. *Neurology: Genetics* 2019;5(2):e310. doi: 10.1212/NXG.0000000000000310 | | | | | | |
| 11. Li M, Lin J, Liang S, et al. The role of age at menarche and age at menopause in Alzheimer's disease: evidence from a bidirectional mendelian randomization study. *Aging* 2021;13(15):19722-49. doi: 10.18632/aging.203384 | | | | | | |
| 12. Huang J, Zuber V, Matthews PM, et al. Sleep, major depressive disorder, and Alzheimer disease: A Mendelian randomization study. *Neurology* 2020;95(14):e1963-e70. doi: 10.1212/WNL.0000000000010463 | | | | | | |
| 13. Anderson EL, Richmond RC, Jones SE, et al. Is disrupted sleep a risk factor for Alzheimer's disease? Evidence from a two-sample Mendelian randomization analysis. *International journal of epidemiology* 2021;50(3):817-28. doi: 10.1093/ije/dyaa183 | | | | | | |
| 14. Henry A, Katsoulis M, Masi S, et al. The relationship between sleep duration, cognition and dementia: a Mendelian randomization study. *International journal of epidemiology* 2019;48(3):849-60. doi: 10.1093/ije/dyz071 | | | | | | |
| 15. Cullell N, Carcel-Marquez J, Gallego-Fabrega C, et al. Sleep/wake cycle alterations as a cause of neurodegenerative diseases: a Mendelian randomization study. *Neurobiology of aging* 2021;106:320.e1-20.e12. doi: 10.1016/j.neurobiolaging.2021.05.008 | | | | | | |
| 16. Andrews SJ, Goate A, Anstey KJ. Association between alcohol consumption and Alzheimer's disease: A Mendelian randomization study. *Alzheimer's & dementia : the journal of the Alzheimer's Association* 2020;16(2):345-53. doi: 10.1016/j.jalz.2019.09.086 | | | | | | |
| 17. Yang YX, Kuo K, Li HQ, et al. Investigating Causal Relations Between Risk Tolerance, Risky Behaviors, and Alzheimer's Disease: A Bidirectional Two-Sample Mendelian Randomization Study. *Journal of Alzheimer's disease : JAD* 2020;78(4):1679-87. doi: 10.3233/jad-200773 [published Online First: 2020/11/14] | | | | | | |
| 18. Ma YH, Yang YX, Shen XN, et al. Evaluation relationships between subjective wellbeing, personality traits, and Alzheimer's disease: a two-sample Mendelian randomization study. *Journal of psychiatric research* 2021;137:498‐505. doi: 10.1016/j.jpsychires.2021.03.033 | | | | | | |
| 19. Shen LX, Yang YX, Kuo K, et al. Social Isolation, Social Interaction, and Alzheimer's Disease: a Mendelian Randomization Study. *Journal of Alzheimer's disease* 2021;80(2):665‐72. doi: 10.3233/JAD-201442 | | | | | | |
| 20. Baumeister SE, Karch A, Bahls M, et al. Physical activity and risk of Alzheimer disease: A 2-sample mendelian randomization study. *Neurology* 2020;95(13):e1897-e905. doi: 10.1212/wnl.0000000000010013 [published Online First: 2020/07/19] | | | | | | |
| 21. Wu PF, Lu H, Zhou X, et al. Assessment of causal effects of physical activity on neurodegenerative diseases: A Mendelian randomization study. *Journal of sport and health science* 2021;10(4):454-61. doi: 10.1016/j.jshs.2021.01.008 [published Online First: 2021/01/31] | | | | | | |
| 22. Yang F, Chen S, Qu Z, et al. Genetic Liability to Sedentary Behavior in Relation to Stroke, Its Subtypes and Neurodegenerative Diseases: A Mendelian Randomization Study. *Frontiers in Aging Neuroscience* 2021;13:757388. doi: 10.3389/fnagi.2021.757388 | | | | | | |
| 23. Li X, Tian Y, Yang YX, et al. Life Course Adiposity and Alzheimer's Disease: A Mendelian Randomization Study. *Journal of Alzheimer's disease : JAD* 2021;82(2):503-12. doi: 10.3233/jad-210345 [published Online First: 2021/06/01] | | | | | | |
| 24. Zhou Y, Sun X, Zhou M. Body Shape and Alzheimer’s Disease: A Mendelian Randomization Analysis. *Frontiers in Neuroscience* 2019;13:1084. doi: 10.3389/fnins.2019.01084 | | | | | | |
| 25. Ou YN, Yang YX, Shen XN, et al. Genetically determined blood pressure, antihypertensive medications, and risk of Alzheimer's disease: a Mendelian randomization study. *Alzheimer's research & therapy* 2021;13(1):41. doi: 10.1186/s13195-021-00782-y [published Online First: 2021/02/11] | | | | | | |
| 26. Zhu Z, Zheng Z, Zhang F, et al. Causal associations between risk factors and common diseases inferred from GWAS summary data. *Nature communications* 2018;9(1):224. doi: 10.1038/s41467-017-02317-2 | | | | | | |
| 27. Sproviero W, Winchester L, Newby D, et al. High Blood Pressure and Risk of Dementia: A Two-Sample Mendelian Randomization Study in the UK Biobank. *Biological psychiatry* 2021;89(8):817-24. doi: 10.1016/j.biopsych.2020.12.015 | | | | | | |
| 28. Walker VM, Kehoe PG, Martin RM, et al. Repurposing antihypertensive drugs for the prevention of Alzheimer's disease: a Mendelian randomization study. *International journal of epidemiology* 2020;49(4):1132-40. doi: 10.1093/ije/dyz155 | | | | | | |
| 29. Mukherjee S, Walter S, Kauwe JSK, et al. Genetically predicted body mass index and Alzheimer's disease-related phenotypes in three large samples: Mendelian randomization analyses. *Alzheimer's & dementia : the journal of the Alzheimer's Association* 2015;11(12):1439-51. doi: 10.1016/j.jalz.2015.05.015 | | | | | | |
| 30. Nordestgaard LT, Tybjærg-Hansen A, Nordestgaard BG, et al. Body Mass Index and Risk of Alzheimer's Disease: A Mendelian Randomization Study of 399,536 Individuals. *The Journal of clinical endocrinology and metabolism* 2017;102(7):2310-20. doi: 10.1210/jc.2017-00195 | | | | | | |
| 31. Li QS, Tian C, Hinds D, et al. The association of clinical phenotypes to known AD/FTD genetic risk loci and their inter-relationship. *PloS one* 2020;15(11):e0241552. doi: 10.1371/journal.pone.0241552 | | | | | | |
| 32. Grace C, Clarke R, Goel A, et al. Lack of genetic support for shared aetiology of Coronary Artery Disease and Late-onset Alzheimer's disease. *Scientific reports* 2018;8(1):7102. doi: 10.1038/s41598-018-25460-2 | | | | | | |
| 33. Wang T, Ni QB, Wang K, et al. Stroke and Alzheimer's Disease: A Mendelian Randomization Study. *Frontiers in genetics* 2020;11:581. doi: 10.3389/fgene.2020.00581 [published Online First: 2020/08/08] | | | | | | |
| 34. Kwok MK, Schooling CM. Mendelian randomization study on atrial fibrillation and cardiovascular disease subtypes. *Scientific reports* 2021;11(1):18682. doi: 10.1038/s41598-021-98058-w | | | | | | |
| 35. Han Z, Tian R, Ren P, et al. Parkinson's disease and Alzheimer's disease: a Mendelian randomization study. *BMC medical genetics* 2018;19(Suppl 1):215. doi: 10.1186/s12881-018-0721-7 [published Online First: 2019/01/02] | | | | | | |
| 36. Daghlas I, Rist PM, Chasman DI. Effect of genetic liability to migraine on cognition and brain volume: a Mendelian randomization study. *Cephalalgia* 2020;40(9):998-1002. doi: 10.1177/0333102420916751 | | | | | | |
| 37. Higbee D, Granell R, Walton E, et al. Examining the possible causal relationship between lung function, COPD and Alzheimer's disease: a Mendelian randomisation study. *BMJ open respiratory research* 2021;8(1):e000759. doi: 10.1136/bmjresp-2020-000759 | | | | | | |
| 38. Russ TC, Harris SE, Batty GD. Pulmonary Function and Risk of Alzheimer Dementia: Two-Sample Mendelian Randomization Study. *Chest* 2021;160(1):274-76. doi: 10.1016/j.chest.2020.11.056 [published Online First: 2020/12/16] | | | | | | |
| 39. Zhuang QS, Meng L, Wang Z, et al. Associations Between Obesity and Alzheimer's Disease: Multiple Bioinformatic Analyses. *J Alzheimers Dis* 2021;80(1):271-81. doi: 10.3233/jad-201235 [published Online First: 2021/02/02] | | | | | | |
| 40. Thomassen JQ, Tolstrup JS, Benn M, et al. Type-2 diabetes and risk of dementia: observational and Mendelian randomisation studies in 1 million individuals. *Epidemiology and psychiatric sciences* 2020;29:e118. doi: 10.1017/S2045796020000347 | | | | | | |
| 41. Pan Y, Chen W, Yan H, et al. Glycemic traits and Alzheimer's disease: a Mendelian randomization study. *Aging* 2020;12(22):22688-99. doi: 10.18632/aging.103887 | | | | | | |
| 42. Garfield V, Farmaki AE, Fatemifar G, et al. Relationship Between Glycemia and Cognitive Function, Structural Brain Outcomes, and Dementia: A Mendelian Randomization Study in the UK Biobank. *Diabetes* 2021;70(10):2313-21. doi: 10.2337/db20-0895 [published Online First: 2021/02/27] | | | | | | |
| 43. Zhou M, Li H, Wang Y, et al. Causal effect of insulin resistance on small vessel stroke and Alzheimer's disease: A Mendelian randomization analysis. *European Journal of Neurology* 2021;29(3)::698-706. doi: 10.1111/ene.15190 | | | | | | |
| 44. Walter S, Marden JR, Kubzansky LD, et al. Diabetic Phenotypes and Late-Life Dementia Risk: A Mechanism-specific Mendelian Randomization Study. *Alzheimer disease and associated disorders* 2016;30(1):15-20. doi: 10.1097/wad.0000000000000128 [published Online First: 2015/12/10] | | | | | | |
| 45. Benn M, Nordestgaard BG, Tybjærg-Hansen A, et al. Impact of glucose on risk of dementia: Mendelian randomisation studies in 115,875 individuals. *Diabetologia* 2020;63(6):1151-61. doi: 10.1007/s00125-020-05124-5 | | | | | | |
| 46. Seddighi S, Houck AL, Rowe JB, et al. Evidence of a Causal Association Between Cancer and Alzheimer's Disease: a Mendelian Randomization Analysis. *Scientific reports* 2019;9(1):13548. doi: 10.1038/s41598-019-49795-6 | | | | | | |
| 47. Policicchio S, Ahmad AN, Powell JF, et al. Rheumatoid arthritis and risk for Alzheimer's disease: a systematic review and meta-analysis and a Mendelian Randomization study. *Scientific reports* 2017;7(1):12861. doi: 10.1038/s41598-017-13168-8 | | | | | | |
| 48. Andrews SJ, Goate A. Mendelian randomization indicates that TNF is not causally associated with Alzheimer's disease. *Neurobiology of aging* 2019;84:241.e1-41.e3. doi: 10.1016/j.neurobiolaging.2019.09.003 | | | | | | |
| 49. Bae SC, Lee YH. Causal association between rheumatoid arthritis and a decreased risk of Alzheimer's disease : A Mendelian randomization study. *Zeitschrift fur Rheumatologie* 2019;78(4):359-64. doi: 10.1007/s00393-018-0504-8 | | | | | | |
| 50. Lee YH. Gout and the risk of Alzheimer's disease: A Mendelian randomization study. *International journal of rheumatic diseases* 2019;22(6):1046-51. doi: 10.1111/1756-185X.13548 | | | | | | |
| 51. Kwok MK, Schooling CM. Herpes simplex virus and Alzheimer's disease: a Mendelian randomization study. *Neurobiology of aging* 2021;99:101.e11-01.e13. doi: 10.1016/j.neurobiolaging.2020.09.025 | | | | | | |
| 52. Huang SY, Yang YX, Kuo K, et al. Herpesvirus infections and Alzheimer's disease: a Mendelian randomization study. *Alzheimer's research & therapy* 2021;13(1):158. doi: 10.1186/s13195-021-00905-5 [published Online First: 2021/09/26] | | | | | | |
| 53. Sun YQ, Richmond RC, Chen Y, et al. Mixed evidence for the relationship between periodontitis and Alzheimer's disease: A bidirectional Mendelian randomization study. *PLoS One* 2020;15(1):e0228206. doi: 10.1371/journal.pone.0228206 [published Online First: 2020/01/25] | | | | | | |
| 54. Zhang Z, Wang M, Yuan S, et al. Genetically predicted milk intake and risk of neurodegenerative diseases. *Nutrients* 2021;13(8):2893. doi: 10.3390/nu13082893 | | | | | | |
| 55. Zhang Z, Wang M, Yuan S, et al. Genetically Predicted Coffee Consumption and Risk of Alzheimer's Disease and Stroke. *Journal of Alzheimer's disease* 2021;83(4):1815-23. doi: 10.3233/JAD-210678 | | | | | | |
| 56. Kwok MK, Leung GM, Schooling CM. Habitual coffee consumption and risk of type 2 diabetes, ischemic heart disease, depression and Alzheimer's disease: a Mendelian randomization study. *Scientific reports* 2016;6:36500. doi: 10.1038/srep36500 | | | | | | |
| 57. Shi Y, Liu R, Guo Y, et al. An Updated Mendelian Randomization Analysis of the Association Between Serum Calcium Levels and the Risk of Alzheimer's Disease. *Frontiers in genetics* 2021;12:731391. doi: 10.3389/fgene.2021.731391 | | | | | | |
| 58. He Y, Zhang H, Wang T, et al. Impact of Serum Calcium Levels on Alzheimer's Disease: a Mendelian Randomization Study. *Journal of Alzheimer's disease* 2020;76(2):713‐24. doi: 10.3233/JAD-191249 | | | | | | |
| 59. Cheng WW, Zhu Q, Zhang HY. Mineral Nutrition and the Risk of Chronic Diseases: A Mendelian Randomization Study. *Nutrients* 2019;11(2):378. doi: 10.3390/nu11020378 [published Online First: 2019/02/15] | | | | | | |
| 60. Tomata Y, Larsson SC, Hägg S. Polyunsaturated fatty acids and risk of Alzheimer's disease: a Mendelian randomization study. *European journal of nutrition* 2020;59(4):1763-66. doi: 10.1007/s00394-019-02126-x | | | | | | |
| 61. Lord J, Jermy B, Green R, et al. Mendelian randomization identifies blood metabolites previously linked to midlife cognition as causal candidates in Alzheimer's disease. *Proceedings of the National Academy of Sciences of the United States of America* 2021;118(16):e2009808118. doi: 10.1073/pnas.2009808118 | | | | | | |
| 62. Roostaei T, Felsky D, Nazeri A, et al. Genetic influence of plasma homocysteine on Alzheimer's disease. *Neurobiology of aging* 2018;62:243.e7-43.e14. doi: 10.1016/j.neurobiolaging.2017.09.033 | | | | | | |
| 63. Adams CD. Circulating Glutamine and Alzheimer's Disease: A Mendelian Randomization Study. *Clinical interventions in aging* 2020;15:185-93. doi: 10.2147/CIA.S239350 | | | | | | |
| 64. Larsson SC, Markus HS. Branched-chain amino acids and Alzheimer's disease: a Mendelian randomization analysis. *Scientific reports* 2017;7(1):13604. doi: 10.1038/s41598-017-12931-1 | | | | | | |
| 65. Gagliano Taliun SA. Genetic determinants of low vitamin B12 levels in Alzheimer's disease risk. *Alzheimer's & dementia (Amsterdam, Netherlands)* 2019;11:430-34. doi: 10.1016/j.dadm.2019.04.007 [published Online First: 2019/06/18] | | | | | | |
| 66. Liu H, Zhang Y, Hu Y, et al. Mendelian randomization to evaluate the effect of plasma vitamin C levels on the risk of Alzheimer’s disease. *Genes and Nutrition* 2021;16(1):19. doi: 10.1186/s12263-021-00700-9 | | | | | | |
| 67. Chen L, Sun X, Wang Z, et al. The impact of plasma vitamin C levels on the risk of cardiovascular diseases and Alzheimer's disease: A Mendelian randomization study. *Clinical nutrition (Edinburgh, Scotland)* 2021;40(10):5327-34. doi: 10.1016/j.clnu.2021.08.020 | | | | | | |
| 68. Williams DM, Hägg S, Pedersen NL. Circulating antioxidants and Alzheimer disease prevention: a Mendelian randomization study. *The American journal of clinical nutrition* 2019;109(1):90-98. doi: 10.1093/ajcn/nqy225 | | | | | | |
| 69. Wang L, Qiao Y, Zhang H, et al. Circulating Vitamin D Levels and Alzheimer's Disease: A Mendelian Randomization Study in the IGAP and UK Biobank. *Journal of Alzheimer's disease : JAD* 2020;73(2):609-18. doi: 10.3233/JAD-190713 | | | | | | |
| 70. Larsson SC, Traylor M, Markus HS, et al. Serum Parathyroid Hormone, 25-Hydroxyvitamin D, and Risk of Alzheimer's Disease: A Mendelian Randomization Study. *Nutrients* 2018;10(9):1243. doi: 10.3390/nu10091243 | | | | | | |
| 71. Mokry LE, Ross S, Morris JA, et al. Genetically decreased vitamin D and risk of Alzheimer disease. *Neurology* 2016;87(24):2567-74. doi: 10.1212/WNL.0000000000003430 | | | | | | |
| 72. Liu G, Zhao Y, Jin S, et al. Circulating vitamin E levels and Alzheimer's disease: a Mendelian randomization study. *Neurobiology of aging* 2018;72:189.e1‐89.e9. doi: 10.1016/j.neurobiolaging.2018.08.008 | | | | | | |
| 73. Proitsi P, Lupton MK, Velayudhan L, et al. Genetic Predisposition to Increased Blood Cholesterol and Triglyceride Lipid Levels and Risk of Alzheimer Disease: A Mendelian Randomization Analysis. *PLoS medicine* 2014;11(9):e1001713. doi: 10.1371/journal.pmed.1001713 | | | | | | |
| 74. Benn M, Nordestgaard BG, Frikke-Schmidt R, et al. Low LDL cholesterol, PCSK9 and HMGCR genetic variation, and risk of Alzheimer's disease and Parkinson's disease: Mendelian randomisation study. *BMJ (Clinical research ed)* 2017;357:j1648. doi: 10.1136/bmj.j1648 | | | | | | |
| 75. Williams DM, Finan C, Schmidt AF, et al. Lipid lowering and Alzheimer disease risk: A mendelian randomization study. *Annals of neurology* 2020;87(1):30-39. doi: 10.1002/ana.25642 | | | | | | |
| 76. Dunk MM, Driscoll I. Total Cholesterol and APOE-Related Risk for Alzheimer's Disease in the Alzheimer's Disease Neuroimaging Initiative. *Journal of Alzheimer's disease : JAD* 2021;85(4):1519-28. doi: 10.3233/JAD-215091 | | | | | | |
| 77. Kunutsor SK, Laukkanen JA, Burgess S. Genetically elevated gamma-glutamyltransferase and Alzheimer's disease. *Experimental gerontology* 2018;106:61-66. doi: 10.1016/j.exger.2018.03.001 | | | | | | |
| 78. Fani L, Georgakis MK, Ikram MA, et al. Circulating biomarkers of immunity and inflammation, risk of Alzheimer's disease, and hippocampal volume: a Mendelian randomization study. *Translational psychiatry* 2021;11(1):291. doi: 10.1038/s41398-021-01400-z | | | | | | |
| 79. Li GH, Cheung CL, Cheung EY, et al. Genetically Determined TSH Level Within Reference Range Is Inversely Associated With Alzheimer Disease. *J Clin Endocrinol Metab* 2021;106(12):e5064-e74. doi: 10.1210/clinem/dgab527 [published Online First: 2021/07/18] | | | | | | |
| 80. Romo ML, Schooling CM. Examining the Causal Role of Leptin in Alzheimer Disease: a Mendelian Randomization Study. *Neuroendocrinology* 2017;105(2):182‐88. doi: 10.1159/000475713 | | | | | | |
| 81. Syed AAS, He L, Shi Y. The Potential Effect of Aberrant Testosterone Levels on Common Diseases: A Mendelian Randomization Study. *Genes* 2020;11(7):721. doi: 10.3390/genes11070721 | | | | | | |
| 82. Yuan H, Yang W. Genetically Determined Serum Uric Acid and Alzheimer's Disease Risk. *Journal of Alzheimer's disease : JAD* 2018;65(4):1259-65. doi: 10.3233/JAD-180538 | | | | | | |
| 83. Larsson SC, Gill D, Mason AM, et al. Lipoprotein(a) in Alzheimer, Atherosclerotic, Cerebrovascular, Thrombotic, and Valvular Disease: mendelian Randomization Investigation. *Circulation* 2020;141(22):1826‐28. doi: 10.1161/CIRCULATIONAHA.120.045826 | | | | | | |
| 84. Pan Y, Li H, Wang Y, et al. Causal Effect of Lp(a) [Lipoprotein(a)] Level on Ischemic Stroke and Alzheimer Disease: a Mendelian Randomization Study. *Stroke; a journal of cerebral circulation* 2019;50(12):3532-39. doi: 10.1161/STROKEAHA.119.026872 | | | | | | |
| 85. Yeung CHC, Schooling CM. Systemic inflammatory regulators and risk of Alzheimer's disease: a bidirectional Mendelian-randomization study. *International journal of epidemiology* 2021;50(3):829-40. doi: 10.1093/ije/dyaa241 | | | | | | |
| 86. Williams DM, Karlsson IK, Pedersen NL, et al. Circulating insulin-like growth factors and Alzheimer disease: A mendelian randomization study. *Neurology* 2018;90(4):e291-e97. doi: 10.1212/WNL.0000000000004854 | | | | | | |
| 87. Handy A, Lord J, Green R, et al. Assessing Genetic Overlap and Causality Between Blood Plasma Proteins and Alzheimer's Disease. *Journal of Alzheimer's disease* 2021;83(4):1825-39. doi: 10.3233/JAD-210462 | | | | | | |
| 88. Tsui A, Davis D. Systemic inflammation and causal risk for Alzheimer's dementia: Possibilities and limitations of a Mendelian randomization approach. *Aging medicine (Milton (NSW))* 2018;1(3):249-53. doi: 10.1002/agm2.12046 [published Online First: 2019/07/23] | | | | | | |
| 89. Zhang Z, Wang M, Liu X. C-reactive protein and risk of Alzheimer's disease. *Neurobiology of aging* 2022;109:259-63. doi: 10.1016/j.neurobiolaging.2021.08.010 | | | | | | |
| 90. Rasmussen KL, Nordestgaard BG, Frikke-Schmidt R, et al. An updated Alzheimer hypothesis: complement C3 and risk of Alzheimer's disease-A cohort study of 95,442 individuals. *Alzheimer's & dementia* 2018;14(12):1589‐601. doi: 10.1016/j.jalz.2018.07.223 | | | | | | |
| 91. Yang C, Farias FHG, Ibanez L, et al. Genomic atlas of the proteome from brain, CSF and plasma prioritizes proteins implicated in neurological disorders. *Nature neuroscience* 2021;24(9):1302-12. doi: 10.1038/s41593-021-00886-6 | | | | | | |
| 92. Png G, Barysenka A, Repetto L, et al. Mapping the serum proteome to neurological diseases using whole genome sequencing. *Nature communications* 2021;12(1):7042. doi: 10.1038/s41467-021-27387-1 | | | | | | |
| 93. Zhuang Z, Gao M, Yang R, et al. Causal relationships between gut metabolites and Alzheimer's disease: a bidirectional Mendelian randomization study. *Neurobiology of aging* 2021;100:119.e15-19.e18. doi: 10.1016/j.neurobiolaging.2020.10.022 | | | | | | |
| 94. Zhuang Z, Yang R, Wang W, et al. Associations between gut microbiota and Alzheimer's disease, major depressive disorder, and schizophrenia. *Journal of neuroinflammation* 2020;17(1):288. doi: 10.1186/s12974-020-01961-8 | | | | | | |
| 95. Wu BS, Zhang YR, Li HQ, et al. Cortical structure and the risk for Alzheimer's disease: a bidirectional Mendelian randomization study. *Translational psychiatry* 2021;11(1):476. doi: 10.1038/s41398-021-01599-x [published Online First: 2021/09/17] | | | | | | |
| 96. Yu G, Lu L, Ma Z, et al. Genetically Predicted Telomere Length and Its Relationship With Alzheimer’s Disease. *Frontiers in genetics* 2021;12:595864. doi: 10.3389/fgene.2021.595864 | | | | | | |
| 97. Scheller Madrid A, Rasmussen KL, Rode L, et al. Observational and genetic studies of short telomeres and Alzheimer's disease in 67,000 and 152,000 individuals: a Mendelian randomization study. *European journal of epidemiology* 2020;35(2):147-56. doi: 10.1007/s10654-019-00563-w | | | | | | |
| 98. Zhan Y, Song C, Karlsson R, et al. Telomere Length Shortening and Alzheimer Disease--A Mendelian Randomization Study. *JAMA neurology* 2015;72(10):1202-3. doi: 10.1001/jamaneurol.2015.1513 [published Online First: 2015/10/13] | | | | | | |
| 99. Gao K, Wei C, Zhu J, et al. Exploring the Causal Pathway From Telomere Length to Alzheimer's Disease: An Update Mendelian Randomization Study. *Frontiers in psychiatry* 2019;10:843. doi: 10.3389/fpsyt.2019.00843 [published Online First: 2019/12/06] | | | | | | |
| 100. Guo Y, Yu H. Leukocyte Telomere Length Shortening and Alzheimer's Disease Etiology. *Journal of Alzheimer's disease : JAD* 2019;69(3):881-85. doi: 10.3233/JAD-190134 | | | | | | |
| 101. Baird DA, Liu JZ, Zheng J, et al. Identifying drug targets for neurological and psychiatric disease via genetics and the brain transcriptome. *PLoS genetics* 2021;17(1):e1009224. doi: 10.1371/journal.pgen.1009224 [published Online First: 2021/01/09] | | | | | | |
| 102. Wingo AP, Liu Y, Gerasimov ES, et al. Integrating human brain proteomes with genome-wide association data implicates new proteins in Alzheimer's disease pathogenesis. *Nature genetics* 2021;53(2):143-46. doi: 10.1038/s41588-020-00773-z | | | | | | |
| 103. Zhu J, Liu X, Yin H, et al. Convergent lines of evidence support BIN1 as a risk gene of Alzheimer’s disease. *Human genomics* 2021;15(1):9. doi: 10.1186/s40246-021-00307-6 | | | | | | |
| 104. Liu D, Wang Y, Jing H, et al. Novel DNA methylation loci and genes showing pleiotropic association with Alzheimer's dementia: a network Mendelian randomization analysis. *Epigenetics* 2021;17(7):746-58. doi: 10.1080/15592294.2021.1959735 | | | | | | |
| 105. Zhang H, Wang T, Han Z, et al. Impact of Vitamin D Binding Protein Levels on Alzheimer's Disease: a Mendelian Randomization Study. *Journal of Alzheimer's disease* 2020;74(3):991-98. doi: 10.3233/JAD-191051 | | | | | | |
| 106. Yeung CHC, Lau KWD, Au Yeung SL, et al. Amyloid, tau and risk of Alzheimer's disease: a Mendelian randomization study. *European journal of epidemiology* 2021;36(1):81-88. doi: 10.1007/s10654-020-00683-8 | | | | | | |
