# Supplementary material for: Summarizing attributable factors and evaluating risk of bias of Mendelian randomization studies for Alzheimer’s dementia and cognitive status: a systematic review and meta-analysis
Source: Syst Rev. 2025 Mar 13;14:61. doi: 10.1186/s13643-025-02792-5 (PMC11905674; doi:10.1186/s13643-025-02792-5)
Supplement: Supplementary file 5 — Additional file 5. Table S5. Study characteristics of included MR studies for cognition. [file 13643_2025_2792_MOESM5_ESM.docx]

**Table S5 Study characteristics of included MR studies for cognition.**

| Publication | Risk of factor (exposure) | Outcome | Number of SNPs (Genetic instruments) | Database used by exposure | Database used by outcome (cognitive function) | Participants of exposure: total participants/number of cases | Participants of cognitive function: total participants/number of cases | Ancestry of the exposure population/Ancestry of the cognitive function population | R^2^ | F_statistics |
| --- | --- | --- | --- | --- | --- | --- | --- | --- | --- | --- |
| **Sociodemographic factors** |  |  |  |  |  |  |  |  |  |  |
| Li, M.-2021 [1] | Age at menarche | Cognitive performance | 41 (NA) | Reproductive Genetics consortium (Perry et al. 2014) | SSGAC (Lee et al. 2018) | 182416/NA | 257841/NA | European/European | NA | > 10 |
| Li, M.-2021 [1] | Age at menopause | Cognitive performance | 29 (NA) | Reproductive Genetics consortium (Perry et al. 2014) | SSGAC (Lee et al. 2018) | 69360/NA | 257841/NA | European/European | NA | > 10 |
| **Lifestyle attributes** |  |  |  |  |  |  |  |  |  |  |
| Henry, A.-2019 [2] | Each additional hour/day in sleep duration | Visual memory | 77 (rs10173260, rs10421649, rs10483350, rs1057703, rs10761674, rs10973207, rs11190970, rs112230981, rs113113059, rs11567976, rs11602180, rs11614986, rs11621908, rs11643715, rs11885663, rs12246842, rs12567114, rs12607679, rs12611523, rs1263056, rs12791153, rs13088093, rs13109404, rs151014368, rs1517572rs1553132, rs17427571, rs174560, rs17732997, rs1776776, rs180769, rs1939455, rs1991556, rs205024, rs2072727, rs2079070, rs2139261, rs2192528, rs2231265, rs269054, rs3095508, rs330088, rs34354917, rs34556183, rs34731055, rs35531607, rs365663, rs374153, rs4128364, rs4538155, rs4592416, rs460692, rs4767550, rs55658675, rs56372231, rs61796569, rs61985058, rs62120041, rs6575005, rs7115226, rs72804080, rs73219758, rs7503199, rs75539574, rs7556815, rs7616632, rs7644809, rs7806045, rs7915425, rs7951019, rs80193650, rs8038326, rs8050478, rs915416, rs9345234, rs9382445, rs9903973) | UKB | UKB | 395803/NA | 395803/NA | European/European | 0.65% | 33.86 |
| Henry, A.-2019 [2] | Each additional hour/day in sleep duration | Reaction time | 77 (rs10173260, rs10421649, rs10483350, rs1057703, rs10761674, rs10973207, rs11190970, rs112230981, rs113113059, rs11567976, rs11602180, rs11614986, rs11621908, rs11643715, rs11885663, rs12246842, rs12567114, rs12607679, rs12611523, rs1263056, rs12791153, rs13088093, rs13109404, rs151014368, rs1517572rs1553132, rs17427571, rs174560, rs17732997, rs1776776, rs180769, rs1939455, rs1991556, rs205024, rs2072727, rs2079070, rs2139261, rs2192528, rs2231265, rs269054, rs3095508, rs330088, rs34354917, rs34556183, rs34731055, rs35531607, rs365663, rs374153, rs4128364, rs4538155, rs4592416, rs460692, rs4767550, rs55658675, rs56372231, rs61796569, rs61985058, rs62120041, rs6575005, rs7115226, rs72804080, rs73219758, rs7503199, rs75539574, rs7556815, rs7616632, rs7644809, rs7806045, rs7915425, rs7951019, rs80193650, rs8038326, rs8050478, rs915416, rs9345234, rs9382445, rs9903973) | UKB | UKB | 395803/NA | 395803/NA | European/European | 0.65% | 33.86 |
| Henry, A.-2019 [2] | Per additional hour/day in sleep duration | Decline in visual memory | 77 (rs10173260, rs10421649, rs10483350, rs1057703, rs10761674, rs10973207, rs11190970, rs112230981, rs113113059, rs11567976, rs11602180, rs11614986, rs11621908, rs11643715, rs11885663, rs12246842, rs12567114, rs12607679, rs12611523, rs1263056, rs12791153, rs13088093, rs13109404, rs151014368, rs1517572rs1553132, rs17427571, rs174560, rs17732997, rs1776776, rs180769, rs1939455, rs1991556, rs205024, rs2072727, rs2079070, rs2139261, rs2192528, rs2231265, rs269054, rs3095508, rs330088, rs34354917, rs34556183, rs34731055, rs35531607, rs365663, rs374153, rs4128364, rs4538155, rs4592416, rs460692, rs4767550, rs55658675, rs56372231, rs61796569, rs61985058, rs62120041, rs6575005, rs7115226, rs72804080, rs73219758, rs7503199, rs75539574, rs7556815, rs7616632, rs7644809, rs7806045, rs7915425, rs7951019, rs80193650, rs8038326, rs8050478, rs915416, rs9345234, rs9382445, rs9903973) | UKB | UKB | 395803/NA | 98072/4089 | European/European | 0.65% | 33.86 |
| Henry, A.-2019 [2] | Per additional hour/day in sleep duration | Decline in reaction time | 77 (rs10173260, rs10421649, rs10483350, rs1057703, rs10761674, rs10973207, rs11190970, rs112230981, rs113113059, rs11567976, rs11602180, rs11614986, rs11621908, rs11643715, rs11885663, rs12246842, rs12567114, rs12607679, rs12611523, rs1263056, rs12791153, rs13088093, rs13109404, rs151014368, rs1517572rs1553132, rs17427571, rs174560, rs17732997, rs1776776, rs180769, rs1939455, rs1991556, rs205024, rs2072727, rs2079070, rs2139261, rs2192528, rs2231265, rs269054, rs3095508, rs330088, rs34354917, rs34556183, rs34731055, rs35531607, rs365663, rs374153, rs4128364, rs4538155, rs4592416, rs460692, rs4767550, rs55658675, rs56372231, rs61796569, rs61985058, rs62120041, rs6575005, rs7115226, rs72804080, rs73219758, rs7503199, rs75539574, rs7556815, rs7616632, rs7644809, rs7806045, rs7915425, rs7951019, rs80193650, rs8038326, rs8050478, rs915416, rs9345234, rs9382445, rs9903973) | UKB | UKB | 395803/NA | 17090/622 | European/European | 0.65% | 33.86 |
| Zhou, H.-2020 [3] | Alcohol use disorder | Cognitive performance | 22 (NA) | Million Veteran Program (Kranzler et al. 2019) and Psychiatric Genomics Consortium (Walters et al. 2018) | SSGAC (Lee et al. 2018) | 313959/57564 | 257841/NA | European/European | NA | NA |
| Mahedy, L.-2021 [4] | Alcohol use | Working memory | 87 (NA) | GSCAN (Liu et al. 2019) | ALSPAC | 941280/NA | 2471/NA | European/European | < 2.5% | NA |
| Mahedy, L.-2021 [4] | Alcohol use | Response inhibition | 87 (NA) | GSCAN (Liu et al. 2019) | ALSPAC | 941280/NA | 2446/NA | European/European | < 2.5% | NA |
| Mahedy, L.-2021 [4] | Alcohol use | Emotion recognition | 87 (NA) | GSCAN (Liu et al. 2019) | ALSPAC | 941280/NA | 2560/NA | European/European | < 2.5% | NA |
| Mahedy, L.-2021 [5] | Smoking initiation | Working memory | NA (NA) | ALSPAC | ALSPAC | 5107/1638 | 2471/NA | European/European | NA | 14.63 |
| Mahedy, L.-2021 [5] | Smoking initiation | Response inhibition | NA (NA) | ALSPAC | ALSPAC | 5107/1638 | 2446/NA | European/European | NA | 18.12 |
| Mahedy, L.-2021 [5] | Smoking initiation | Emotion recognition | NA (NA) | ALSPAC | ALSPAC | 5107/1638 | 2560/NA | European/European | NA | 17.57 |
| Mahedy, L.-2021 [5] | Smoking initiation | Working memory | 341 (NA) | GSCAN (Liu et al. 2019) | ALSPAC | NA/NA | 2471/NA | NA/European | NA | NA |
| Mahedy, L.-2021 [5] | Smoking initiation | Response inhibition | 341 (NA) | GSCAN (Liu et al. 2019) | ALSPAC | NA/NA | 2446/NA | NA/European | NA | NA |
| Mahedy, L.-2021 [5] | Smoking initiation | Emotion recognition | 341 (NA) | GSCAN (Liu et al. 2019) | ALSPAC | NA/NA | 2560/NA | NA/European | NA | NA |
| Gage, S. H.-2020 [6] | Smoking initiation | Fluid intelligence | 376 (NA) | GSCAN (Liu et al. 2019) | UKB (Neale et al. 2017) | 607291/311629 | NA/NA | European/European | NA | NA |
| Gage, S. H.-2020 [6] | Smoking initiation | Cognitive ability | 322 (NA) | GSCAN (Liu et al. 2019) | ALSPAC | 607291/311629 | NA/NA | European/European | NA | NA |
| Mahedy, L.-2021 [5] | Lifetime cannabis use | Working memory | 8 (NA) | ICC, 23andME, UKB (Pasman et al. 2018) | ALSPAC | 184765/NA | 2471/NA | European/European | NA | NA |
| Mahedy, L.-2021 [5] | Lifetime cannabis use | Working memory | NA (NA) | ALSPAC | ALSPAC | 5319/1348 | 2471/NA | European/European | NA | 6.7 |
| Mahedy, L.-2021 [5] | Lifetime cannabis use | Response inhibition | 8 (NA) | ICC, 23andME, UKB (Pasman et al. 2018) | ALSPAC | 184765/NA | 2446/NA | European/European | NA | NA |
| Mahedy, L.-2021 [5] | Lifetime cannabis use | Response inhibition | NA (NA) | ALSPAC | ALSPAC | 5319/1348 | 2446/NA | European/European | NA | 8.87 |
| Mahedy, L.-2021 [5] | Lifetime cannabis use | Emotion recognition | 8 (NA) | ICC, 23andME, UKB (Pasman et al. 2018) | ALSPAC | 184765/NA | 2560/NA | European/European | NA | NA |
| Mahedy, L.-2021 [5] | Lifetime cannabis use | Emotion recognition | NA (NA) | ALSPAC | ALSPAC | 5319/1348 | 2560/NA | European/European | NA | 8.79 |
| Yang, F.-2021 [7] | Television watching | Cognitive performance | 136 (rs10041724, rs10054327, rs10145592, rs10189857, rs1022785, rs10234444, rs10246289, rs1031423, rs10427502, rs10737620, rs10771746, rs10772643, rs10876864, rs10890123, rs10932837, rs10940659, rs10994943, rs1674086, rs11130793, rs10415849, rs11201422, rs11218575, rs11245482, rs114328297, rs114600294, rs1156541, rs11654952, rs11657730, rs11689199, rs11714337, rs11763734, rs11810109, rs12105701, rs12272012, rs12289262, rs1243182, rs35094031, rs12491503, rs12541615, rs12554512, rs12725114, rs1278847, rs13029509, rs13107325, rs138256022, rs141184308, rs1421334, rs142710267, rs1451533, rs17207890, rs17379561, rs17512836, rs17727474, rs17789218, rs2034768, rs2045147, rs2073869, rs2092829, rs2164744, rs2173650, rs12210048, rs2447098, rs2460, rs2584597, rs2616830, rs262890, rs2717559, rs2787374, rs303753, rs34864022, rs35574015, rs374722, rs3754970, rs3796386, rs1268446, rs42210, rs4334769, rs4382592, rs4523073, rs4577309, rs4675246, rs4775373, rs4845364, rs4937842, rs4973576, rs55700114, rs12408634, rs56103247, rs56858768, rs57585211, rs6131281, rs6141814, rs62379379, rs62641636, rs6472942, rs6673341, rs6829279, rs6721975, rs6797840, rs6825241, rs6850494, rs6905544, rs6973656, rs6996198, rs7089973, rs7157001, rs71658797, rs7184800, rs7189927, rs7248205, rs72671494, rs72781699, rs72828890, rs72834698, rs749671, rs7564130, rs7693082, rs7693703, rs7700107, rs4382197, rs77215114, rs6997839, rs7335993, rs801733, rs8043253, rs8756, rs9471333, rs9563168, rs4886037, rs9718104, rs973734, rs9834970, rs984409, rs9867121, rs9902312, rs9964724) | UKB (van de Vegte et al. 2020) | SSGAC (Lee et al. 2018) | 408815/NA | 257841/NA | European/European | NA | 5747 |
| Yang, F.-2021 [7] | Computer use | Cognitive performance | 43 (rs10208088, rs10754920, rs113851275, rs11708955, rs11749912, rs12145677, rs12603813, rs12706626, rs13262595, rs136553, rs1448355, rs1469249, rs162894, rs166835, rs198262, rs2068625, rs206965, rs2220599, rs2345941, rs2734849, rs2748985, rs35933007, rs3730399, rs3944151, rs4592851, rs4702, rs4977839, rs55772938, rs6129084, rs6498759, rs66643547, rs6744254, rs6774533, rs6857629, rs6935828, rs7020477, rs7209653, rs7288455, rs73578186, rs78082503, rs784256, rs9372625, rs9477970) | UKB (van de Vegte et al. 2020) | SSGAC (Lee et al. 2018) | 408815/NA | 257841/NA | European/European | NA | 1685 |
| Yang, F.-2021 [7] | Driving behavior | Cognitive performance | 5 (rs10186876, rs1198575, rs4765541, rs6012558, rs9840902) | UKB (van de Vegte et al. 2020) | SSGAC (Lee et al. 2018) | 408815/NA | 257841/NA | European/European | NA | 193 |
| **Anthropometrics** |  |  |  |  |  |  |  |  |  |  |
| Hagenaars, S. P.-2017 [8] | Body mass index | Verbal-numerical reasoning | 70 (rs1000940, rs10132280, rs1016287, rs10182181, rs10733682, rs10938397, rs10968576, rs11030104, rs11057405, rs11126666, rs11165643, rs11191560, rs11583200, rs1167827, rs11688816, rs11727676, rs11847697, rs12286929, rs12401738, rs12429545, rs12446632, rs12566985, rs12885454, rs12940622, rs13021737, rs13078960, rs13107325, rs13191362, rs1516725, rs1528435, rs16851483, rs16951275, rs17024393, rs17094222, rs17405819, rs17724992, rs1808579, rs1928295, rs2033732, rs205262, rs2112347, rs2121279, rs2176598, rs2207139, rs2245368, rs2287019, rs2365389, rs2650492, rs2820292, rs29941, rs3101336, rs3736485, rs3810291, rs3817334, rs3849570, rs3888190, rs4740619, rs543874, rs6477694, rs6567160, rs657452, rs6804842, rs7138803, rs7141420, rs7243357, rs758747, rs7599312, rs7899106, rs7903146, rs9400239) | GWAS of BMI (Locke et al. 2015) | UKB | 236231/NA | 36035/NA | European/European | 2.70% | NA |
| Orri, M.-2021 [9] | Birth weight | Cognitive status: intelligence | 47 (rs1012167, rs10181515, rs10265057, rs10935733, rs11042596, rs11055030, rs11096402, rs112139215, rs1129156, rs116807401, rs11698914, rs11711420, rs12401656, rs13266210, rs134594, rs138715366, rs1480470, rs1547669, rs222857, rs2282978, rs2551347, rs28457693, rs28505901, rs3933326, rs41311445, rs41355649, rs4144829, rs4444073, rs4511593, rs4953353, rs56188432, rs6575803, rs6930558, rs7076938, rs72681869, rs73143584, rs732563, rs7402983, rs753381, rs754868, rs7772579, rs7819593, rs7968682, rs80278614, rs8106042, rs8756, rs9909342) | Early Growth Genetics, UKB (Warrington et al. 2019) | SSGAC (Lee et al. 2018) | 264498/NA | 269867/NA | European/European | 17.29% | 1561.8 |
| Hagenaars, S. P.-2017 [8] | Height | Verbal-numerical reasoning | 331 (rs10883563, rs7899004, rs6584575, rs291979, rs1614303, rs10794175, rs12779328, rs4350272, rs7069985, rs4332428, rs10995319, rs1171615, rs10997979, rs2631676, rs10790381, rs1461503, rs6485978, rs2272566, rs2237886, rs10767838, rs3802758, rs1681630, rs3782089, rs7112925, rs606452, rs2164747, rs2888893, rs11616067, rs2856321, rs11835818, rs7980687, rs10770705, rs11049611, rs11612228, rs10880969, rs2306694, rs10877030, rs17122659, rs8756, rs10748128, rs17783015, rs3825199, rs1199734, rs11618507, rs12323101, rs6561319, rs3118905, rs3818416, rs11616380, rs7319045, rs8017130, rs1950500, rs12435366, rs10131337, rs8006657, rs11624136, rs2093210, rs2781373, rs1980850, rs2058092, rs862034, rs7154721, rs1036477, rs16964211, rs7177711, rs7162825, rs17264185, rs975210, rs12904334, rs5742915, rs11855014, rs2280470, rs7181724, rs4548838, rs1659127, rs2023693, rs11642612, rs4785393, rs8058684, rs217181, rs11648796, rs11640018, rs6420435, rs2326458, rs4843367, rs8052560, rs4640244, rs3809790, rs3760318, rs2338115, rs584828, rs9766, rs4986172, rs318095, rs870183, rs2079795, rs3923086, rs2072268, rs11867479, rs10083886, rs2117563, rs9217, rs1552173, rs4239020, rs692964, rs14062, rs4369779, rs888403, rs11152213, rs8097893, rs11659752, rs8102380, rs7259684, rs8103068, rs8103992, rs11880992, rs2074977, rs7253628, rs4802134, rs4803468, rs2682587, rs2123731, rs891088, rs7517682, rs10779751, rs12120956, rs9428104, rs6658763, rs2298265, rs6688100, rs4656220, rs6694089, rs1325596, rs3814333, rs12137162, rs425277, rs10863936, rs6540834, rs212524, rs991967, rs6696239, rs2806561, rs11799609, rs4601530, rs16834765, rs7544462, rs6600365, rs3014219, rs12855, rs6691924, rs2815379, rs17391694, rs9434723, rs2811594, rs17113369, rs6080830, rs143384, rs4812586, rs2224538, rs7273787, rs1326023, rs2057291, rs6061231, rs1884897, rs2829941, rs2211866, rs9977276, rs7284476, rs738288, rs3885668, rs13388725, rs2166898, rs7567288, rs749234, rs17038954, rs540652, rs12987566, rs6746356, rs833152, rs2345835, rs12693589, rs6435143, rs12470505, rs6761041, rs3116168, rs2289195, rs780094, rs6714546, rs13416119, rs9309101, rs897080, rs12474201, rs354196, rs3791679, rs2120335, rs7568069, rs11684404, rs6439168, rs2597513, rs9880211, rs724016, rs936339, rs6441170, rs9858528, rs720390, rs4686904, rs9841435, rs3915129, rs2633761, rs13088462, rs2581830, rs2034172, rs1658351, rs6794009, rs17806888, rs2175513, rs12330322, rs12639764, rs1562975, rs7659107, rs6838153, rs12513181, rs763318, rs1812175, rs13150868, rs3958122, rs955748, rs2306596, rs1996422, rs6446315, rs13113518, rs17081935, rs9993613, rs17556750, rs2302580, rs13177718, rs1582931, rs26024, rs7701414, rs526896, rs165189, rs4624820, rs2974438, rs4868126, rs7733195, rs422421, rs11750568, rs6879260, rs17410035, rs9292468, rs301901, rs3812040, rs17574650, rs7716219, rs2662027, rs7727731, rs9291926, rs34651, rs820848, rs12519505, rs32855, rs6894139, rs314263, rs6920372, rs2145357, rs1405212, rs1155939, rs4896582, rs6902771, rs11156098, rs1832871, rs991946, rs2763273, rs17330192, rs1047014, rs932445, rs806794, rs1233627, rs9404952, rs6457374, rs12214804, rs16895130, rs10948222, rs9395264, rs12209223, rs9392918, rs310421, rs761391, rs6952113, rs929637, rs6962887, rs273945, rs822531, rs6955948, rs3807931, rs12538407, rs1055144, rs798497, rs552707, rs6462432, rs2715094, rs1113765, rs12669267, rs17807185, rs4725061, rs42039, rs17250196, rs1550162, rs1599473, rs4733724, rs7834383, rs1036821, rs2013265, rs568610, rs6988484, rs9650315, rs2956605, rs429433, rs989393, rs7027110, rs3739707, rs7033487, rs7466269, rs3132297, rs7849585, rs1576900, rs11144688, rs7853235, rs181338, rs7043114, rs817300) | GIANT (Wood et al. 2014) | UKB | 253288/NA | 36035/NA | European/European | 15.00% | NA |
| Sun, D.-2020 [10] | SBP | DSST | 96 (NA) | UKB (Warren et al. 2017) | CARDIA study | 140886/NA | 1369/NA | European/European | NA | NA |
| Sun, D.-2020 [10] | SBP | RAVLT | 92 (NA) | UKB (Warren et al. 2017) | CARDIA study | 140886/NA | 1369/NA | European/European | NA | NA |
| Sun, D.-2020 [10] | SBP | STROOP | 94 (NA) | UKB (Warren et al. 2017) | CARDIA study | 140886/NA | 1369/NA | European/European | NA | NA |
| Hagenaars, S. P.-2017 [8] | SBP | Verbal-numerical reasoning | 20 (rs17367504, rs2932538, rs13082711, rs419076, rs13107325, rs1458038, rs1173771, rs805303, rs11191548, rs1813353, rs932764, rs381815, rs7129220, rs17249754, rs3184504, rs1378942, rs12940887, rs17608766, rs1327235, rs6015450) | GWAS of SBP (Ehret et al. 2011) | UKB | 69395/NA | 36035/NA | European/European | 0.90% | NA |
| Sun, D.-2020 [10] | DBP | DSST | 93 (NA) | UKB (Warren et al. 2017) | CARDIA study | 140886/NA | 1369/NA | European/European | NA | NA |
| Sun, D.-2020 [10] | DBP | RAVLT | 92 (NA) | UKB (Warren et al. 2017) | CARDIA study | 140886/NA | 1369/NA | European/European | NA | NA |
| Sun, D.-2020 [10] | DBP | STROOP | 94 (NA) | UKB (Warren et al. 2017) | CARDIA study | 140886/NA | 1369/NA | European/European | NA | NA |
| Sun, D.-2020 [10] | PP | DSST | 94 (NA) | UKB (Warren et al. 2017) | CARDIA study | 140886/NA | 1369/NA | European/European | NA | NA |
| Sun, D.-2020 [10] | PP | RAVLT | 93 (NA) | UKB (Warren et al. 2017) | CARDIA study | 140886/NA | 1369/NA | European/European | NA | NA |
| Sun, D.-2020 [10] | PP | STROOP | 91 (NA) | UKB (Warren et al. 2017) | CARDIA study | 140886/NA | 1369/NA | European/European | NA | NA |
| **Predisposition to diseases/phenotypes** |  |  |  |  |  |  |  |  |  |  |
| ***Diseases of the circulatory*** |  |  |  |  |  |  |  |  |  |  |
| Hagenaars, S. P.-2017 [8] | Coronary artery disease | Cognitive ability | 18 (rs1122608, rs11556924, rs12413409, rs12936587, rs17114036, rs1746048, rs17465637, rs2306374, rs2895811, rs3798220, rs3825807, rs46522, rs4773144, rs4977574, rs579459, rs599839, rs6725887, rs9982601) | GWAS (Schunkert et al. 2011) | UKB | 86995/22233 | 36035/NA | European/European | 10.00% | NA |
| Kwok, M. K.-2021 [11] | Atrial fibrillation | Cognitive function | 109 (NA) | GWAS (Nielsen et al. 2018) | CHARGE, COGENT, and UKB (Davies et al.2018) | 1030836/60620 | 300486/NA | European/European | 0.94% | 89.9 |
| ***Diseases/phenotypes of the respiratory system*** |  |  |  |  |  |  |  |  |  |  |
| Higbee, D. H.-2021 [12] | Chronic obstructive pulmonary disease | Cognitive function | 67 (NA) | International Chronic Obstructive Pulmonary Disease Genetics Consortium, UKB (Sarkonsakaplat et al. 2019) | CHARGE, COGENT (Davies et al. 2018) | 257811/35735 | 132452/NA | European/European | NA | 52 |
| Higbee, D. H.-2021 [12] | FEV_1_ | Cognitive function | 59 (NA) | UKB, SpiroMeta-GWAS (Shrine et al. 2019) | CHARGE, COGENT (Davies et al. 2018) | 400102/NA | 132452/NA | European/European | NA | 69 |
| Higbee, D. H.-2021 [12] | FEV_1_ | Cognitive function | 298 (NA) | UKB | CHARGE, COGENT (Davies et al. 2018) | 345590/NA | 132452/NA | European/European | NA | NA |
| Higbee, D. H.-2021 [12] | FVC | Cognitive function | 68 (NA) | UKB, SpiroMeta (Shrine et al. 2019) | CHARGE, COGENT (Davies et al. 2018) | 400102/NA | 132452/NA | European/European | NA | 70 |
| Higbee, D. H.-2021 [12] | FEV1/FVC | Cognitive function | 93 (NA) | UKB, SpiroMeta (Shrine et al. 2019) | CHARGE, COGENT (Davies et al. 2018) | 400102/NA | 132452/NA | European/European | NA | 148 |
| Higbee, D. H.-2021 [12] | FEV1, FVC, FEV1/FVC, peak expiratory flow | Cognitive function | 173 (NA) | UKB, SpiroMeta (Shrine et al. 2019) | CHARGE, COGENT (Davies et al. 2018) | 400102/NA | 132452/NA | European/European | NA | 111 |
| Higbee, D. H.-2021 [12] | Forced expiratory volume | Cognitive function | 381 (NA) | UKB | CHARGE, COGENT (Davies et al. 2018) | 345590/NA | 132452/NA | European/European | NA | NA |
| ***Diseases/phenotypes of Endocrine System*** |  |  |  |  |  |  |  |  |  |  |
| Ware, E. B.-2021 [13] | T2DM | CIND | NA (Polygenic score) | Health and Retirement Study (2020) | Health and Retirement Study (2020) | 7979/NA | 7979/NA | European/European | NA | 234.1 |
| Hagenaars, S. P.-2017 [8] | T2DM | Verbal-numerical reasoning | 8 (rs4402960, rs7756992, rs849135, rs3802177, rs10811661, rs1111875, rs7903146, rs9936385) | DIAGRAM (Morris et al. 2012 stage-1) | UKB | 69033/12171 | 36035/NA | European/European | 5.70% | NA |
| Garfield, V.-2021 [14] | T2DM | Reaction time | 157 (NA) | Combined GWAS from 32 studies (Mahajan et al. 2018) | UKB | 898130/74124 | 349326/NA | European/European | 1.50% | 27.43 |
| Garfield, V.-2021 [14] | T2DM | Reaction time | 77 (NA) | DIAGRAM (Mahajan et al. 2014) | UKB | NA/NA | 349326/NA | NA/European | NA | 30.88 |
| Garfield, V.-2021 [14] | T2DM | Visual memory | 157 (NA) | Combined GWAS from 32 studies (Mahajan et al. 2018) | UKB | 898130/74124 | 349326/NA | European/European | 1.50% | 27.43 |
| Garfield, V.-2021 [14] | T2DM | Visual memory | 77 (NA) | DIAGRAM (Mahajan et al. 2014) | UKB | NA/NA | 349326/NA | NA/European | NA | 30.88 |
| Garfield, V.-2021 [14] | HbA1c | Reaction time | 51 (rs1046896, rs10774625, rs10823343, rs10830963, rs11086054, rs11224302, rs11248914, rs11558471, rs11603334, rs11619319, rs11708067, rs11964178, rs12621844, rs13134327, rs1467311, rs1558902, rs174577, rs17509001, rs17533903, rs17747324, rs1800562, rs198846, rs2110073, rs2191349, rs2375278, rs2383208, rs2408955, rs267738, rs282587, rs3782123, rs3824065, rs4607517, rs4737009, rs4745982, rs4783565, rs4820268, rs4894799, rs560887, rs579459, rs592423, rs6474359, rs6980507, rs7040409, rs7616006, rs7756992, rs8192675, rs837763, rs857691, rs9604573, rs9818758, rs9914988) | GWAS (Wheeler et al. 2017) | UKB | NA/NA | NA/NA | European/European | 2.80% | 164.6 |
| Garfield, V.-2021 [14] | HbA1c | Visual memory | 51 (rs1046896, rs10774625, rs10823343, rs10830963, rs11086054, rs11224302, rs11248914, rs11558471, rs11603334, rs11619319, rs11708067, rs11964178, rs12621844, rs13134327, rs1467311, rs1558902, rs174577, rs17509001, rs17533903, rs17747324, rs1800562, rs198846, rs2110073, rs2191349, rs2375278, rs2383208, rs2408955, rs267738, rs282587, rs3782123, rs3824065, rs4607517, rs4737009, rs4745982, rs4783565, rs4820268, rs4894799, rs560887, rs579459, rs592423, rs6474359, rs6980507, rs7040409, rs7616006, rs7756992, rs8192675, rs837763, rs857691, rs9604573, rs9818758, rs9914988) | GWAS (Wheeler et al. 2017) | UKB | NA/NA | NA/NA | European/European | 2.80% | 164.6 |
| ***Mental symptom/phenotype*** |  |  |  |  |  |  |  |  |  |  |
| Rosoff, D. B.-2020 [15] | Suicide attempt | Cognitive performance | 13 (NA) | iPSYCH GWASs (Erlangsen et al. 2019) | SSGAC (Lee et al. 2018) | 50264/6024 | 257841/NA | Predominantly European /European | 0.60% | 23.430 |
| Fitzgerald, J.-2022 [16] | Schizophrenia | Cognitive resilience | 152 (NA) | UKB | UKB | 266543/NA | 266543/NA | European/European | NA | NA |
| Fitzgerald, J.-2022 [16] | Bipolar disorder | Cognitive resilience | 13 (NA) | UKB | UKB | 266543/NA | 266543/NA | European/European | NA | NA |
| ***Disease of herpes virus infection*** |  |  |  |  |  |  |  |  |  |  |
| Kwok, M. K.-2021 [17] | HSV infection | Cognitive function | 108 (rs72661517, rs137875660, rs141762587, rs79791423, rs28569185, rs78247505, rs76796791, rs1075688, rs148117129, rs139749675, rs72817942, rs139867497, rs1519647, rs16833509, rs80189034, rs111341098, rs147768671, rs114511528, rs117938012, rs144279141, rs142955037, rs62315917, rs114371318, rs117373772, rs75582075, rs12188041, rs111498642, rs74347763, rs191358934, rs139923785, rs145242611, rs61178465, rs191303010, rs7768621, rs150383498, rs72867568, rs151015442, rs62420569, rs12525756, rs9388000, rs143504577, rs114262802, rs145951674, rs116959781, rs192787921, rs144299282, rs75919604, rs78901239, rs75021745, rs145366114, rs149469629, rs11496171, rs140099206, rs73725299, rs78478942, rs148133887, rs142149837, rs11993881, rs143679028, rs141892831, rs139760814, rs7875054, rs72732948, rs180883500, rs76023281, rs189507880, rs74817377, rs185240410, rs117794161, rs138685265, rs118033341, rs11197586, rs149674827, rs150480597, rs12284824, rs181807044, rs189126732, rs117334944, rs71455470, rs76033287, rs146446580, rs116862258, rs16943898, rs142579103, rs113971128, rs74070632, rs35738901, rs77353959, rs72641495, rs12147304, rs73481666, rs117346960, rs117829204, rs12944569, rs117368106, rs77107066, rs11653760, rs76396910, rs79965232, rs117243019, rs116955951, rs17055749, rs149126057, rs181023919, rs143187995, rs76548129, rs146863228, rs139828174) | UKB | CHARGE, COGENT, and UKB (Davies et al.2018) | 361141/154 | 300486/NA | European/European | 0.66% | 24.92 |
| Kwok, M. K.-2021 [17] | HSV infection | Cognitive function | 10 (rs17732209, rs115789906, rs73036068, rs2905775, rs2532923, rs885950, rs74762609, rs4360170, rs3132452, rs16974161, rs28391720) | US 23&Me Study (Tian et al. 2017) | CHARGE, COGENT, and UKB (Davies et al.2018) | 88440/25108 | 300486/NA | European/European | 0.33% | 29.61 |
| Kwok, M. K.-2021 [17] | HSV infection | Cognitive function | 7 (rs9439664, rs61782495, rs2689714, rs7864004, rs2449629, rs7573884, rs12932696) | French Milieu Interieur cohort (Scepanovic et al. 2018) | CHARGE, COGENT, and UKB (Davies et al.2018) | 1000/853 | 300486/NA | European/European | 53.60% | 118.5 |
| **Dietary intake** |  |  |  |  |  |  |  |  |  |  |
| Zhou, A.-2018 [18] | Habitual coffee consumption | Global cognition | 2 (rs2472297 (CYP1A1/2) and rs6968865 (AHR)) | 1958 British birth cohort , UKB, Mothers of Avon Longitudinal Study of Parents and Children, Northern Finland Birth Cohorts 1966, Cardiovascular Risk in Young Finns Study, Helsinki Birth Cohort Study, Prospective Investigation of the Vasculature in Uppsala Seniors, Uppsala Longitudinal Study of Adult Men, Swedish twin registry and TwinGene studies | 1958 British birth cohort , UKB, Mothers of Avon Longitudinal Study of Parents and Children, Northern Finland Birth Cohorts 1966, Cardiovascular Risk in Young Finns Study, Helsinki Birth Cohort Study, Prospective Investigation of the Vasculature in Uppsala Seniors, Uppsala Longitudinal Study of Adult Men, Swedish twin registry and TwinGene studies | 415530/300760 | 300760/NA | European (White)/European (White) | NA | NA |
| Zhou, A.-2018 [18] | Habitual coffee consumption | Memory scores | 2 (rs2472297 (CYP1A1/2) and rs6968865 (AHR)) | 1958 British birth cohort , UKB, Mothers of Avon Longitudinal Study of Parents and Children, Northern Finland Birth Cohorts 1966, Cardiovascular Risk in Young Finns Study, Helsinki Birth Cohort Study, Prospective Investigation of the Vasculature in Uppsala Seniors, Uppsala Longitudinal Study of Adult Men, Swedish twin registry and TwinGene studies | 1958 British birth cohort , UKB, Mothers of Avon Longitudinal Study of Parents and Children, Northern Finland Birth Cohorts 1966, Cardiovascular Risk in Young Finns Study, Helsinki Birth Cohort Study, Prospective Investigation of the Vasculature in Uppsala Seniors, Uppsala Longitudinal Study of Adult Men, Swedish twin registry and TwinGene studies | 415530/300760 | 301804/NA | European (White)/European (White) | NA | NA |
| Zhou, A.-2018 [18] | Habitual coffee consumption | Domain-specific cognitive measures: reaction time | 6 (rs17685, rs9902453, rs1260326, rs1481012, rs7800944, and rs6265 (POR, EFCAB5, GCKR, ABCG2, MLXIPL, and BDN)) | 1958 British birth cohort , UKB, Mothers of Avon Longitudinal Study of Parents and Children, Northern Finland Birth Cohorts 1966, Cardiovascular Risk in Young Finns Study, Helsinki Birth Cohort Study, Prospective Investigation of the Vasculature in Uppsala Seniors, Uppsala Longitudinal Study of Adult Men, Swedish twin registry and TwinGene studies | UKB | 415530/300760 | 288905/NA | European (White)/European (White) | 0.33% | 952.6 |
| Zhou, A.-2018 [18] | Habitual coffee consumption | Domain-specific cognitive measures: pairs matching | 6 (rs17685, rs9902453, rs1260326, rs1481012, rs7800944, and rs6265 (POR, EFCAB5, GCKR, ABCG2, MLXIPL, and BDN)) | 1958 British birth cohort , UKB, Mothers of Avon Longitudinal Study of Parents and Children, Northern Finland Birth Cohorts 1966, Cardiovascular Risk in Young Finns Study, Helsinki Birth Cohort Study, Prospective Investigation of the Vasculature in Uppsala Seniors, Uppsala Longitudinal Study of Adult Men, Swedish twin registry and TwinGene studies | UKB | 415530/300760 | 290574/NA | European (White)/European (White) | 0.33% | 952.6 |
| Zhou, A.-2018 [18] | Habitual coffee consumption | Domain-specific cognitive measures: reasoning | 6 (rs17685, rs9902453, rs1260326, rs1481012, rs7800944, and rs6265 (POR, EFCAB5, GCKR, ABCG2, MLXIPL, and BDN)) | 1958 British birth cohort , UKB, Mothers of Avon Longitudinal Study of Parents and Children, Northern Finland Birth Cohorts 1966, Cardiovascular Risk in Young Finns Study, Helsinki Birth Cohort Study, Prospective Investigation of the Vasculature in Uppsala Seniors, Uppsala Longitudinal Study of Adult Men, Swedish twin registry and TwinGene studies | UKB | 415530/300760 | 93512/NA | European (White)/European (White) | 0.33% | 952.6 |
| Zhou, A.-2018 [18] | Habitual coffee consumption | Domain-specific cognitive measures: prospective memory | 6 (rs17685, rs9902453, rs1260326, rs1481012, rs7800944, and rs6265 (POR, EFCAB5, GCKR, ABCG2, MLXIPL, and BDN)) | 1958 British birth cohort , UKB, Mothers of Avon Longitudinal Study of Parents and Children, Northern Finland Birth Cohorts 1966, Cardiovascular Risk in Young Finns Study, Helsinki Birth Cohort Study, Prospective Investigation of the Vasculature in Uppsala Seniors, Uppsala Longitudinal Study of Adult Men, Swedish twin registry and TwinGene studies | UKB | 415530/300760 | 95340/NA | European (White)/European (White) | 0.33% | 952.6 |
| Maddock, J.-2017 [19] | 25(OH)D concentrations | Global cognitive function | 2 (rs12794714 (CYP2R1), rs12785878 (DHCR7)) | 9 cohorts (1958BC, CoLaus, ESTHER, HBCS, NFBC66, PIVUS, Tromsø, ULSAM, and YFS) | 17 cohorts (1958BC, CoLaus, ESTHER, HBCS, NFBC66, PIVUS, Tromsø, ULSAM, YFS, ASPS, ELSA, HRS, STR, TwinGene, UKB, UKHLS, and WII) | 18830/NA | 153187/NA | European (White)/European (White) | 0.60% | 113.50 |
| Maddock, J.-2017 [19] | 25(OH)D concentrations | Memory cognitive function | 2 (rs12794714 (CYP2R1), rs12785878 (DHCR7)) | 9 cohorts (1958BC, CoLaus, ESTHER, HBCS, NFBC66, PIVUS, Tromsø, ULSAM, and YFS) | 17 cohorts (1958BC, CoLaus, ESTHER, HBCS, NFBC66, PIVUS, Tromsø, ULSAM, YFS, ASPS, ELSA, HRS, STR, TwinGene, UKB, UKHLS, and WII) | 18830/NA | 155882/NA | European (White)/European (White) | 0.60% | 113.50 |
| Maddock, J.-2017 [19] | 25(OH)D concentrations | Cognitive domain-specific effects (Pairs matching) | 2 (rs12794714 (CYP2R1), rs12785878 (DHCR7)) | 9 cohorts (1958BC, CoLaus, ESTHER, HBCS, NFBC66, PIVUS, Tromsø, ULSAM, and YFS) | UKB | 18830/NA | 110545/NA | European (White)/European (White) | 0.60% | 113.50 |
| Maddock, J.-2017 [19] | 25(OH)D concentrations | Reaction time | 2 (rs12794714 (CYP2R1), rs12785878 (DHCR7)) | 9 cohorts (1958BC, CoLaus, ESTHER, HBCS, NFBC66, PIVUS, Tromsø, ULSAM, and YFS) | UKB | 18830/NA | 109911/NA | European (White)/European (White) | 0.60% | 113.5 |
| Maddock, J.-2017 [19] | 25(OH)D concentrations | Cognitive domain-specific effects (Reasoning) | 2 (rs12794714 (CYP2R1), rs12785878 (DHCR7)) | 9 cohorts (1958BC, CoLaus, ESTHER, HBCS, NFBC66, PIVUS, Tromsø, ULSAM, and YFS) | UKB | 18830/NA | 35603/NA | European (White)/European (White) | 0.60% | 113.5 |
| Maddock, J.-2017 [19] | 25(OH)D concentrations | Cognitive domain-specific effects (Prospective memory) | 2 (rs12794714 (CYP2R1), rs12785878 (DHCR7)) | 9 cohorts (1958BC, CoLaus, ESTHER, HBCS, NFBC66, PIVUS, Tromsø, ULSAM, and YFS) | UKB | 18830/NA | 36311/NA | European (White)/European (White) | 0.60% | 113.5 |
| Liu, H.-2021 [20] | Plasma vitamin C | Cognitive performance | 11 (rs10051765, rs10136000, rs117885456, rs13028225, rs174547, rs2559850, rs33972313, rs56738967, rs6693447, rs7740812, rs9895661) | Fenland study, EPIC-InterAct study, EPIC-Norfolk study, EPIC-CVD study (Zheng et al. 2018) | SSGAC (Rietveld et al. 2014) | 52018/NA | 257841/NA | European /European | 1.85% | NA |
| **Biochemical index** |  |  |  |  |  |  |  |  |  |  |
| Fu, M.-2021 [21] | HDL-C | CIND | NA (polygenic score) | Health and Retirement Study (waves 2006–2012) | Health and Retirement Study (waves 2006–2012) | 7707/NA | 7707/NA | European/European | NA | NA |
| Fu, M.-2021 [21] | HDL-C | Total cognition score | NA (polygenic score) | Health and Retirement Study (waves 2006–2012) | Health and Retirement Study (waves 2006–2012) | 5270/NA | 5270/NA | European/European | NA | NA |
| Fu, M.-2021 [21] | HDL-C | Episodic memory (Immediate word recall) | NA (polygenic score) | Health and Retirement Study (waves 2006–2012) | Health and Retirement Study (waves 2006–2012) | 7869/NA | 7869/NA | European/European | NA | NA |
| Fu, M.-2021 [21] | HDL-C | Episodic memory (Delayed word recall) | NA (polygenic score) | Health and Retirement Study (waves 2006–2012) | Health and Retirement Study (waves 2006–2012) | 7869/NA | 7869/NA | European/European | NA | NA |
| Fu, M.-2021 [21] | HDL-C | Mental status (Serial 7 subtraction) | NA (polygenic score) | Health and Retirement Study (waves 2006–2012) | Health and Retirement Study (waves 2006–2012) | 7869/NA | 7869/NA | European/European | NA | NA |
| Fu, M.-2021 [21] | HDL-C | Mental status (Backward count from 20) | NA (polygenic score) | Health and Retirement Study (waves 2006–2012) | Health and Retirement Study (waves 2006–2012) | 7869/NA | 7869/NA | European/European | NA | NA |
| Fu, M.-2021 [21] | HDL-C | Vocabulary | NA (polygenic score) | Health and Retirement Study (waves 2006–2012) | Health and Retirement Study (waves 2006–2012) | 2714/NA | 2714/NA | European/European | NA | NA |
| Fu, M.-2021 [21] | TC | CIND | NA (polygenic score) | Health and Retirement Study (waves 2006–2012) | Health and Retirement Study (waves 2006–2012) | 8590/NA | 8590/NA | European/European | NA | NA |
| Fu, M.-2021 [21] | TC | Total cognition score | NA (polygenic score) | Health and Retirement Study (waves 2006–2012) | Health and Retirement Study (waves 2006–2012) | 5924/NA | 5924/NA | European/European | NA | NA |
| Fu, M.-2021 [21] | TC | Episodic memory: immediate word recall | NA (polygenic score) | Health and Retirement Study (waves 2006–2012) | Health and Retirement Study (waves 2006–2012) | 8775/NA | 8775/NA | European/European | NA | NA |
| Fu, M.-2021 [21] | TC | Episodic memory: delayed word recall | NA (polygenic score) | Health and Retirement Study (waves 2006–2012) | Health and Retirement Study (waves 2006–2012) | 8775/NA | 8775/NA | European/European | NA | NA |
| Fu, M.-2021 [21] | TC | Mental status: serial 7 subtraction | NA (polygenic score) | Health and Retirement Study (waves 2006–2012) | Health and Retirement Study (waves 2006–2012) | 8775/NA | 8775/NA | European/European | NA | NA |
| Fu, M.-2021 [21] | TC | Mental status: backward count from 20 | NA (polygenic score) | Health and Retirement Study (waves 2006–2012) | Health and Retirement Study (waves 2006–2012) | 8775/NA | 8775/NA | European/European | NA | NA |
| Fu, M.-2021 [21] | TC | Vocabulary | NA (polygenic score) | Health and Retirement Study (waves 2006–2012) | Health and Retirement Study (waves 2006–2012) | 3166/NA | 3166/NA | European/European | NA | NA |
| Dunk, M. M.-2021 [22] | TC | Early MCI | NA (APOE3R versus APOE5+) | ADNI between 2004 and 2017 | ADNI between 2004 and 2017 | 1534/NA | 1534/NA | The United States and Canada/the United States and Canada | NA | > 10 |
| Dunk, M. M.-2021 [22] | TC | Late MCI | NA (APOE3R versus APOE5+) | ADNI between 2004 and 2017 | ADNI between 2004 and 2017 | 1534/NA | 1534/NA | The United States and Canada/the United States and Canada | NA | > 10 |
| Dunk, M. M.-2021 [22] | TC | Early & Late MCI | NA (APOE3R versus APOE5+) | ADNI between 2004 and 2017 | ADNI between 2004 and 2017 | 1534/NA | 1534/NA | The United States and Canada/the United States and Canada | NA | > 10 |
| Winchester, L. M.-2018 [23] | MCH | Reaction time | NA (NA) | UKB | UKB | 37323/NA | 37323/NA | European/European | NA | NA |
| Winchester, L. M.-2018 [23] | MCH | Reaction time | NA (NA) | UKB | UKB | 37323/NA | 335423/NA | European/European | NA | NA |
| Winchester, L. M.-2018 [23] | MCH | Numeric memory | NA (NA) | UKB | UKB | 12474/NA | 12474/NA | European/European | NA | NA |
| Winchester, L. M.-2018 [23] | MCH | Numeric memory | NA (NA) | UKB | UKB | 12474/NA | 335423/NA | European/European | NA | NA |
| Winchester, L. M.-2018 [23] | MCH | Prospective memory | NA (NA) | UKB | UKB | 38104/NA | 38104/NA | European/European | NA | NA |
| Winchester, L. M.-2018 [23] | MCH | Prospective memory | NA (NA) | UKB | UKB | 38104/NA | 335423/NA | European/European | NA | NA |
| Winchester, L. M.-2018 [23] | MCH | Visual memory | NA (NA) | UKB | UKB | 116391/NA | 116391/NA | European/European | NA | NA |
| Winchester, L. M.-2018 [23] | MCH | Verbal: numeric reasoning | NA (NA) | UKB | UKB | 115782/NA | 115782/NA | European/European | NA | NA |
| Winchester, L. M.-2018 [23] | MCH | Verbal: numeric reasoning | 110 (NA) | UKB | UKB | 115782/NA | 335423/NA | European/European | NA | NA |
| Winchester, L. M.-2018 [23] | MCH | Verbal: numeric reasoning | NA (NA) | UKB | UKB | 37323/NA | 335423/NA | European/European | NA | NA |
| Winchester, L. M.-2018 [23] | RDW | Reaction time | NA (NA) | UKB | UKB | 37323/NA | 37323/NA | European/European | NA | NA |
| Winchester, L. M.-2018 [23] | RDW | Verbal: numeric reasoning | NA (NA) | UKB | UKB | 115782/NA | 115782/NA | European/European | NA | NA |
| Winchester, L. M.-2018 [23] | RDW | Numeric memory | NA (NA) | UKB | UKB | 12474/NA | 12474/NA | European/European | NA | NA |
| Winchester, L. M.-2018 [23] | RDW | Visual memory | NA (NA) | UKB | UKB | 116391/NA | 116391/NA | European/European | NA | NA |
| Winchester, L. M.-2018 [23] | RDW | Prospective memory | NA (NA) | UKB | UKB | 38104/NA | 38104/NA | European/European | NA | NA |
| Efstathiadou, A.-2019 [24] | SUA | Cognitive performance | 28 (rs10480300, rs10821905, rs11264341, rs1165151, rs1171614, rs1178977, rs12498742, rs1260326, rs1394125, rs1471633, rs17050272, rs17632159, rs17786744, rs2078267, rs2231142, rs2941484, rs3741414, rs478607 rs653178, rs6598541, rs675209, rs6770152, rs7188445, rs7193778, rs7224610, rs729761, rs7953704, rs7976059) | Global Urate Genetics Consortium | SSGAC (Lee et al. 2018) | 110347/NA | 257841/NA | European/European | 5.80% | > 10 |
| Richard, E.-2021 [25] | SUA | Numeric memory | 297 (Polygenic scores) | UKB | UKB | 39359/NA | 39359/NA | European/European | 5.40% | NA |
| Richard, E.-2021 [25] | SUA | Verbal: numeric reasoning | 297 (Polygenic scores) | UKB | UKB | 124834/NA | 124834/NA | European/European | 5.40% | NA |
| Richard, E.-2021 [25] | SUA | Reaction time | 297 (Polygenic scores) | UKB | UKB | 357590/NA | 357590/NA | European/European | 5.40% | NA |
| Richard, E.-2021 [25] | SUA | Visual memory (% errors) | 297 (Polygenic scores) | UKB | UKB | 359664/NA | 359664/NA | European/European | 5.40% | NA |
| Richard, E.-2021 [25] | Serum creatinine: eGFRcre | Numeric memory | 453 (Polygenic scores) | UKB | UKB | 39359/NA | 39359/NA | European/European | 4.00% | NA |
| Richard, E.-2021 [25] | Serum creatinine: eGFRcre | Reaction time | 453 (Polygenic scores) | UKB | UKB | 357590/NA | 357590/NA | European/European | 4.00% | NA |
| Richard, E.-2021 [25] | Serum creatinine: eGFRcre | Verbal: numeric reasoning | 453 (Polygenic scores) | UKB | UKB | 124834/NA | 124834/NA | European/European | 4.00% | NA |
| Richard, E.-2021 [25] | Serum creatinine: eGFRcre | Visual memory | 453 (Polygenic scores) | UKB | UKB | 359664/NA | 359664/NA | European/European | 4.00% | NA |
| Richard, E.-2021 [25] | Serum cystatin C: eGFRcys | Numeric memory | 4 (Polygenic scores) | UKB | UKB | 39359/NA | 39359/NA | European/European | 3.00% | NA |
| Richard, E.-2021 [25] | Serum cystatin C: eGFRcys | Verbal: numeric reasoning | 4 (Polygenic scores) | UKB | UKB | 124834/NA | 124834/NA | European/European | 3.00% | NA |
| Richard, E.-2021 [25] | Serum cystatin C: eGFRcys | Reaction time | 4 (Polygenic scores) | UKB | UKB | 357590/NA | 357590/NA | European/European | 3.00% | NA |
| Richard, E.-2021 [25] | Serum cystatin C: eGFRcys | Visual memory | 4 (Polygenic scores) | UKB | UKB | 359664/NA | 359664/NA | European/European | 3.00% | NA |
| Richard, E.-2021 [25] | Log ACR | Numeric memory | 76 (Polygenic scores) | UKB | UKB | 39359/NA | 39359/NA | European/European | 0.60% | NA |
| Richard, E.-2021 [25] | Log ACR | Verbal-numeric reasoning | 76 (Polygenic scores) | UKB | UKB | 124834/NA | 124834/NA | European/European | 0.60% | NA |
| Richard, E.-2021 [25] | Log ACR | Reaction time | 76 (Polygenic scores) | UKB | UKB | 357590/NA | 357590/NA | European/European | 0.60% | NA |
| Richard, E.-2021 [25] | Log ACR | Visual memory | 76 (Polygenic scores) | UKB | UKB | 359664/NA | 359664/NA | European/European | 0.60% | NA |
| **Biomarkers of immunity and inflammation** |  |  |  |  |  |  |  |  |  |  |
| Png, G.-2021 [26] | CD33 | Cognitive performance | 3 (NA) | NA | SSGAC (Lee et al. 2018) | NA/NA | 257841/NA | NA/European | NA | NA |
| **Omics traits** |  |  |  |  |  |  |  |  |  |  |
| ***Neuroimaging feature*** |  |  |  |  |  |  |  |  |  |  |
| Fitzgerald, J.-2022 [16] | White matter | Cognitive resilience | 9 (NA) | UKB | UKB | 266543/NA | 266543/NA | European /European | NA | NA |
| Fitzgerald, J.-2022 [16] | Cerebral whate matter (left) | Cognitive resilience | 12 (NA) | UKB | UKB | 266543/NA | 266543/NA | European/European | NA | NA |
| Fitzgerald, J.-2022 [16] | Cerebral whate matter (right) | Cognitive resilience | 12 (NA) | UKB | UKB | 266543/NA | 266543/NA | European/European | NA | NA |
| ***Leukocyte telomere length*** |  |  |  |  |  |  |  |  |  |  |
| Hägg, S.-2017 [27] | Telomere length | Cognitive ability | 7 (rs11125529, rs10936599, rs7675998, rs2736100, rs9420907, rs8105767, rs755017 (GRS)) | ENGAGE study | ENGAGE study | 12283/NA | 12283/NA | European/European | NA | 36 |
| Hägg, S.-2017 [27] | Telomere length | Cognitive ability | 7 (rs11125529, rs10936599, rs7675998, rs2736100, rs9420907, rs8105767, rs755017 (GRS)) | ENGAGE study | CHARGE Consortium (Davies et al. 2015) | 12283/NA | 53949/NA | European/European | NA | 36 |
| Hägg, S.-2017 [27] | Telomere length | Mini-mental state exam | 7 (rs11125529, rs10936599, rs7675998, rs2736100, rs9420907, rs8105767, rs755017 (GRS)) | ENGAGE study | ENGAGE study | 7066/NA | 7066/NA | European/European | NA | 36 |
| Hägg, S.-2017 [27] | Telomere length | DSST | 7 (rs11125529, rs10936599, rs7675998, rs2736100, rs9420907, rs8105767, rs755017 (GRS)) | ENGAGE study | ENGAGE study | 4419/NA | 4419/NA | European/European | NA | 36 |
| Hägg, S.-2017 [27] | Telomere length | Block design test | 7 (rs11125529, rs10936599, rs7675998, rs2736100, rs9420907, rs8105767, rs755017 (GRS)) | ENGAGE study | ENGAGE study | 5001/NA | 5001/NA | European/European | NA | 36 |
| Hägg, S.-2017 [27] | Telomere length | Memory: Verbal memory or Picture learning test | 7 (rs11125529, rs10936599, rs7675998, rs2736100, rs9420907, rs8105767, rs755017 (GRS)) | ENGAGE study | ENGAGE study | 13060/NA | 13060/NA | European/European | NA | 36 |
| Hägg, S.-2017 [27] | Telomere length | STROOP | 7 (rs11125529, rs10936599, rs7675998, rs2736100, rs9420907, rs8105767, rs755017 (GRS)) | ENGAGE study | ENGAGE study | 2940/NA | 2940/NA | European/European | NA | 36 |
| ***DNA methylation*** |  |  |  |  |  |  |  |  |  |  |
| Caramaschi.-2017 [28] | DNA methylation: cg10543947 | Cognitive performance | 1 (rs5750236) | ALSPAC-ARIES (Boyd et al. 2013) | SSGAC (Rietveld et al. 2014) | 771/NA | 106736/NA | White ethnicity/White ethnicity | NA | NA |
| Caramaschi.-2017 [28] | DNA methylation: cg15676719 | Cognitive performance | 1 (rs1890131) | ALSPAC-ARIES (Boyd et al. 2013) | SSGAC (Rietveld et al. 2014) | 771/NA | 106736/NA | White ethnicity/White ethnicity | NA | NA |
| Caramaschi.-2017 [28] | DNA methylation: cg10543947 | Cognitive performance: off spring’s cognition | 1 (rs5750236) | ALSPAC-ARIES (Boyd et al. 2013) | ALSPAC-ARIES (Boyd et al. 2013) | 771/NA | 3354-3843/NA | White ethnicity/White ethnicity | NA | NA |
| Caramaschi.-2017 [28] | DNA methylation: cg15676719 | Cognitive performance: off spring’s cognition | 1 (rs1890131) | ALSPAC-ARIES (Boyd et al. 2013) | ALSPAC-ARIES (Boyd et al. 2013) | 771/NA | 3354-3843/NA | White ethnicity/White ethnicity | NA | NA |
| ***Proteome*** |  |  |  |  |  |  |  |  |  |  |
| Png, G.-2021 [26] | Dipeptidase 1 | Cognitive performance | 3 (NA) | NA | SSGAC (Lee et al. 2018) | NA/NA | 257841/NA | NA/European | NA | NA |
| When a noun in the table appears three or more times, the abbreviation is used to indicate it, as follows: ACR, albumin to creatinine ratio; ALSPAC, Avon Longitudinal Study of Parents and Children; CARDIA, Coronary Artery Risk Development in Young Adults; CHARGE, Cohort of Heart and Aging Research in Genomic Epidemiology; CIND, cognitive impairment-non dementia; COGENT, Cognitive Genomics Consortium; DBP, diastolic blood pressure; DSST, Digit Symbol Substitution Test; eGFRcre, estimated glomerular filtration rate creatinine; eGFRcys, estimated glomerular filtration rate cystatin C; ENGAGE, European Network for Genetic and Genomic Epidemiology; EPIC, European Prospective Investigation into Cancer and Nutrition; FEV1, forced expiratory volume in 1 second; FVC, forced vital capacity; GSCAN, GWAS & Sequencing Consortium of Alcohol and Nicotine; HDL-C, high-density lipoprotein cholesterol; HSV, herpes simplex virus; ICC, International Cannabis Consortium; MCH, mean corpuscular haemoglobin; MCI, mild cognitive impairment; PP, pulse pressure; RAVLT, Rey Auditory Verbal Learning Test; RDW, red blood cell distribution width; SBP, systolic blood pressure; SSGAC, Social Science Genetic Association Consortium; STROOP, Stroop interference score; SUA, serum uric acid; TC, total cholesterol; T2DM, type 2 diabetes mellitus; UKB, UK Biobank; 25(OH)D, 25-hydroxyvitamin D. | | | | | | | | | | |
|  |  |  |  |  |  |  |  |  |  |  |
| **References** |  |  |  |  |  |  |  |  |  |  |
| 1. Li M, Lin J, Liang S, et al. The role of age at menarche and age at menopause in Alzheimer's disease: evidence from a bidirectional mendelian randomization study. *Aging* 2021;13(15):19722-49. doi: 10.18632/aging.203384 | | | | | | | | | | |
| 2. Henry A, Katsoulis M, Masi S, et al. The relationship between sleep duration, cognition and dementia: a Mendelian randomization study. *International journal of epidemiology* 2019;48(3):849-60. doi: 10.1093/ije/dyz071 | | | | | | | | | | |
| 3. Zhou H, Sealock JM, Sanchez-Roige S, et al. Genome-wide meta-analysis of problematic alcohol use in 435,563 individuals yields insights into biology and relationships with other traits. *Nat Neurosci* 2020;23(7):809-18. doi: 10.1038/s41593-020-0643-5 [published Online First: 2020/05/27] | | | | | | | | | | |
| 4. Mahedy L, Suddell S, Skirrow C, et al. Alcohol use and cognitive functioning in young adults: improving causal inference. *Addiction (Abingdon, England)* 2021;116(2):292-302. doi: 10.1111/add.15100 [published Online First: 2020/04/27] | | | | | | | | | | |
| 5. Mahedy L, Wootton R, Suddell S, et al. Testing the association between tobacco and cannabis use and cognitive functioning: findings from an observational and Mendelian randomization study. *Drug and alcohol dependence* 2021;221:108591. doi: 10.1016/j.drugalcdep.2021.108591 | | | | | | | | | | |
| 6. Gage SH, Sallis HM, Lassi G, et al. Does smoking cause lower educational attainment and general cognitive ability? Triangulation of causal evidence using multiple study designs. *Psychological medicine* 2020;52(8):1578-86. doi: 10.1017/S0033291720003402 | | | | | | | | | | |
| 7. Yang F, Chen S, Qu Z, et al. Genetic Liability to Sedentary Behavior in Relation to Stroke, Its Subtypes and Neurodegenerative Diseases: A Mendelian Randomization Study. *Frontiers in Aging Neuroscience* 2021;13:757388. doi: 10.3389/fnagi.2021.757388 | | | | | | | | | | |
| 8. Hagenaars SP, Gale CR, Deary IJ, et al. Cognitive ability and physical health: a Mendelian randomization study. *Scientific reports* 2017;7(1):2651. doi: 10.1038/s41598-017-02837-3 | | | | | | | | | | |
| 9. Orri M, Pingault JB, Turecki G, et al. Contribution of birth weight to mental health, cognitive and socioeconomic outcomes: Two-sample Mendelian randomisation. *British Journal of Psychiatry* 2021;219(3):507-14. doi: 10.1192/bjp.2021.15 | | | | | | | | | | |
| 10. Sun D, Thomas EA, Launer LJ, et al. Association of blood pressure with cognitive function at midlife: a Mendelian randomization study. *BMC medical genomics* 2020;13(1):121. doi: 10.1186/s12920-020-00769-y | | | | | | | | | | |
| 11. Kwok MK, Schooling CM. Mendelian randomization study on atrial fibrillation and cardiovascular disease subtypes. *Scientific reports* 2021;11(1):18682. doi: 10.1038/s41598-021-98058-w | | | | | | | | | | |
| 12. Higbee DH, Granell R, Hemani G, et al. Lung function, COPD and cognitive function: a multivariable and two sample Mendelian randomization study. *BMC pulmonary medicine* 2021;21(1):246. doi: 10.1186/s12890-021-01611-6 | | | | | | | | | | |
| 13. Ware EB, Morataya C, Fu M, et al. Type 2 Diabetes and Cognitive Status in the Health and Retirement Study: a Mendelian Randomization Approach. *Frontiers in genetics* 2021;12:634767. doi: 10.3389/fgene.2021.634767 | | | | | | | | | | |
| 14. Garfield V, Farmaki AE, Fatemifar G, et al. Relationship Between Glycemia and Cognitive Function, Structural Brain Outcomes, and Dementia: A Mendelian Randomization Study in the UK Biobank. *Diabetes* 2021;70(10):2313-21. doi: 10.2337/db20-0895 [published Online First: 2021/02/27] | | | | | | | | | | |
| 15. Rosoff DB, Kaminsky ZA, McIntosh AM, et al. Educational attainment reduces the risk of suicide attempt among individuals with and without psychiatric disorders independent of cognition: a bidirectional and multivariable Mendelian randomization study with more than 815,000 participants. *Translational psychiatry* 2020;10(1):388. doi: 10.1038/s41398-020-01047-2 | | | | | | | | | | |
| 16. Fitzgerald J, Fahey L, Holleran L, et al. Thirteen Independent Genetic Loci Associated with Preserved Processing Speed in a Study of Cognitive Resilience in 330,097 Individuals in the UK Biobank. *Genes* 2022;13(1):122. doi: 10.3390/genes13010122 | | | | | | | | | | |
| 17. Kwok MK, Schooling CM. Herpes simplex virus and Alzheimer's disease: a Mendelian randomization study. *Neurobiology of aging* 2021;99:101.e11-01.e13. doi: 10.1016/j.neurobiolaging.2020.09.025 | | | | | | | | | | |
| 18. Zhou A, Taylor AE, Karhunen V, et al. Habitual coffee consumption and cognitive function: a Mendelian randomization meta-analysis in up to 415,530 participants. *Scientific reports* 2018;8(1):7526. doi: 10.1038/s41598-018-25919-2 | | | | | | | | | | |
| 19. Maddock J, Zhou A, Cavadino A, et al. Vitamin D and cognitive function: A Mendelian randomisation study. *Sci Rep* 2017;7(1):13230. doi: 10.1038/s41598-017-13189-3 [published Online First: 2017/10/19] | | | | | | | | | | |
| 20. Liu H, Zhang Y, Hu Y, et al. Mendelian randomization to evaluate the effect of plasma vitamin C levels on the risk of Alzheimer’s disease. *Genes and Nutrition* 2021;16(1):19. doi: 10.1186/s12263-021-00700-9 | | | | | | | | | | |
| 21. Fu M, Bakulski KM, Higgins C, et al. Mendelian Randomization of Dyslipidemia on Cognitive Impairment Among Older Americans. *Frontiers in Neurology* 2021;12:660212. doi: 10.3389/fneur.2021.660212 | | | | | | | | | | |
| 22. Dunk MM, Driscoll I. Total Cholesterol and APOE-Related Risk for Alzheimer's Disease in the Alzheimer's Disease Neuroimaging Initiative. *Journal of Alzheimer's disease : JAD* 2021;85(4):1519-28. doi: 10.3233/JAD-215091 | | | | | | | | | | |
| 23. Winchester LM, Powell J, Lovestone S, et al. Red blood cell indices and anaemia as causative factors for cognitive function deficits and for Alzheimer's disease. *Genome medicine* 2018;10(1):51. doi: 10.1186/s13073-018-0556-z | | | | | | | | | | |
| 24. Efstathiadou A, Gill D, McGrane F, et al. Genetically Determined Uric Acid and the Risk of Cardiovascular and Neurovascular Diseases: A Mendelian Randomization Study of Outcomes Investigated in Randomized Trials. *Journal of the American Heart Association* 2019;8(17):e012738. doi: 10.1161/JAHA.119.012738 | | | | | | | | | | |
| 25. Richard E, McEvoy L, Cao S, et al. Biomarkers of kidney function and cognitive ability: a mendelian randomization study. *Circulation* 2021;143:118071. doi: 10.1161/circ.143.suppl_1.028 | | | | | | | | | | |
| 26. Png G, Barysenka A, Repetto L, et al. Mapping the serum proteome to neurological diseases using whole genome sequencing. *Nature communications* 2021;12(1):7042. doi: 10.1038/s41467-021-27387-1 | | | | | | | | | | |
| 27. Hägg S, Zhan Y, Karlsson R, et al. Short telomere length is associated with impaired cognitive performance in European ancestry cohorts. *Translational psychiatry* 2017;7(4):e1100. doi: 10.1038/tp.2017.73 | | | | | | | | | | |
| 28. Caramaschi D, Sharp GC, Nohr EA, et al. Exploring a causal role of DNA methylation in the relationship between maternal vitamin B12 during pregnancy and child's IQ at age 8, cognitive performance and educational attainment: a two-step Mendelian randomization study. *Human molecular genetics* 2017;26(15):3001-13. doi: 10.1093/hmg/ddx164 | | | | | | | | | | |
