# Supplementary material for: Summarizing attributable factors and evaluating risk of bias of Mendelian randomization studies for Alzheimer’s dementia and cognitive status: a systematic review and meta-analysis
Source: Syst Rev. 2025 Mar 13;14:61. doi: 10.1186/s13643-025-02792-5 (PMC11905674; doi:10.1186/s13643-025-02792-5)
Supplement: Supplementary file 6 — Additional file 6. Table S6. The association between cognitive status and risk factors. [file 13643_2025_2792_MOESM6_ESM.docx]

**Table S6 The association between cognitive status and risk factors.**

| Publication | Risk of factor (exposure) | Outcome | Odds ratio (95% confidence interval) | *P* | Meta-analysis_Cochran's Q | Meta-analysis_df | Meta-analysis_I2 |
| --- | --- | --- | --- | --- | --- | --- | --- |
| **Sociodemographic factors** |  |  |  |  |  |  |  |
| Li, M.-2021 [1] | Age at menarche | Cognitive performance | 1.005 (0.981-1.030) | 0.658 |  |  |  |
| Li, M.-2021 [1] | Age at menopause | Cognitive performance | 0.998 (0.992-1.005) | 0.585 |  |  |  |
| **Lifestyle attributes** |  |  |  |  |  |  |  |
| Henry, A.-2019 [2] | Each additional hour/day in sleep duration | Visual memory | 1.027 (1.000–1.056) | 0.050 |  |  |  |
| Henry, A.-2019 [2] | Each additional hour/day in sleep duration | Reaction time | 1.011 (1.003-1.019) | 0.008 |  |  |  |
| Henry, A.-2019 [2] | Per additional hour/day in sleep duration | Decline in visual memory | 1.096 (0.765–1.570) | 0.619 |  |  |  |
| Henry, A.-2019 [2] | Per additional hour/day in sleep duration | Decline in reaction time | 1.280 (0.490–3.349) | 0.614 |  |  |  |
| Zhou, H.-2020 [3] | Alcohol use disorder | Cognitive performance | 0.970 (0.970-0.970) | 0.460 |  |  |  |
| Mahedy, L.-2021 [4] | Alcohol use | Working memory | 1.330 (0.657-2.691) | 0.430 |  |  |  |
| Mahedy, L.-2021 [4] | Alcohol use | Response inhibition | 0.725 (0.353-1.477) | 0.380 |  |  |  |
| Mahedy, L.-2021 [4] | Alcohol use | Emotion recognition | 1.028 (0.577-1.840) | 0.930 |  |  |  |
| Mahedy, L.-2021 [5] | Smoking initiation | Working memory | 0.684 (0.221-2.117) | 0.510 |  |  |  |
| Mahedy, L.-2021 [5] | Smoking initiation | Response inhibition | 0.763 (0.270-2.160) | 0.610 |  |  |  |
| Mahedy, L.-2021 [5] | Smoking initiation | Emotion recognition | 0.589 (0.204-1.699) | 0.330 |  |  |  |
| Mahedy, L.-2021 [5] | Smoking initiation | Working memory | 1.010 (0.942-1.336) | 0.210 |  |  |  |
| Mahedy, L.-2021 [5] | Smoking initiation | Response inhibition | 1.174 (0.970-1.419) | 0.100 |  |  |  |
| Mahedy, L.-2021 [5] | Smoking initiation | Emotion recognition | 1.000 (0.835-1.197) | 0.970 |  |  |  |
| Gage, S. H.-2020 [6] | Smoking initiation | Fluid intelligence | 0.442 (0.354-0.552) | <0.001 |  |  |  |
| Gage, S. H.-2020 [6] | Smoking initiation | Cognitive ability | 0.667 (0.486-0.915) | 0.012 |  |  |  |
| Meta-analysis | Lifetime cannabis use | Working memory | 0.633 (0.016-23.886) | 0.359 | 1.123 | 1 | 0.110 |
| Mahedy, L.-2021 [5] | Lifetime cannabis use | Working memory | 0.691 (0.487-0.980) | 0.040 |  |  |  |
| Mahedy, L.-2021 [5] | Lifetime cannabis use | Working memory | 0.244 (0.037-1.632) | 0.140 |  |  |  |
| Meta-analysis | Lifetime cannabis use | Response inhibition | 1.040 (0.112-9.611) | 0.861 | 0.0008 | 1 | 0.000 |
| Mahedy, L.-2021 [5] | Lifetime cannabis use | Response inhibition | 1.041 (0.726-1.477) | 0.850 |  |  |  |
| Mahedy, L.-2021 [5] | Lifetime cannabis use | Response inhibition | 1.020 (0.270-3.857) | 0.980 |  |  |  |
| Meta-analysis | Lifetime cannabis use | Emotion recognition | 0.927 (0.062-13.807) | 0.781 | 0.0033 | 1 | 0.000 |
| Mahedy, L.-2021 [5] | Lifetime cannabis use | Emotion recognition | 0.923 (0.595-1.433) | 0.710 |  |  |  |
| Mahedy, L.-2021 [5] | Lifetime cannabis use | Emotion recognition | 0.961 (0.259-3.561) | 0.950 |  |  |  |
| Yang, F.-2021 [7] | Television watching | Cognitive performance | 0.631 (0.577-0.691) | <0.001 |  |  |  |
| Yang, F.-2021 [7] | Computer use | Cognitive performance | 1.916 (1.649-2.226) | <0.001 |  |  |  |
| Yang, F.-2021 [7] | Driving behavior | Cognitive performance | 0.449 (0.247-0.827) | 0.010 |  |  |  |
| **Anthropometrics** |  |  |  |  |  |  |  |
| Hagenaars, S. P.-2017 [8] | Body mass index | Verbal-numerical reasoning | 0.966 (0.863-1.08) | 0.544 |  |  |  |
| Orri, M.-2021 [9] | Birth weight | Cognitive status: intelligence | 0.932 (0.878-0.98) | 0.010 |  |  |  |
| Hagenaars, S. P.-2017 [8] | Height | Verbal-numerical reasoning | 1.026 (0.991-1.063) | 0.133 |  |  |  |
| Sun, D.-2020 [10] | SBP | DSST | 0.613 (0.449-0.844) | 0.002 |  |  |  |
| Sun, D.-2020 [10] | SBP | RAVLT | 0.932 (0.869-1.000) | 0.046 |  |  |  |
| Sun, D.-2020 [10] | SBP | STROOP | 1.259 (1.051-1.507) | 0.011 |  |  |  |
| Hagenaars, S. P.-2017 [8] | SBP | Verbal-numerical reasoning | 0.998 (0.990-1.006) | 0.636 |  |  |  |
| Sun, D.-2020 [10] | DBP | DSST | 0.463 (0.357-0.795) | 0.005 |  |  |  |
| Sun, D.-2020 [10] | DBP | RAVLT | 0.951 (0.852-1.062) | 0.370 |  |  |  |
| Sun, D.-2020 [10] | DBP | STROOP | 1.271 (0.932-1.716) | 0.126 |  |  |  |
| Sun, D.-2020 [10] | PP | DSST | 0.512 (0.313-0.835) | 0.008 |  |  |  |
| Sun, D.-2020 [10] | PP | RAVLT | 0.932 (0.844-1.030) | 0.165 |  |  |  |
| Sun, D.-2020 [10] | PP | STROOP | 1.310 (0.990-1.751) | 0.058 |  |  |  |
| **Predisposition to diseases/phenotypes** |  |  |  |  |  |  |  |
| ***Diseases of the circulatory*** |  |  |  |  |  |  |  |
| Hagenaars, S. P.-2017 [8] | Coronary artery disease | Cognitive ability | 0.982 (0.956-1.009) | 0.234 |  |  |  |
| Kwok, M. K.-2021 [11] | Atrial fibrillation | Cognitive function | 0.999 (0.993-1.004) | 0.620 |  |  |  |
| ***Diseases/phenotypes of the respiratory system*** |  |  |  |  |  |  |  |
| Higbee, D. H.-2021 [12] | Chronic obstructive pulmonary disease | Cognitive function | 0.992 (0.977-1.008) | 0.350 |  |  |  |
| Meta-analysis | FEV1 | Cognitive function | 0.945 (0.697-1.282) | 0.257 | 0.036 | 1 | 0.000 |
| Higbee, D. H.-2021 [12] | FEV_1_ | Cognitive function | 0.951 (0.880-1.029) | 0.440 |  |  |  |
| Higbee, D. H.-2021 [12] | FEV_1_ | Cognitive function | 0.942 (0.888-0.999) | < 0.001 |  |  |  |
| Higbee, D. H.-2021 [12] | FVC | Cognitive function | 0.996 (0.939-1.056) | 0.870 |  |  |  |
| Higbee, D. H.-2021 [12] | FEV1/FVC | Cognitive function | 1.010 (0.971-1.050) | 0.520 |  |  |  |
| Higbee, D. H.-2021 [12] | FEV1, FVC, FEV1/FVC, peak expiratory flow | Cognitive function | 0.998 (0.960-1.038) | 0.860 |  |  |  |
| Higbee, D. H.-2021 [12] | Forced expiratory volume | Cognitive function | 0.942 (0.923-0.960) | < 0.001 |  |  |  |
| ***Diseases/phenotypes of Endocrine System*** |  |  |  |  |  |  |  |
| Ware, E. B.-2021 [13] | T2DM | CIND | 1.040 (0.900-1.210) | > 0.050 |  |  |  |
| Hagenaars, S. P.-2017 [8] | T2DM | Verbal-numerical reasoning | 1.010 (0.981-1.040) | 0.532 |  |  |  |
| Meta-analysis | T2DM | Reaction time | NA | NA | NA | 1 | NA |
| Garfield, V.-2021 [14] | T2DM | Reaction time | 1.000 (1.000-1.000) | > 0.050 |  |  |  |
| Garfield, V.-2021 [14] | T2DM | Reaction time | 1.000 (0.990, 1.000) | > 0.050 |  |  |  |
| Meta-analysis | T2DM | Visual memory | 1.000 (0.970,1.031) | > 0.999 | NA | 1 | NA |
| Garfield, V.-2021 [14] | T2DM | Visual memory | 1.000 (0.990, 1.000) | > 0.050 |  |  |  |
| Garfield, V.-2021 [14] | T2DM | Visual memory | 1.000 (0.990, 1.020) | > 0.050 |  |  |  |
| Garfield, V.-2021 [14] | HbA1c | Reaction time | 1.000 (0.990, 1.020) | > 0.050 |  |  |  |
| Garfield, V.-2021 [14] | HbA1c | Visual memory | 0.990 (0.960, 1.020) | > 0.050 |  |  |  |
| ***Mental symptom/phenotype*** |  |  |  |  |  |  |  |
| Rosoff, D. B.-2020 [15] | Suicide attempt | Cognitive performance | 1.010 (0.988-1.033) | 0.360 |  |  |  |
| Fitzgerald, J.-2022 [16] | Schizophrenia | Cognitive resilience | 0.934 (0.910-0.959) | <0.001 |  |  |  |
| Fitzgerald, J.-2022 [16] | Bipolar disorder | Cognitive resilience | 1.000 (0.933-1.072) | 0.992 |  |  |  |
| ***Disease of herpes virus infection*** |  |  |  |  |  |  |  |
| Meta-analysis | HSV infection | Cognitive function | 1.000 (1.000-1.000) | > 0.999 | NA | 2 | NA |
| Kwok, M. K.-2021 [17] | HSV infection | Cognitive function | 1.030 (0.631-1.684) | 0.900 |  |  |  |
| Kwok, M. K.-2021 [17] | HSV infection | Cognitive function | 1.003 (0.994-1.012) | 0.550 |  |  |  |
| Kwok, M. K.-2021 [17] | HSV infection | Cognitive function | 1.000 (1.000-1.000) | 0.960 |  |  |  |
| **Dietary intake** |  |  |  |  |  |  |  |
| Zhou, A.-2018 [18] | Habitual coffee consumption | Global cognition | 0.999 (0.991-1.008) | 0.870 |  |  |  |
| Zhou, A.-2018 [18] | Habitual coffee consumption | Memory scores | 0.999 (0.995-1.002) | 0.510 |  |  |  |
| Zhou, A.-2018 [18] | Habitual coffee consumption | Domain-specific cognitive measures: reaction time | 1.000 (0.990-1.000) | 0.168 |  |  |  |
| Zhou, A.-2018 [18] | Habitual coffee consumption | Domain-specific cognitive measures: pairs matching | 1.000 (0.990-1.000) | 0.460 |  |  |  |
| Zhou, A.-2018 [18] | Habitual coffee consumption | Domain-specific cognitive measures: reasoning | 1.000 (0.990-1.000) | 0.597 |  |  |  |
| Zhou, A.-2018 [18] | Habitual coffee consumption | Domain-specific cognitive measures: prospective memory | 1.020 (1.000-1.041) | 0.047 |  |  |  |
| Maddock, J.-2017 [19] | 25(OH)D concentrations | Global cognitive function | 1.000 (0.991-1.008) | 0.940 |  |  |  |
| Maddock, J.-2017 [19] | 25(OH)D concentrations | Memory cognitive function | 0.998 (0.991-1.005) | 0.530 |  |  |  |
| Maddock, J.-2017 [19] | 25(OH)D concentrations | Cognitive domain-specific effects (Pairs matching) | 1.000 (0.990-1.000) | 0.290 |  |  |  |
| Maddock, J.-2017 [19] | 25(OH)D concentrations | Reaction time | 1.000 (0.990-1.010) | 0.830 |  |  |  |
| Maddock, J.-2017 [19] | 25(OH)D concentrations | Cognitive domain-specific effects (Reasoning) | 1.010 (0.990-1.020) | 0.320 |  |  |  |
| Maddock, J.-2017 [19] | 25(OH)D concentrations | Cognitive domain-specific effects (Prospective memory) | 0.990 (0.961-1.020) | 0.470 |  |  |  |
| Liu, H.-2021 [20] | Plasma vitamin C | Cognitive performance | 1.007 (0.958-1.059) | 0.775 |  |  |  |
| **Biochemical index** |  |  |  |  |  |  |  |
| Fu, M.-2021 [21] | HDL-C | CIND | 0.910 (0.680-1.230) | > 0.050 |  |  |  |
| Fu, M.-2021 [21] | HDL-C | Total cognition score | 0.771 (0.512-1.150) | > 0.050 |  |  |  |
| Fu, M.-2021 [21] | HDL-C | Episodic memory (Immediate word recall) | 0.905 (0.811-1.020) | > 0.050 |  |  |  |
| Fu, M.-2021 [21] | HDL-C | Episodic memory (Delayed word recall) | 0.878 (0.763-1.010) | > 0.050 |  |  |  |
| Fu, M.-2021 [21] | HDL-C | Mental status (Serial 7 subtraction) | 1.030 (0.914-1.162) | > 0.050 |  |  |  |
| Fu, M.-2021 [21] | HDL-C | Mental status (Backward count from 20) | 1.000 (0.961-1.030) | > 0.050 |  |  |  |
| Fu, M.-2021 [21] | HDL-C | Vocabulary | 0.923 (0.733-1.174) | > 0.050 |  |  |  |
| Fu, M.-2021 [21] | TC | CIND | 0.860 (0.580-1.260) | > 0.050 |  |  |  |
| Fu, M.-2021 [21] | TC | Total cognition score | 1.221 (0.554-2.718) | > 0.050 |  |  |  |
| Fu, M.-2021 [21] | TC | Episodic memory: immediate word recall | 1.051 (0.835-1.323) | > 0.050 |  |  |  |
| Fu, M.-2021 [21] | TC | Episodic memory: delayed word recall | 1.062 (0.803-1.405) | > 0.050 |  |  |  |
| Fu, M.-2021 [21] | TC | Mental status: serial 7 subtraction | 1.062 (0.844-1.336) | > 0.050 |  |  |  |
| Fu, M.-2021 [21] | TC | Mental status: backward count from 20 | 1.030 (0.970-1.094) | > 0.050 |  |  |  |
| Fu, M.-2021 [21] | TC | Vocabulary | 0.932 (0.583-1.492) | > 0.050 |  |  |  |
| Dunk, M. M.-2021 [22] | TC | Early MCI | 1.050 (1.010-3.220) | < 0.050 |  |  |  |
| Dunk, M. M.-2021 [22] | TC | Late MCI | 1.130 (1.050-11.700) | < 0.050 |  |  |  |
| Dunk, M. M.-2021 [22] | TC | Early & Late MCI | 1.110 (1.040-7.320) | < 0.050 |  |  |  |
| Meta-analysis | MCH | Reaction time | 1.005 (0.958-1.054) | 0.398 | 0.324 | 1 | 0.000 |
| Winchester, L. M.-2018 [23] | MCH | Reaction time | 1.009 (0.994-1.024) | 0.232 |  |  |  |
| Winchester, L. M.-2018 [23] | MCH | Reaction time | 1.004 (0.995-1.012) | 0.363 |  |  |  |
| Meta-analysis | MCH | Numeric memory | 1.025 (0.876-1.201) | 0.294 | 1.181 | 1 | 0.153 |
| Winchester, L. M.-2018 [23] | MCH | Numeric memory | 1.045 (1.002-1.090) | 0.039 |  |  |  |
| Winchester, L. M.-2018 [23] | MCH | Numeric memory | 1.017 (0.992-1.043) | 0.181 |  |  |  |
| Meta-analysis | MCH | Prospective memory | 1.003 (0.777-1.295) | 0.900 | 1.449 | 1 | 0.310 |
| Winchester, L. M.-2018 [23] | MCH | Prospective memory | 1.032 (0.972-1.095) | 0.308 |  |  |  |
| Winchester, L. M.-2018 [23] | MCH | Prospective memory | 0.989 (0.954-1.024) | 0.534 |  |  |  |
| Winchester, L. M.-2018 [23] | MCH | Visual memory | 0.990 (0.977-1.004) | 0.152 |  |  |  |
| Meta-analysis | MCH | Verbal: numeric reasoning | 1.033 (1.005-1.061) | 0.037 | 2.3979 | 2 | 0.166 |
| Winchester, L. M.-2018 [23] | MCH | Verbal: numeric reasoning | 1.047 (1.020-1.075) | 0.001 |  |  |  |
| Winchester, L. M.-2018 [23] | MCH | Verbal: numeric reasoning | 1.036 (1.017-1.055) | <0.001 |  |  |  |
| Winchester, L. M.-2018 [23] | MCH | Verbal: numeric reasoning | 1.023 (1.006-1.040) | 0.007 |  |  |  |
| Winchester, L. M.-2018 [23] | RDW | Reaction time | 0.987 (0.947-1.027) | 0.453 |  |  |  |
| Winchester, L. M.-2018 [23] | RDW | Verbal: numeric reasoning | 0.888 (0.826-0.954) | 0.003 |  |  |  |
| Winchester, L. M.-2018 [23] | RDW | Numeric memory | 0.895 (0.791-1.012) | 0.071 |  |  |  |
| Winchester, L. M.-2018 [23] | RDW | Visual memory | 1.044 (0.991-1.099) | 0.131 |  |  |  |
| Winchester, L. M.-2018 [23] | RDW | Prospective memory | 0.895 (0.751-1.068) | 0.186 |  |  |  |
| Efstathiadou, A.-2019 [24] | SUA | Cognitive performance | 0.980 (0.961-1.010) | 0.160 |  |  |  |
| Richard, E.-2021 [25] | SUA | Numeric memory | 0.961 (0.923-1.010) | 0.100 |  |  |  |
| Richard, E.-2021 [25] | SUA | Verbal: numeric reasoning | 0.970 (0.932-1.010) | 0.130 |  |  |  |
| Richard, E.-2021 [25] | SUA | Reaction time | 1.271 (0.403-4.015) | 0.680 |  |  |  |
| Richard, E.-2021 [25] | SUA | Visual memory (% errors) | 0.600 (0.244-1.492) | 0.270 |  |  |  |
| Richard, E.-2021 [25] | Serum creatinine: eGFRcre | Numeric memory | 0.970 (0.932-1.020) | 0.300 |  |  |  |
| Richard, E.-2021 [25] | Serum creatinine: eGFRcre | Reaction time | 0.560 (0.142-2.203) | 0.400 |  |  |  |
| Richard, E.-2021 [25] | Serum creatinine: eGFRcre | Verbal: numeric reasoning | 1.030 (0.990-1.073) | 0.150 |  |  |  |
| Richard, E.-2021 [25] | Serum creatinine: eGFRcre | Visual memory | 2.096 (0.942-4.665) | 0.070 |  |  |  |
| Richard, E.-2021 [25] | Serum cystatin C: eGFRcys | Numeric memory | 0.980 (0.942-1.020) | 0.290 |  |  |  |
| Richard, E.-2021 [25] | Serum cystatin C: eGFRcys | Verbal: numeric reasoning | 1.006 (0.99-1.284) | 0.770 |  |  |  |
| Richard, E.-2021 [25] | Serum cystatin C: eGFRcys | Reaction time | 0.811 (0.154-4.263) | 0.790 |  |  |  |
| Richard, E.-2021 [25] | Serum cystatin C: eGFRcys | Visual memory | 1.297 (0.644-2.612) | 0.460 |  |  |  |
| Richard, E.-2021 [25] | Log ACR | Numeric memory | 0.990 (0.819-1.197) | 0.890 |  |  |  |
| Richard, E.-2021 [25] | Log ACR | Verbal-numeric reasoning | 0.970 (0.844-1.116) | 0.690 |  |  |  |
| Richard, E.-2021 [25] | Log ACR | Reaction time | 123.965 (2.509-6124.179) | 0.010 |  |  |  |
| Richard, E.-2021 [25] | Log ACR | Visual memory | 14.013 (0.942-225.879) | 0.055 |  |  |  |
| **Biomarkers of immunity and inflammation** |  |  |  |  |  |  |  |
| Png, G.-2021 [26] | CD33 | Cognitive performance | 0.990 (0.980, 0.990) | 0.002 |  |  |  |
| **Omics traits** |  |  |  |  |  |  |  |
| ***Neuroimaging feature*** |  |  |  |  |  |  |  |
| Fitzgerald, J.-2022 [16] | White matter | Cognitive resilience | 1.142 (0.996-1.310) | 0.049 |  |  |  |
| Fitzgerald, J.-2022 [16] | Cerebral whate matter (left) | Cognitive resilience | 1.162 (1.053-1.281) | 0.005 |  |  |  |
| Fitzgerald, J.-2022 [16] | Cerebral whate matter (right) | Cognitive resilience | 1.179 (1.06-1.312) | 0.002 |  |  |  |
| ***Leukocyte telomere length*** |  |  |  |  |  |  |  |
| Meta-analysis | Telomere length | Cognitive ability | 1.087 (0.700-1.687) | 0.251 | 0.111 | 1 | 0.000 |
| Hägg, S.-2017 [27] | Telomere length | Cognitive ability | 1.040 (0.795-1.358) | 0.778 |  |  |  |
| Hägg, S.-2017 [27] | Telomere length | Cognitive ability | 1.090 (1.016-1.169) | 0.016 |  |  |  |
| Hägg, S.-2017 [27] | Telomere length | Mini-mental state exam | 1.338 (0.951-1.879) | 0.095 |  |  |  |
| Hägg, S.-2017 [27] | Telomere length | DSST | 0.984 (0.646-1.499) | 0.941 |  |  |  |
| Hägg, S.-2017 [27] | Telomere length | Block design test | 0.825 (0.552-1.234) | 0.349 |  |  |  |
| Hägg, S.-2017 [27] | Telomere length | Memory: Verbal memory or Picture learning test | 0.978 (0.768-1.246) | 0.860 |  |  |  |
| Hägg, S.-2017 [27] | Telomere length | STROOP | 0.550 (0.325-0.931) | 0.026 |  |  |  |
| ***DNA methylation*** |  |  |  |  |  |  |  |
| Caramaschi.-2017 [28] | DNA methylation: cg10543947 | Cognitive performance | 0.993 (0.955-1.033) | 0.740 |  |  |  |
| Caramaschi.-2017 [28] | DNA methylation: cg15676719 | Cognitive performance | 0.984 (0.965-1.004) | 0.110 |  |  |  |
| Caramaschi.-2017 [28] | DNA methylation: cg10543947 | Cognitive performance: off spring’s cognition | 7.614 (0.344-168.477) | 0.200 |  |  |  |
| Caramaschi.-2017 [28] | DNA methylation: cg15676719 | Cognitive performance: off spring’s cognition | 0.154 (0.027-0.865) | 0.030 |  |  |  |
| ***Proteome*** |  |  |  |  |  |  |  |
| Png, G.-2021 [26] | Dipeptidase 1 | Cognitive performance | 0.986 (0.98-0.992) | <0.001 |  |  |  |
| When a noun in the table appears three or more times, the abbreviation is used to indicate it, as follows: ACR, albumin to creatinine ratio; CIND, cognitive impairment-non dementia; DBP, diastolic blood pressure; DSST, Digit Symbol Substitution Test; eGFRcre, estimated glomerular filtration rate creatinine; eGFRcys, estimated glomerular filtration rate cystatin C; FEV1, forced expiratory volume in 1 second; FVC, forced vital capacity; HDL-C, high-density lipoprotein cholesterol; HSV, herpes simplex virus; MCH, mean corpuscular haemoglobin; MCI, mild cognitive impairment; PP, pulse pressure; RAVLT, Rey Auditory Verbal Learning Test; RDW, red blood cell distribution width; SBP, systolic blood pressure; STROOP, Stroop interference score; SUA, serum uric acid; TC, total cholesterol; T2DM, type 2 diabetes mellitus; 25(OH)D, 25-hydroxyvitamin D. | | | | | | | |
|  |  |  |  |  |  |  |  |
| **References** |  |  |  |  |  |  |  |
| 1. Li M, Lin J, Liang S, et al. The role of age at menarche and age at menopause in Alzheimer's disease: evidence from a bidirectional mendelian randomization study. *Aging* 2021;13(15):19722-49. doi: 10.18632/aging.203384 | | | | | | | |
| 2. Henry A, Katsoulis M, Masi S, et al. The relationship between sleep duration, cognition and dementia: a Mendelian randomization study. *International journal of epidemiology* 2019;48(3):849-60. doi: 10.1093/ije/dyz071 | | | | | | | |
| 3. Zhou H, Sealock JM, Sanchez-Roige S, et al. Genome-wide meta-analysis of problematic alcohol use in 435,563 individuals yields insights into biology and relationships with other traits. *Nat Neurosci* 2020;23(7):809-18. doi: 10.1038/s41593-020-0643-5 [published Online First: 2020/05/27] | | | | | | | |
| 4. Mahedy L, Suddell S, Skirrow C, et al. Alcohol use and cognitive functioning in young adults: improving causal inference. *Addiction (Abingdon, England)* 2021;116(2):292-302. doi: 10.1111/add.15100 [published Online First: 2020/04/27] | | | | | | | |
| 5. Mahedy L, Wootton R, Suddell S, et al. Testing the association between tobacco and cannabis use and cognitive functioning: findings from an observational and Mendelian randomization study. *Drug and alcohol dependence* 2021;221:108591. doi: 10.1016/j.drugalcdep.2021.108591 | | | | | | | |
| 6. Gage SH, Sallis HM, Lassi G, et al. Does smoking cause lower educational attainment and general cognitive ability? Triangulation of causal evidence using multiple study designs. *Psychological medicine* 2020;52(8):1578-86. doi: 10.1017/S0033291720003402 | | | | | | | |
| 7. Yang F, Chen S, Qu Z, et al. Genetic Liability to Sedentary Behavior in Relation to Stroke, Its Subtypes and Neurodegenerative Diseases: A Mendelian Randomization Study. *Frontiers in Aging Neuroscience* 2021;13:757388. doi: 10.3389/fnagi.2021.757388 | | | | | | | |
| 8. Hagenaars SP, Gale CR, Deary IJ, et al. Cognitive ability and physical health: a Mendelian randomization study. *Scientific reports* 2017;7(1):2651. doi: 10.1038/s41598-017-02837-3 | | | | | | | |
| 9. Orri M, Pingault JB, Turecki G, et al. Contribution of birth weight to mental health, cognitive and socioeconomic outcomes: Two-sample Mendelian randomisation. *British Journal of Psychiatry* 2021;219(3):507-14. doi: 10.1192/bjp.2021.15 | | | | | | | |
| 10. Sun D, Thomas EA, Launer LJ, et al. Association of blood pressure with cognitive function at midlife: a Mendelian randomization study. *BMC medical genomics* 2020;13(1):121. doi: 10.1186/s12920-020-00769-y | | | | | | | |
| 11. Kwok MK, Schooling CM. Mendelian randomization study on atrial fibrillation and cardiovascular disease subtypes. *Scientific reports* 2021;11(1):18682. doi: 10.1038/s41598-021-98058-w | | | | | | | |
| 12. Higbee DH, Granell R, Hemani G, et al. Lung function, COPD and cognitive function: a multivariable and two sample Mendelian randomization study. *BMC pulmonary medicine* 2021;21(1):246. doi: 10.1186/s12890-021-01611-6 | | | | | | | |
| 13. Ware EB, Morataya C, Fu M, et al. Type 2 Diabetes and Cognitive Status in the Health and Retirement Study: a Mendelian Randomization Approach. *Frontiers in genetics* 2021;12:634767. doi: 10.3389/fgene.2021.634767 | | | | | | | |
| 14. Garfield V, Farmaki AE, Fatemifar G, et al. Relationship Between Glycemia and Cognitive Function, Structural Brain Outcomes, and Dementia: A Mendelian Randomization Study in the UK Biobank. *Diabetes* 2021;70(10):2313-21. doi: 10.2337/db20-0895 [published Online First: 2021/02/27] | | | | | | | |
| 15. Rosoff DB, Kaminsky ZA, McIntosh AM, et al. Educational attainment reduces the risk of suicide attempt among individuals with and without psychiatric disorders independent of cognition: a bidirectional and multivariable Mendelian randomization study with more than 815,000 participants. *Translational psychiatry* 2020;10(1):388. doi: 10.1038/s41398-020-01047-2 | | | | | | | |
| 16. Fitzgerald J, Fahey L, Holleran L, et al. Thirteen Independent Genetic Loci Associated with Preserved Processing Speed in a Study of Cognitive Resilience in 330,097 Individuals in the UK Biobank. *Genes* 2022;13(1):122. doi: 10.3390/genes13010122 | | | | | | | |
| 17. Kwok MK, Schooling CM. Herpes simplex virus and Alzheimer's disease: a Mendelian randomization study. *Neurobiology of aging* 2021;99:101.e11-01.e13. doi: 10.1016/j.neurobiolaging.2020.09.025 | | | | | | | |
| 18. Zhou A, Taylor AE, Karhunen V, et al. Habitual coffee consumption and cognitive function: a Mendelian randomization meta-analysis in up to 415,530 participants. *Scientific reports* 2018;8(1):7526. doi: 10.1038/s41598-018-25919-2 | | | | | | | |
| 19. Maddock J, Zhou A, Cavadino A, et al. Vitamin D and cognitive function: A Mendelian randomisation study. *Sci Rep* 2017;7(1):13230. doi: 10.1038/s41598-017-13189-3 [published Online First: 2017/10/19] | | | | | | | |
| 20. Liu H, Zhang Y, Hu Y, et al. Mendelian randomization to evaluate the effect of plasma vitamin C levels on the risk of Alzheimer’s disease. *Genes and Nutrition* 2021;16(1):19. doi: 10.1186/s12263-021-00700-9 | | | | | | | |
| 21. Fu M, Bakulski KM, Higgins C, et al. Mendelian Randomization of Dyslipidemia on Cognitive Impairment Among Older Americans. *Frontiers in Neurology* 2021;12:660212. doi: 10.3389/fneur.2021.660212 | | | | | | | |
| 22. Dunk MM, Driscoll I. Total Cholesterol and APOE-Related Risk for Alzheimer's Disease in the Alzheimer's Disease Neuroimaging Initiative. *Journal of Alzheimer's disease : JAD* 2021;85(4):1519-28. doi: 10.3233/JAD-215091 | | | | | | | |
| 23. Winchester LM, Powell J, Lovestone S, et al. Red blood cell indices and anaemia as causative factors for cognitive function deficits and for Alzheimer's disease. *Genome medicine* 2018;10(1):51. doi: 10.1186/s13073-018-0556-z | | | | | | | |
| 24. Efstathiadou A, Gill D, McGrane F, et al. Genetically Determined Uric Acid and the Risk of Cardiovascular and Neurovascular Diseases: A Mendelian Randomization Study of Outcomes Investigated in Randomized Trials. *Journal of the American Heart Association* 2019;8(17):e012738. doi: 10.1161/JAHA.119.012738 | | | | | | | |
| 25. Richard E, McEvoy L, Cao S, et al. Biomarkers of kidney function and cognitive ability: a mendelian randomization study. *Circulation* 2021;143:118071. doi: 10.1161/circ.143.suppl_1.028 | | | | | | | |
| 26. Png G, Barysenka A, Repetto L, et al. Mapping the serum proteome to neurological diseases using whole genome sequencing. *Nature communications* 2021;12(1):7042. doi: 10.1038/s41467-021-27387-1 | | | | | | | |
| 27. Hägg S, Zhan Y, Karlsson R, et al. Short telomere length is associated with impaired cognitive performance in European ancestry cohorts. *Translational psychiatry* 2017;7(4):e1100. doi: 10.1038/tp.2017.73 | | | | | | | |
| 28. Caramaschi D, Sharp GC, Nohr EA, et al. Exploring a causal role of DNA methylation in the relationship between maternal vitamin B12 during pregnancy and child's IQ at age 8, cognitive performance and educational attainment: a two-step Mendelian randomization study. *Human molecular genetics* 2017;26(15):3001-13. doi: 10.1093/hmg/ddx164 | | | | | | | |
