# Supplementary material for: Summarizing attributable factors and evaluating risk of bias of Mendelian randomization studies for Alzheimer’s dementia and cognitive status: a systematic review and meta-analysis
Source: Syst Rev. 2025 Mar 13;14:61. doi: 10.1186/s13643-025-02792-5 (PMC11905674; doi:10.1186/s13643-025-02792-5)
Supplement: Supplementary file 7 — Additional file 7. Table S7. Risk of bias assessment for the included MR studies of AD. [file 13643_2025_2792_MOESM7_ESM.docx]

# Table S7 Risk of bias assessment for the included MR studies of AD

| **MR studies** | **1. Bias of instrumental variables selection** | | | **2. Bias of population selection** | | **3. Bias in selection of the reported result** | | | | **Overall judgement** |
| --- | --- | --- | --- | --- | --- | --- | --- | --- | --- | --- |
|  | (1) Weak instrument bias | (2) Pleiotropy bias | (3) Biological Complexity Explained | (1) Crowd stratification | (2) Sample overlap | (1) Consistent with sensitivity analyses | (2) Repeatability | (3) Other research evidence | (4) Reporting bias |  |
| Wang, T. [1] | M | L | L | H | L | L | M | L | L | H |
| Kwok, M. K. [2] | L | L | H | L | M | L | M | L | L | H |
| Li, Q. S. [3] | M | H | L | L | L | L | L | L | L | H |
| Grace, C. [4] | M | L | L | L | L | L | M | L | L | M |
| Han, Z. [5] | M | L | L | L | L | L | M | L | L | M |
| Huang, J. [6] | L | L | L | L | L | L | L | L | L | L |
| Andrews, S. J. [7] | L | L | L | L | L | L | M | L | L | M |
| Jansen, I. E. [8] | M | L | L | L | L | M | M | L | L | M |
| Daghlas, I. [9] | L | M | H | L | M | M | M | L | L | H |
| Higbee, D. [10] | L | H | H | L | L | L | M | L | L | H |
| Russ, T. C. [11] | L | L | H | L | L | L | M | L | L | H |
| Thomassen, J. Q. [12] | L | L | L | L | L | L | M | L | L | M |
| Wang, H. [13] | M | H | L | L | L | L | M | L | L | H |
| Zhang, Q. [14] | M | L | H | L | M | L | M | L | L | H |
| Pan, Y. [15] | M | L | H | L | L | L | M | L | L | H |
| Østergaard, S. D. [16] | M | M | L | L | M | H | M | L | L | H |
| Larsson, S. C. [17] | L | L | L | L | M | L | M | L | L | M |
| Garfield, V. [18] | L | L | H | L | L | L | M | L | L | H |
| Zhuang, Q. S. [19] | M | M | L | L | M | L | M | L | L | M |
| Lee, Y. H. [20] | L | L | H | H | L | L | M | L | L | H |
| Seddighi, S. [21] | M | L | H | L | L | M | M | L | L | H |
| Kwok, M. K. [22] | L | L | H | L | M | L | L | L | L | H |
| Huang, S. Y. [23] | L | L | H | L | L | L | L | L | L | H |
| Sun, Y. Q. [24] | L | L | H | L | M | L | L | L | L | H |
| Policicchio, S. [25] | M | L | H | L | M | L | M | L | L | H |
| Andrews, S. J. [26] | L | M | H | L | L | L | M | L | L | H |
| Bae, S. [27] | M | L | H | H | L | L | M | L | L | H |
| Wu, B. S. [28] | L | L | H | L | H | L | M | L | L | H |
| Yeung, C. H. C. [29] | L | L | L | L | L | L | L | L | L | L |
| Li, X. [30] | L | L | H | L | M | L | M | L | L | H |
| Zhou, Y. [31] | L | L | L | L | L | L | M | L | L | M |
| Ou, Y. N. [32] | M | L | H | L | L | L | M | L | L | H |
| Wang, Z. [33] | L | L | L | L | L | L | M | L | H | H |
| Zhu, Z. [34] | M | L | H | L | M | L | M | L | L | H |
| Sproviero, W. [35] | L | L | H | L | L | L | L | L | L | H |
| Walker, V. M. [36] | M | L | H | L | L | L | M | L | L | H |
| Mukherjee, S. [37] | M | M | M | L | L | L | L | L | L | M |
| Nordestgaard, L. T. [38] | L | M | M | L | M | M | M | L | L | M |
| Anderson, E. L. [39] | L | L | H | L | L | L | M | L | L | H |
| Ko, H. [40] | M | L | L | L | L | L | M | L | L | M |
| Raghavan, N. S. [41] | M | L | L | L | L | L | M | L | L | M |
| Yang, Y. X. [42] | L | L | H | L | L | L | M | L | L | H |
| Li, M. [43] | L | L | L | L | M | L | M | L | L | M |
| Anderson, E. L. [44] | L | L | L | L | L | L | M | L | L | M |
| Henry, A. [45] | L | L | H | L | L | L | L | L | L | H |
| Cullell, N. [46] | L | H | H | L | L | L | M | L | L | H |
| Andrews, S. J [47] | L | L | H | L | L | L | M | L | L | H |
| Ma, Y. H. [48] | L | L | H | L | M | L | M | L | L | H |
| Shen, L. X. [49] | L | L | H | L | H | L | L | L | L | H |
| Baumeister, S. E. [50] | L | L | H | L | L | L | L | L | L | H |
| Wu, P. F. [51] | L | L | L | L | L | L | H | L | L | H |
| Yang, F. [52] | L | L | H | L | L | L | M | L | L | H |
| Zhang, Z. [53] | L | M | H | L | L | M | L | L | L | H |
| Zhang, Z. [54] | M | L | H | L | L | L | L | L | L | H |
| Kwok, M. K. [55] | M | M | L | L | L | M | M | L | L | M |
| Shi, Y. [56] | M | L | H | L | L | L | L | L | L | H |
| He, Y. [57] | L | L | L | L | L | H | M | L | L | H |
| Cheng, W. W. [58] | M | L | H | H | L | L | M | L | L | H |
| Tomata, Y. [59] | L | L | H | L | L | L | M | L | L | H |
| Lord, J. [60] | L | L | L | L | L | L | H | L | L | H |
| Adams, C. D. [61] | L | L | H | L | L | L | M | L | L | H |
| Larsson, S. C. [62] | M | L | L | L | L | L | M | L | L | M |
| Roostaei, T. [63] | M | M | H | L | L | M | L | L | L | H |
| Williams, D. M. [64] | L | L | L | L | L | L | M | L | L | M |
| Gagliano T, S. A. [65] | M | L | L | L | L | L | M | L | L | M |
| Liu, H. [66] | L | L | L | L | L | L | L | L | L | L |
| Chen, L. [67] | L | L | H | L | L | L | M | L | L | H |
| Wang, L. [68] | L | L | H | L | L | L | H | L | L | H |
| Larsson, S. C. [69] | L | L | L | L | L | L | M | L | L | M |
| Mokry, L. E. [70] | L | M | L | L | L | L | M | L | L | M |
| Liu, G. [71] | L | L | H | L | L | L | M | L | L | H |
| Yeung, C. H. C. [72] | L | L | H | L | L | L | L | L | L | H |
| Fani, L. [73] | M | M | L | L | L | L | M | L | L | M |
| Williams, D. M. [74] | M | M | L | L | L | L | L | L | L | M |
| Tsui, A. [75] | L | H | H | L | L | L | M | L | L | H |
| Zhang, Z. [76] | L | L | L | L | M | L | M | L | L | M |
| Handy, A. [77] | L | L | L | L | L | L | M | L | L | M |
| Png, G. [78] | M | M | L | M | L | L | M | L | L | M |
| Rasmussen, K. L. [79] | L | L | L | L | L | M | L | L | L | M |
| Williams, D. M. [80] | M | L | L | L | L | L | L | L | L | M |
| Proitsi, P. [81] | L | M | L | L | L | L | L | L | L | M |
| Benn, M. [82] | L | H | L | M | L | L | M | L | L | H |
| Kjeldsen, E. W. [83] | M | L | L | L | L | L | L | L | L | M |
| Dunk, M. M. [84] | L | M | L | L | L | H | M | L | L | H |
| Yuan, H. [85] | L | L | L | L | L | L | M | L | L | M |
| Zhuang, Z. [86] | L | L | H | L | L | L | M | L | L | H |
| Kunutsor, S. K. [87] | L | L | H | L | L | L | M | L | L | H |
| Zhang, H. [88] | L | L | L | L | L | L | H | L | L | H |
| Li, G. H. [89] | L | L | H | L | L | L | M | L | L | H |
| Romo, M. L. [90] | M | M | H | L | L | L | M | L | L | H |
| Syed, A. A. S. [91] | M | H | L | L | L | H | M | L | L | H |
| Larsson, S. C. [92] | M | M | L | L | L | M | M | L | L | M |
| Pan, Y. [93] | L | L | L | L | L | H | M | L | L | H |
| Walter, S. [94] | M | L | L | L | L | M | L | L | L | M |
| Zhou, M. [95] | M | L | H | L | L | H | M | L | L | H |
| Benn, M. [96] | L | L | L | L | L | L | L | L | L | L |
| Yu, G. [97] | L | L | H | L | M | L | M | L | L | H |
| Scheller M. A. [98] | L | M | L | L | M | L | L | L | L | M |
| Zhan, Y. [99] | M | M | M | M | M | L | M | L | L | M |
| Gao, K. [100] | M | L | L | L | M | L | M | L | L | M |
| Guo, Y. [101] | M | L | H | L | L | L | M | L | L | H |
| Baird, D. A. [102] | M | L | L | L | L | M | M | L | L | M |
| Wingo, A. P. [103] | M | M | L | L | L | M | L | L | L | M |
| Yang, C. [104] | M | M | L | H | L | M | L | L | L | H |
| Zhu, J. [105] | M | M | L | L | L | M | L | L | L | M |
| Liu, D. [106] | M | M | L | L | L | M | L | L | L | M |

AD, Alzheimer’s dementia; L = low risk of bias; H = high risk of bias; M = moderate risk of bias; MR, Mendelian randomization.

**References**

1. Wang T, Ni QB, Wang K, Han Z, Sun BL. Stroke and Alzheimer's Disease: A Mendelian Randomization Study. Frontiers in genetics. 2020;11:581. doi:10.3389/fgene.2020.00581

2. Kwok MK, Schooling CM. Mendelian randomization study on atrial fibrillation and cardiovascular disease subtypes. Scientific reports. 2021;11(1):18682. doi:10.1038/s41598-021-98058-w

3. Li QS, Tian C, Hinds D, Seabrook GR. The association of clinical phenotypes to known AD/FTD genetic risk loci and their inter-relationship. PloS one. 2020;15(11):e0241552. doi:10.1371/journal.pone.0241552

4. Grace C, Clarke R, Goel A, Farrall M, Watkins H, Hopewell JC. Lack of genetic support for shared aetiology of Coronary Artery Disease and Late-onset Alzheimer's disease. Scientific reports. 2018;8(1):7102. doi:10.1038/s41598-018-25460-2

5. Han Z, Tian R, Ren P, et al. Parkinson's disease and Alzheimer's disease: a Mendelian randomization study. BMC medical genetics. 2018;19(Suppl 1):215. doi:10.1186/s12881-018-0721-7

6. Huang J, Zuber V, Matthews PM, Elliott P, Tzoulaki J, Dehghan A. Sleep, major depressive disorder, and Alzheimer disease: A Mendelian randomization study. Neurology. 2020;95(14):e1963-e70. doi:10.1212/WNL.0000000000010463

7. Andrews SJ, Fulton-Howard B, O'Reilly P, Marcora E, Goate AM. Causal Associations Between Modifiable Risk Factors and the Alzheimer's Phenome. Annals of neurology. 2021;89(1):54-65. doi:10.1002/ana.25918

8. Jansen IE, Savage JE, Watanabe K, et al. Genome-wide meta-analysis identifies new loci and functional pathways influencing Alzheimer's disease risk. Nature genetics. 2019;51(3):404-13. doi:10.1038/s41588-018-0311-9

9. Daghlas I, Rist PM, Chasman DI. Effect of genetic liability to migraine on cognition and brain volume: a Mendelian randomization study. Cephalalgia. 2020;40(9):998-1002. doi:10.1177/0333102420916751

10. Higbee D, Granell R, Walton E, Korologou-Linden R, Davey Smith G, Dodd J. Examining the possible causal relationship between lung function, COPD and Alzheimer's disease: a Mendelian randomisation study. BMJ open respiratory research. 2021;8(1):e000759. doi:10.1136/bmjresp-2020-000759

11. Russ TC, Harris SE, Batty GD. Pulmonary Function and Risk of Alzheimer Dementia: Two-Sample Mendelian Randomization Study. Chest. 2021;160(1):274-6. doi:10.1016/j.chest.2020.11.056

12. Thomassen JQ, Tolstrup JS, Benn M, Frikke-Schmidt R. Type-2 diabetes and risk of dementia: observational and Mendelian randomisation studies in 1 million individuals. Epidemiology and psychiatric sciences. 2020;29:e118. doi:10.1017/S2045796020000347

13. Wang H, Rosenthal BS, Makowski C, et al. Causal association of cognitive reserve on Alzheimer's disease with putative sex difference. Alzheimer's and Dementia: Diagnosis, Assessment and Disease Monitoring. 2021;13(1):e12270. doi:10.1002/dad2.12270

14. Zhang Q, Xu F, Wang L, Zhang WD, Sun CQ, Deng HW. Detecting potential causal relationship between multiple risk factors and Alzheimer's disease using multivariable Mendelian randomization. Aging. 2020;12(21):21747‐57. doi:10.18632/aging.103983

15. Pan Y, Chen W, Yan H, Wang M, Xiang X. Glycemic traits and Alzheimer's disease: a Mendelian randomization study. Aging. 2020;12(22):22688-99. doi:10.18632/aging.103887

16. Østergaard SD, Mukherjee S, Sharp SJ, et al. Associations between Potentially Modifiable Risk Factors and Alzheimer Disease: A Mendelian Randomization Study. PLoS medicine. 2015;12(6):e1001841. doi:10.1371/journal.pmed.1001841

17. Larsson SC, Traylor M, Malik R, Dichgans M, Burgess S, Markus HS. Modifiable pathways in Alzheimer's disease: Mendelian randomisation analysis. BMJ (Clinical research ed.). 2017;359:j5375. doi:10.1136/bmj.j5375

18. Garfield V, Farmaki AE, Fatemifar G, et al. Relationship Between Glycemia and Cognitive Function, Structural Brain Outcomes, and Dementia: A Mendelian Randomization Study in the UK Biobank. Diabetes. 2021;70(10):2313-21. doi:10.2337/db20-0895

19. Zhuang QS, Meng L, Wang Z, Shen L, Ji HF. Associations Between Obesity and Alzheimer's Disease: Multiple Bioinformatic Analyses. J Alzheimers Dis. 2021;80(1):271-81. doi:10.3233/jad-201235

20. Lee YH. Gout and the risk of Alzheimer's disease: A Mendelian randomization study. International journal of rheumatic diseases. 2019;22(6):1046-51. doi:10.1111/1756-185X.13548

21. Seddighi S, Houck AL, Rowe JB, Pharoah PDP. Evidence of a Causal Association Between Cancer and Alzheimer's Disease: a Mendelian Randomization Analysis. Scientific reports. 2019;9(1):13548. doi:10.1038/s41598-019-49795-6

22. Kwok MK, Schooling CM. Herpes simplex virus and Alzheimer's disease: a Mendelian randomization study. Neurobiology of aging. 2021;99:101.e11-.e13. doi:10.1016/j.neurobiolaging.2020.09.025

23. Huang SY, Yang YX, Kuo K, et al. Herpesvirus infections and Alzheimer's disease: a Mendelian randomization study. Alzheimer's research & therapy. 2021;13(1):158. doi:10.1186/s13195-021-00905-5

24. Sun YQ, Richmond RC, Chen Y, Mai XM. Mixed evidence for the relationship between periodontitis and Alzheimer's disease: A bidirectional Mendelian randomization study. PLoS One. 2020;15(1):e0228206. doi:10.1371/journal.pone.0228206

25. Policicchio S, Ahmad AN, Powell JF, Proitsi P. Rheumatoid arthritis and risk for Alzheimer's disease: a systematic review and meta-analysis and a Mendelian Randomization study. Scientific reports. 2017;7(1):12861. doi:10.1038/s41598-017-13168-8

26. Andrews SJ, Goate A. Mendelian randomization indicates that TNF is not causally associated with Alzheimer's disease. Neurobiology of aging. 2019. doi:10.1016/j.neurobiolaging.2019.09.003

27. Bae SC, Lee YH. Causal association between rheumatoid arthritis and a decreased risk of Alzheimer's disease : A Mendelian randomization study. Zeitschrift fur Rheumatologie. 2019;78(4):359-64. doi:10.1007/s00393-018-0504-8

28. Wu BS, Zhang YR, Li HQ, et al. Cortical structure and the risk for Alzheimer's disease: a bidirectional Mendelian randomization study. Translational psychiatry. 2021;11(1):476. doi:10.1038/s41398-021-01599-x

29. Yeung CHC, Lau KWD, Au Yeung SL, Schooling CM. Amyloid, tau and risk of Alzheimer's disease: a Mendelian randomization study. European journal of epidemiology. 2021;36(1):81-8. doi:10.1007/s10654-020-00683-8

30. Li X, Tian Y, Yang YX, et al. Life Course Adiposity and Alzheimer's Disease: A Mendelian Randomization Study. Journal of Alzheimer's disease : JAD. 2021;82(2):503-12. doi:10.3233/jad-210345

31. Zhou Y, Sun X, Zhou M. Body Shape and Alzheimer’s Disease: A Mendelian Randomization Analysis. Frontiers in Neuroscience. 2019;13:1084. doi:10.3389/fnins.2019.01084

32. Ou YN, Yang YX, Shen XN, et al. Genetically determined blood pressure, antihypertensive medications, and risk of Alzheimer's disease: a Mendelian randomization study. Alzheimer's research & therapy. 2021;13(1):41. doi:10.1186/s13195-021-00782-y

33. Wang Z, Meng L, Shen L, Ji H-F. Impact of modifiable risk factors on Alzheimer's disease: A two-sample Mendelian randomization study. Neurobiology of aging. 2020;91:167.e11-.e19. doi:10.1016/j.neurobiolaging.2020.02.018

34. Zhu Z, Zheng Z, Zhang F, et al. Causal associations between risk factors and common diseases inferred from GWAS summary data. Nature communications. 2018;9(1):224. doi:10.1038/s41467-017-02317-2

35. Sproviero W, Winchester L, Newby D, et al. High Blood Pressure and Risk of Dementia: A Two-Sample Mendelian Randomization Study in the UK Biobank. Biological psychiatry. 2021;89(8):817-24. doi:10.1016/j.biopsych.2020.12.015

36. Walker VM, Kehoe PG, Martin RM, Davies NM. Repurposing antihypertensive drugs for the prevention of Alzheimer's disease: a Mendelian randomization study. International journal of epidemiology. 2020;49(4):1132-40. doi:10.1093/ije/dyz155

37. Mukherjee S, Walter S, Kauwe JSK, et al. Genetically predicted body mass index and Alzheimer's disease-related phenotypes in three large samples: Mendelian randomization analyses. Alzheimer's & dementia : the journal of the Alzheimer's Association. 2015;11(12):1439-51. doi:10.1016/j.jalz.2015.05.015

38. Nordestgaard LT, Tybjærg-Hansen A, Nordestgaard BG, Frikke-Schmidt R. Body Mass Index and Risk of Alzheimer's Disease: A Mendelian Randomization Study of 399,536 Individuals. The Journal of clinical endocrinology and metabolism. 2017;102(7):2310-20. doi:10.1210/jc.2017-00195

39. Anderson EL, Howe LD, Wade KH, et al. Education, intelligence and Alzheimer's disease: evidence from a multivariable two-sample Mendelian randomization study. International journal of epidemiology. 2020;49(4):1163-72. doi:10.1093/ije/dyz280

40. Ko H, Kim S, Kim K, et al. Genome-wide association study of occupational attainment as a proxy for cognitive reserve. Brain : a journal of neurology. 2021;145(4):1436-48. doi:10.1093/brain/awab351

41. Raghavan NS, Vardarajan B, Mayeux R. Genomic variation in educational attainment modifies Alzheimer disease risk. Neurology: Genetics. 2019;5(2):e310. doi:10.1212/NXG.0000000000000310

42. Yang YX, Kuo K, Li HQ, et al. Investigating Causal Relations Between Risk Tolerance, Risky Behaviors, and Alzheimer's Disease: A Bidirectional Two-Sample Mendelian Randomization Study. Journal of Alzheimer's disease : JAD. 2020;78(4):1679-87. doi:10.3233/jad-200773

43. Li M, Lin J, Liang S, et al. The role of age at menarche and age at menopause in Alzheimer's disease: evidence from a bidirectional mendelian randomization study. Aging. 2021;13(15):19722-49. doi:10.18632/aging.203384

44. Anderson EL, Richmond RC, Jones SE, et al. Is disrupted sleep a risk factor for Alzheimer's disease? Evidence from a two-sample Mendelian randomization analysis. International journal of epidemiology. 2021;50(3):817-28. doi:10.1093/ije/dyaa183

45. Henry A, Katsoulis M, Masi S, et al. The relationship between sleep duration, cognition and dementia: a Mendelian randomization study. International journal of epidemiology. 2019;48(3):849-60. doi:10.1093/ije/dyz071

46. Cullell N, Carcel-Marquez J, Gallego-Fabrega C, et al. Sleep/wake cycle alterations as a cause of neurodegenerative diseases: a Mendelian randomization study. Neurobiology of aging. 2021;106:320.e1-.e12. doi:10.1016/j.neurobiolaging.2021.05.008

47. Andrews SJ, Goate A, Anstey KJ. Association between alcohol consumption and Alzheimer's disease: A Mendelian randomization study. Alzheimer's & dementia : the journal of the Alzheimer's Association. 2020;16(2):345-53. doi:10.1016/j.jalz.2019.09.086

48. Ma YH, Yang YX, Shen XN, et al. Evaluation relationships between subjective wellbeing, personality traits, and Alzheimer's disease: a two-sample Mendelian randomization study. Journal of psychiatric research. 2021;137:498‐505. doi:10.1016/j.jpsychires.2021.03.033

49. Shen LX, Yang YX, Kuo K, et al. Social Isolation, Social Interaction, and Alzheimer's Disease: a Mendelian Randomization Study. Journal of Alzheimer's disease. 2021;80(2):665‐72. doi:10.3233/JAD-201442

50. Baumeister SE, Karch A, Bahls M, Teumer A, Leitzmann MF, Baurecht H. Physical activity and risk of Alzheimer's disease: a two-sample Mendelian randomization study. Neurology. 2020;95(13):e1897-e905. doi:10.1212/WNL.0000000000010013

51. Wu PF, Lu H, Zhou X, et al. Assessment of causal effects of physical activity on neurodegenerative diseases: A Mendelian randomization study. Journal of sport and health science. 2021;10(4):454-61. doi:10.1016/j.jshs.2021.01.008

52. Yang F, Chen S, Qu Z, Wang K, Xie X, Cui H. Genetic Liability to Sedentary Behavior in Relation to Stroke, Its Subtypes and Neurodegenerative Diseases: A Mendelian Randomization Study. Frontiers in Aging Neuroscience. 2021;13:757388. doi:10.3389/fnagi.2021.757388

53. Zhang Z, Wang M, Yuan S, Larsson SC, Liu X. Genetically predicted milk intake and risk of neurodegenerative diseases. Nutrients. 2021;13(8):2893. doi:10.3390/nu13082893

54. Zhang Z, Wang M, Yuan S, Cai H, Zhu SG, Liu X. Genetically Predicted Coffee Consumption and Risk of Alzheimer's Disease and Stroke. Journal of Alzheimer's disease. 2021;83(4):1815-23. doi:10.3233/JAD-210678

55. Kwok MK, Leung GM, Schooling CM. Habitual coffee consumption and risk of type 2 diabetes, ischemic heart disease, depression and Alzheimer's disease: a Mendelian randomization study. Scientific reports. 2016;6:36500. doi:10.1038/srep36500

56. Shi Y, Liu R, Guo Y, et al. An Updated Mendelian Randomization Analysis of the Association Between Serum Calcium Levels and the Risk of Alzheimer's Disease. Frontiers in genetics. 2021;12:731391. doi:10.3389/fgene.2021.731391

57. He Y, Zhang H, Wang T, et al. Impact of Serum Calcium Levels on Alzheimer's Disease: a Mendelian Randomization Study. Journal of Alzheimer's disease. 2020;76(2):713‐24. doi:10.3233/JAD-191249

58. Cheng WW, Zhu Q, Zhang HY. Mineral Nutrition and the Risk of Chronic Diseases: A Mendelian Randomization Study. Nutrients. 2019;11(2):378. doi:10.3390/nu11020378

59. Tomata Y, Larsson SC, Hägg S. Polyunsaturated fatty acids and risk of Alzheimer's disease: a Mendelian randomization study. European journal of nutrition. 2020;59(4):1763-6. doi:10.1007/s00394-019-02126-x

60. Lord J, Jermy B, Green R, et al. Mendelian randomization identifies blood metabolites previously linked to midlife cognition as causal candidates in Alzheimer's disease. Proceedings of the National Academy of Sciences of the United States of America. 2021;118(16):e2009808118. doi:10.1073/pnas.2009808118

61. Adams CD. Circulating Glutamine and Alzheimer's Disease: A Mendelian Randomization Study. Clinical interventions in aging. 2020;15:185-93. doi:10.2147/CIA.S239350

62. Larsson SC, Markus HS. Branched-chain amino acids and Alzheimer's disease: a Mendelian randomization analysis. Scientific reports. 2017;7(1):13604. doi:10.1038/s41598-017-12931-1

63. Roostaei T, Felsky D, Nazeri A, et al. Genetic influence of plasma homocysteine on Alzheimer's disease. Neurobiology of aging. 2018;62:243.e7-.e14. doi:10.1016/j.neurobiolaging.2017.09.033

64. Williams DM, Hägg S, Pedersen NL. Circulating antioxidants and Alzheimer disease prevention: a Mendelian randomization study. The American journal of clinical nutrition. 2019;109(1):90-8. doi:10.1093/ajcn/nqy225

65. Gagliano Taliun SA. Genetic determinants of low vitamin B12 levels in Alzheimer's disease risk. Alzheimer's & dementia (Amsterdam, Netherlands). 2019;11:430-4. doi:10.1016/j.dadm.2019.04.007

66. Liu H, Zhang Y, Hu Y, et al. Mendelian randomization to evaluate the effect of plasma vitamin C levels on the risk of Alzheimer’s disease. Genes and Nutrition. 2021;16(1):19. doi:10.1186/s12263-021-00700-9

67. Chen L, Sun X, Wang Z, et al. The impact of plasma vitamin C levels on the risk of cardiovascular diseases and Alzheimer's disease: A Mendelian randomization study. Clinical nutrition (Edinburgh, Scotland). 2021;40(10):5327-34. doi:10.1016/j.clnu.2021.08.020

68. Wang L, Qiao Y, Zhang H, et al. Circulating Vitamin D Levels and Alzheimer's Disease: A Mendelian Randomization Study in the IGAP and UK Biobank. Journal of Alzheimer's disease : JAD. 2020;73(2):609-18. doi:10.3233/JAD-190713

69. Larsson SC, Traylor M, Markus HS, Michaëlsson K. Serum Parathyroid Hormone, 25-Hydroxyvitamin D, and Risk of Alzheimer's Disease: A Mendelian Randomization Study. Nutrients. 2018;10(9):1243. doi:10.3390/nu10091243

70. Mokry LE, Ross S, Morris JA, Manousaki D, Forgetta V, Richards JB. Genetically decreased vitamin D and risk of Alzheimer disease. Neurology. 2016;87(24):2567-74. doi:10.1212/WNL.0000000000003430

71. Liu G, Zhao Y, Jin S, et al. Circulating vitamin E levels and Alzheimer's disease: a Mendelian randomization study. Neurobiology of aging. 2018;72:189.e1-.e9. doi:10.1016/j.neurobiolaging.2018.08.008

72. Yeung CHC, Schooling CM. Systemic inflammatory regulators and risk of Alzheimer's disease: a bidirectional Mendelian-randomization study. International journal of epidemiology. 2021;50(3):829-40. doi:10.1093/ije/dyaa241

73. Fani L, Georgakis MK, Ikram MA, Ikram MK, Malik R, Dichgans M. Circulating biomarkers of immunity and inflammation, risk of Alzheimer's disease, and hippocampal volume: a Mendelian randomization study. Translational psychiatry. 2021;11(1):291. doi:10.1038/s41398-021-01400-z

74. Williams DM, Karlsson IK, Pedersen NL, Hägg S. Circulating insulin-like growth factors and Alzheimer disease: A mendelian randomization study. Neurology. 2018;90(4):e291-e7. doi:10.1212/WNL.0000000000004854

75. Tsui A, Davis D. Systemic inflammation and causal risk for Alzheimer's dementia: Possibilities and limitations of a Mendelian randomization approach. Aging medicine (Milton (N.S.W)). 2018;1(3):249-53. doi:10.1002/agm2.12046

76. Zhang Z, Wang M, Liu X. C-reactive protein and risk of Alzheimer's disease. Neurobiology of aging. 2022;109:259-63. doi:10.1016/j.neurobiolaging.2021.08.010

77. Handy A, Lord J, Green R, et al. Assessing Genetic Overlap and Causality Between Blood Plasma Proteins and Alzheimer's Disease. Journal of Alzheimer's disease. 2021;83(4):1825-39. doi:10.3233/JAD-210462

78. Png G, Barysenka A, Repetto L, et al. Mapping the serum proteome to neurological diseases using whole genome sequencing. Nature communications. 2021;12(1):7042. doi:10.1038/s41467-021-27387-1

79. Rasmussen KL, Nordestgaard BG, Frikke-Schmidt R, Nielsen SF. An updated Alzheimer hypothesis: complement C3 and risk of Alzheimer's disease-A cohort study of 95,442 individuals. Alzheimer's & dementia. 2018;14(12):1589‐601. doi:10.1016/j.jalz.2018.07.223

80. Williams DM, Finan C, Schmidt AF, Burgess S, Hingorani AD. Lipid lowering and Alzheimer disease risk: A mendelian randomization study. Annals of neurology. 2020;87(1):30-9. doi:10.1002/ana.25642

81. Proitsi P, Lupton MK, Velayudhan L, et al. Genetic Predisposition to Increased Blood Cholesterol and Triglyceride Lipid Levels and Risk of Alzheimer Disease: A Mendelian Randomization Analysis. PLoS medicine. 2014;11(9):e1001713. doi:10.1371/journal.pmed.1001713

82. Benn M, Nordestgaard BG, Frikke-Schmidt R, Tybjærg-Hansen A. Low LDL cholesterol, PCSK9 and HMGCR genetic variation, and risk of Alzheimer's disease and Parkinson's disease: Mendelian randomisation study. BMJ (Clinical research ed.). 2017;357:j1648. doi:10.1136/bmj.j1648

83. Kjeldsen EW, Thomassen JQ, Juul Rasmussen I, Nordestgaard BG, Tybjaerg-Hansen A, Frikke-Schmidt R. Plasma HDL cholesterol and risk of dementia - observational and genetic studies. Cardiovascular research. 2021;118(5):1330-43. doi:10.1093/cvr/cvab164

84. Dunk MM, Driscoll I. Total Cholesterol and APOE-Related Risk for Alzheimer's Disease in the Alzheimer's Disease Neuroimaging Initiative. Journal of Alzheimer's disease : JAD. 2021;85(4):1519-28. doi:10.3233/JAD-215091

85. Yuan H, Yang W. Genetically Determined Serum Uric Acid and Alzheimer's Disease Risk. Journal of Alzheimer's disease : JAD. 2018;65(4):1259-65. doi:10.3233/JAD-180538

86. Zhuang Z, Yang R, Wang W, Qi L, Huang T. Associations between gut microbiota and Alzheimer's disease, major depressive disorder, and schizophrenia. Journal of neuroinflammation. 2020;17(1):288. doi:10.1186/s12974-020-01961-8

87. Kunutsor SK, Laukkanen JA, Burgess S. Genetically elevated gamma-glutamyltransferase and Alzheimer's disease. Experimental gerontology. 2018;106:61-6. doi:10.1016/j.exger.2018.03.001

88. Zhang H, Wang T, Han Z, et al. Impact of Vitamin D Binding Protein Levels on Alzheimer's Disease: a Mendelian Randomization Study. Journal of Alzheimer's disease. 2020;74(3):991-8. doi:10.3233/JAD-191051

89. Li GH, Cheung CL, Cheung EY, Chan WC, Tan KC. Genetically Determined TSH Level Within Reference Range Is Inversely Associated With Alzheimer Disease. J Clin Endocrinol Metab. 2021;106(12):e5064-e74. doi:10.1210/clinem/dgab527

90. Romo ML, Schooling CM. Examining the Causal Role of Leptin in Alzheimer Disease: a Mendelian Randomization Study. Neuroendocrinology. 2017;105(2):182‐8. doi:10.1159/000475713

91. Syed AAS, He L, Shi Y. The Potential Effect of Aberrant Testosterone Levels on Common Diseases: A Mendelian Randomization Study. Genes. 2020;11(7):721. doi:10.3390/genes11070721

92. Larsson SC, Gill D, Mason AM, et al. Lipoprotein(a) in Alzheimer, Atherosclerotic, Cerebrovascular, Thrombotic, and Valvular Disease: mendelian Randomization Investigation. Circulation. 2020:1826‐8. doi:10.1161/CIRCULATIONAHA.120.045826

93. Pan Y, Li H, Wang Y, Meng X, Wang Y. Causal Effect of Lp(a) [Lipoprotein(a)] Level on Ischemic Stroke and Alzheimer Disease: a Mendelian Randomization Study. Stroke; a journal of cerebral circulation. 2019;50(12):3532-9. doi:10.1161/STROKEAHA.119.026872

94. Walter S, Marden JR, Kubzansky LD, et al. Diabetic Phenotypes and Late-Life Dementia Risk: A Mechanism-specific Mendelian Randomization Study. Alzheimer disease and associated disorders. 2016;30(1):15-20. doi:10.1097/wad.0000000000000128

95. Zhou M, Li H, Wang Y, Pan Y, Wang Y. Causal effect of insulin resistance on small vessel stroke and Alzheimer's disease: A Mendelian randomization analysis. European Journal of Neurology. 2021;29(3):698-706. doi:10.1111/ene.15190

96. Benn M, Nordestgaard BG, Tybjærg-Hansen A, Frikke-Schmidt R. Impact of glucose on risk of dementia: Mendelian randomisation studies in 115,875 individuals. Diabetologia. 2020;63(6):1151-61. doi:10.1007/s00125-020-05124-5

97. Yu G, Lu L, Ma Z, Wu S. Genetically Predicted Telomere Length and Its Relationship With Alzheimer’s Disease. Frontiers in genetics. 2021;12:595864. doi:10.3389/fgene.2021.595864

98. Scheller Madrid A, Rasmussen KL, Rode L, Frikke-Schmidt R, Nordestgaard BG, Bojesen SE. Observational and genetic studies of short telomeres and Alzheimer's disease in 67,000 and 152,000 individuals: a Mendelian randomization study. European journal of epidemiology. 2020;35(2):147-56. doi:10.1007/s10654-019-00563-w

99. Zhan Y, Song C, Karlsson R, et al. Telomere Length Shortening and Alzheimer Disease--A Mendelian Randomization Study. JAMA neurology. 2015;72(10):1202-3. doi:10.1001/jamaneurol.2015.1513

100. Gao K, Wei C, Zhu J, et al. Exploring the Causal Pathway From Telomere Length to Alzheimer's Disease: An Update Mendelian Randomization Study. Frontiers in psychiatry. 2019;10:843. doi:10.3389/fpsyt.2019.00843

101. Guo Y, Yu H. Leukocyte Telomere Length Shortening and Alzheimer's Disease Etiology. Journal of Alzheimer's disease : JAD. 2019;69(3):881-5. doi:10.3233/JAD-190134

102. Baird DA, Liu JZ, Zheng J, et al. Identifying drug targets for neurological and psychiatric disease via genetics and the brain transcriptome. PLoS genetics. 2021;17(1):e1009224. doi:10.1371/journal.pgen.1009224

103. Wingo AP, Liu Y, Gerasimov ES, et al. Integrating human brain proteomes with genome-wide association data implicates new proteins in Alzheimer's disease pathogenesis. Nature genetics. 2021;53(2):143-6. doi:10.1038/s41588-020-00773-z

104. Yang C, Farias FHG, Ibanez L, et al. Genomic atlas of the proteome from brain, CSF and plasma prioritizes proteins implicated in neurological disorders. Nature neuroscience. 2021;24(9):1302-12. doi:10.1038/s41593-021-00886-6

105. Zhu J, Liu X, Yin H, Gao Y, Yu H. Convergent lines of evidence support BIN1 as a risk gene of Alzheimer’s disease. Human genomics. 2021;15(1):9. doi:10.1186/s40246-021-00307-6

106. Liu D, Wang Y, Jing H, Meng Q, Yang J. Novel DNA methylation loci and genes showing pleiotropic association with Alzheimer's dementia: a network Mendelian randomization analysis. Epigenetics. 2021;17(7):746-58. doi:10.1080/15592294.2021.1959735
