# Supplementary material for: Summarizing attributable factors and evaluating risk of bias of Mendelian randomization studies for Alzheimer’s dementia and cognitive status: a systematic review and meta-analysis
Source: Syst Rev. 2025 Mar 13;14:61. doi: 10.1186/s13643-025-02792-5 (PMC11905674; doi:10.1186/s13643-025-02792-5)
Supplement: Supplementary file 8 — Additional file 8. Table S8. Risk of bias assessment for the included MR studies of cognitive status. [file 13643_2025_2792_MOESM8_ESM.docx]

# Table S8 Risk of bias assessment for the included MR studies of cognitive status

| **MR studies** | **1. Bias of instrumental variables selection** | | | **2. Bias of population selection** | | **3. Bias in selection of the reported result** | | | | **Overall judgement** |
| --- | --- | --- | --- | --- | --- | --- | --- | --- | --- | --- |
|  | (1) Weak instrument bias | (2) Pleiotropy bias | (3) Biological Complexity Explained | (1) Crowd stratification | (2) Sample overlap | (1) Consistent with sensitivity analyses | (2) Repeatability | (3) Other research evidence | (4) Reporting bias |  |
| Li, M. [1] | L | L | L | L | M | L | M | L | L | M |
| Zhou, H. [2] | M | L | H | L | L | L | M | L | L | H |
| Mahedy, L. [3] | M | M | H | L | L | L | L | L | L | H |
| Mahedy, L. [4] | L | M | H | L | L | L | L | L | L | H |
| Gage, S. H. [5] | M | L | H | L | L | L | L | L | L | H |
| Yang, F. [6] | L | L | H | L | M | L | M | L | L | H |
| Zhou, A. [7] | L | L | L | L | L | L | M | L | L | M |
| Henry, A. [8] | L | L | H | L | L | L | L | L | L | H |
| Rosoff, D. B. [9] | L | L | H | L | L | L | M | L | L | H |
| Ware, E. B. [10] | M | M | L | L | L | L | L | L | L | M |
| Hagenaars, S. P. [11] | L | M | H | L | L | M | M | L | L | H |
| Garfield, V. [12] | L | L | H | L | L | L | M | L | L | H |
| Kwok, M. K. [13] | L | L | H | L | M | L | M | L | L | H |
| Fitzgerald, J. [14] | M | M | L | L | L | M | L | L | L | M |
| Kwok, M. K. [15] | L | L | H | L | M | L | L | L | L | H |
| Higbee, D. H. [16] | L | L | H | L | L | L | M | L | L | H |
| Richard, E. [17] | L | L | H | L | L | L | M | L | L | H |
| Sun, D. [18] | M | L | H | L | L | L | M | L | L | H |
| Orri, M. [19] | L | L | H | L | L | L | M | L | L | H |
| Fu, M. [20] | M | M | L | L | L | L | M | L | L | M |
| Dunk, M. M. [21] | L | M | L | L | L | H | M | L | L | H |
| Maddock, J. [22] | L | M | L | L | L | M | M | L | L | M |
| Hägg, S. [23] | M | M | L | L | L | L | L | L | L | M |
| Winchester, L. M. [24] | M | L | H | L | L | L | L | L | L | H |
| Efstathiadou, A. [25] | L | L | H | L | L | L | M | L | L | H |
| Caramaschi. D. [26] | M | M | L | L | L | M | L | L | L | M |
| Png, G. [27] | M | M | L | M | L | L | M | L | L | M |
| Liu, H. [28] | L | L | L | L | L | L | M | L | L | M |

L = low risk of bias; H = high risk of bias; M = moderate risk of bias; MR, Mendelian randomization.

**References**

1. Li M, Lin J, Liang S, et al. The role of age at menarche and age at menopause in Alzheimer's disease: evidence from a bidirectional mendelian randomization study. Aging. 2021;13(15):19722-49. doi:10.18632/aging.203384

2. Zhou H, Sealock JM, Sanchez-Roige S, et al. Genome-wide meta-analysis of problematic alcohol use in 435,563 individuals yields insights into biology and relationships with other traits. Nat Neurosci. 2020;23(7):809-18. doi:10.1038/s41593-020-0643-5

3. Mahedy L, Suddell S, Skirrow C, et al. Alcohol use and cognitive functioning in young adults: improving causal inference. Addiction (Abingdon, England). 2021;116(2):292-302. doi:10.1111/add.15100

4. Mahedy L, Wootton R, Suddell S, et al. Testing the association between tobacco and cannabis use and cognitive functioning: findings from an observational and Mendelian randomization study. Drug and alcohol dependence. 2021;221:108591. doi:10.1016/j.drugalcdep.2021.108591

5. Gage SH, Sallis HM, Lassi G, et al. Does smoking cause lower educational attainment and general cognitive ability? Triangulation of causal evidence using multiple study designs. Psychological medicine. 2020;52(8):1578-86. doi:10.1017/S0033291720003402

6. Yang F, Chen S, Qu Z, Wang K, Xie X, Cui H. Genetic Liability to Sedentary Behavior in Relation to Stroke, Its Subtypes and Neurodegenerative Diseases: A Mendelian Randomization Study. Frontiers in Aging Neuroscience. 2021;13:757388. doi:10.3389/fnagi.2021.757388

7. Zhou A, Taylor AE, Karhunen V, et al. Habitual coffee consumption and cognitive function: a Mendelian randomization meta-analysis in up to 415,530 participants. Scientific reports. 2018;8(1):7526. doi:10.1038/s41598-018-25919-2

8. Henry A, Katsoulis M, Masi S, et al. The relationship between sleep duration, cognition and dementia: a Mendelian randomization study. International journal of epidemiology. 2019;48(3):849-60. doi:10.1093/ije/dyz071

9. Rosoff DB, Kaminsky ZA, McIntosh AM, Davey Smith G, Lohoff FW. Educational attainment reduces the risk of suicide attempt among individuals with and without psychiatric disorders independent of cognition: a bidirectional and multivariable Mendelian randomization study with more than 815,000 participants. Translational psychiatry. 2020;10(1):388. doi:10.1038/s41398-020-01047-2

10. Ware EB, Morataya C, Fu M, Bakulski KM. Type 2 Diabetes and Cognitive Status in the Health and Retirement Study: a Mendelian Randomization Approach. Frontiers in genetics. 2021;12:634767. doi:10.3389/fgene.2021.634767

11. Hagenaars SP, Gale CR, Deary IJ, Harris SE. Cognitive ability and physical health: a Mendelian randomization study. Scientific reports. 2017;7(1):2651. doi:10.1038/s41598-017-02837-3

12. Garfield V, Farmaki AE, Fatemifar G, et al. Relationship Between Glycemia and Cognitive Function, Structural Brain Outcomes, and Dementia: A Mendelian Randomization Study in the UK Biobank. Diabetes. 2021;70(10):2313-21. doi:10.2337/db20-0895

13. Kwok MK, Schooling CM. Mendelian randomization study on atrial fibrillation and cardiovascular disease subtypes. Scientific reports. 2021;11(1):18682. doi:10.1038/s41598-021-98058-w

14. Fitzgerald J, Fahey L, Holleran L, Broin PÓ, Donohoe G, Morris DW. Thirteen Independent Genetic Loci Associated with Preserved Processing Speed in a Study of Cognitive Resilience in 330,097 Individuals in the UK Biobank. Genes. 2022;13(1):122. doi:10.3390/genes13010122

15. Kwok MK, Schooling CM. Herpes simplex virus and Alzheimer's disease: a Mendelian randomization study. Neurobiology of aging. 2021;99:101.e11-.e13. doi:10.1016/j.neurobiolaging.2020.09.025

16. Higbee DH, Granell R, Hemani G, Smith GD, Dodd JW. Lung function, COPD and cognitive function: a multivariable and two sample Mendelian randomization study. BMC pulmonary medicine. 2021;21(1):246. doi:10.1186/s12890-021-01611-6

17. Richard E, McEvoy L, Cao S, Lacroix AZ, Salem R. Biomarkers of kidney function and cognitive ability: a mendelian randomization study. Circulation. 2021;143:118071. doi:10.1161/circ.143.suppl_1.028

18. Sun D, Thomas EA, Launer LJ, Sidney S, Yaffe K, Fornage M. Association of blood pressure with cognitive function at midlife: a Mendelian randomization study. BMC medical genomics. 2020;13(1):121. doi:10.1186/s12920-020-00769-y

19. Orri M, Pingault JB, Turecki G, et al. Contribution of birth weight to mental health, cognitive and socioeconomic outcomes: Two-sample Mendelian randomisation. British Journal of Psychiatry. 2021;219(3):507-14. doi:10.1192/bjp.2021.15

20. Fu M, Bakulski KM, Higgins C, Ware EB. Mendelian Randomization of Dyslipidemia on Cognitive Impairment Among Older Americans. Frontiers in Neurology. 2021;12:660212. doi:10.3389/fneur.2021.660212

21. Dunk MM, Driscoll I. Total Cholesterol and APOE-Related Risk for Alzheimer's Disease in the Alzheimer's Disease Neuroimaging Initiative. Journal of Alzheimer's disease : JAD. 2021;85(4):1519-28. doi:10.3233/JAD-215091

22. Maddock J, Zhou A, Cavadino A, et al. Vitamin D and cognitive function: A Mendelian randomisation study. Sci Rep. 2017;7(1):13230. doi:10.1038/s41598-017-13189-3

23. Hägg S, Zhan Y, Karlsson R, et al. Short telomere length is associated with impaired cognitive performance in European ancestry cohorts. Translational psychiatry. 2017;7(4):e1100. doi:10.1038/tp.2017.73

24. Winchester LM, Powell J, Lovestone S, Nevado-Holgado AJ. Red blood cell indices and anaemia as causative factors for cognitive function deficits and for Alzheimer's disease. Genome medicine. 2018;10(1):51. doi:10.1186/s13073-018-0556-z

25. Efstathiadou A, Gill D, McGrane F, Quinn T, Dawson J. Genetically Determined Uric Acid and the Risk of Cardiovascular and Neurovascular Diseases: A Mendelian Randomization Study of Outcomes Investigated in Randomized Trials. Journal of the American Heart Association. 2019;8(17):e012738. doi:10.1161/JAHA.119.012738

26. Caramaschi D, Sharp GC, Nohr EA, et al. Exploring a causal role of DNA methylation in the relationship between maternal vitamin B12 during pregnancy and child's IQ at age 8, cognitive performance and educational attainment: a two-step Mendelian randomization study. Human molecular genetics. 2017;26(15):3001-13. doi:10.1093/hmg/ddx164

27. Png G, Barysenka A, Repetto L, et al. Mapping the serum proteome to neurological diseases using whole genome sequencing. Nature communications. 2021;12(1):7042. doi:10.1038/s41467-021-27387-1

28. Liu H, Zhang Y, Hu Y, et al. Mendelian randomization to evaluate the effect of plasma vitamin C levels on the risk of Alzheimer’s disease. Genes and Nutrition. 2021;16(1):19. doi:10.1186/s12263-021-00700-9
